# Prevalence and Risk Factors of Coxiellosis at the Human–Animal–Environment Interface in the South Asian Countries: A Systematic Review and Meta-Analysis

**DOI:** 10.1155/tbed/2890693

**Published:** 2025-01-31

**Authors:** Md Mazharul Islam, Pronesh Dutta, Devendra Bansal, Gyanendra Gongal, Elmoubashar Farag, Ricardo J. Soares Magalhaes, John I. Alawneh, Jane Heller, Mohammad Mahmudul Hassan

**Affiliations:** ^1^Department of Animal Resources, Ministry of Municipality, Doha, Qatar; ^2^Institute of Epidemiology, Disease Control and Research, Mohakhali, Dhaka 1212, Bangladesh; ^3^Department of Health Protection and Communicable Diseases, Ministry of Public Health, Doha, Qatar; ^4^World Health Emergency Program, WHO Regional Office for South-East Asia, New Delhi, India; ^5^Queensland Alliance for One Health Sciences, School of Veterinary Science, The University of Queensland, Brisbane 4343, Queensland, Australia; ^6^Children Health and Environment Program, UQ Child Health Research Centre, The University of Queensland, Brisbane, Queensland 4343, Australia; ^7^Plant Biosecurity and Product Integrity, Biosecurity Queensland, Department of Primary Industries, Brisbane 4000, Queensland, Australia; ^8^School of Agricultural, Environmental, and Veterinary Sciences, Charles Sturt University, Wagga Wagga 2678, New South Wales, Australia; ^9^Faculty of Veterinary Medicine, Chattogram Veterinary and Animal Sciences University, Chattogram 4225, Bangladesh

**Keywords:** *Coxiella burnetii*, One Health, prevalence, risk factors, South Asia

## Abstract

Coxiellosis, a zoonotic bacterial infection caused by *Coxiella burnetii*, affects diverse mammalian hosts and is prevalent worldwide, including in South Asia. This study aimed to investigate the epidemiology of Coxiellosis in South Asia, focusing on distribution, host diversity, prevalence, and associated risk factors at the human–animal–environment interface. Following the preferred reporting items for systematic reviews and meta-analyses (PRISMA) guidelines and a registered protocol, online searches were conducted in Embase, PubMed, Scopus, and Web of Science on August 6, 2023, to retrieve articles from the South Asian countries without restrictions on hosts or timeframe. Two authors independently reviewed, extracted data, and assessed quality based on predefined criteria, which were then evaluated and compiled into a single document and analyzed. The review identified 112 articles published between 1954 and 2023. Among humans, the estimated pooled seroprevalence (EPSP) was 9.2%, and the estimated pooled carrier prevalence (EPCP) was 6.2%. Ruminant herd-level EPSP and EPCP were 77.3% and 74.6%, and at the individual level, were 11.9% and 5.3%, respectively. Seroprevalence was significantly influenced by country, tick infestation, reproductive disorders, age, and body condition of ruminants. Nonruminant mammals, such as dogs (16.8%), horses (6.0%), pigs (3.9%), and rodents (14.8%), were also seropositive. Several avian and reptile species showed EPSP rates of 14.5% and 29.2%, respectively. Bacterial DNA was detected in ticks and soil samples, with EPCP of 1.0% and 3.3%, respectively. We recommend prioritizing One Health surveillance and intervention to prevent infections among humans, livestock, poultry, pets, and wildlife. Special emphasis should be placed on aged and emaciated animals, tick infestations, and animals with reproductive disorders.

## 1. Introduction

Coxiellosis is a zoonotic bacterial infection caused by *Coxiella burnetii*, affecting a wide range of hosts globally, including humans, domesticated and wild animals, birds, reptiles, and arthropods. The prevalence of Coxiellosis has been increasing, leading to spontaneous abortions and potential reproductive failures in both humans and animals, as well as substantial economic losses [1, 2]. The infectivity of *C. burnetii* is remarkably high; a single inhaled organism can cause clinical illness. The bacterium can be excreted in milk, urine, and feces and, during parturition, it may also be present in amniotic fluids and the placenta [3]. Furthermore, infected ticks can carry the bacteria in their feces, posing a potential risk of environmental contamination. *C. burnetii* can also form spore-like structures outside the host, providing resistance to heat and drying. This enables the bacterium to survive in the environment and facilitates its transmission through dust and wind [3].

South Asia is the most densely populated region in the world, comprising eight sovereign countries: Afghanistan, Bangladesh, Bhutan, India, Maldives, Nepal, Pakistan, and Sri Lanka [4]. Agriculture is a vital livelihood for many people in this region, with livestock ownership considered essential for food security and economic stability. However, due to a lack of knowledge, poverty, and inadequate health services, zoonotic diseases associated with livestock are frequently reported in South Asia [5]. Coxiellosis is one such disease that has been reported in most countries within the region, yet it remains neglected and underreported, lacking the attention it deserves as a priority issue [5].

To effectively prevent and manage a disease within a community, it is essential to have a comprehensive understanding of the disease. Although some research on Coxiellosis in animals has been conducted in recent years in this region, most studies consist of cross-sectional serological and molecular investigations [6–8]. These studies have identified several risk factors, including sex, breed, and reproductive disorders. To the best of the authors' knowledge, no systematic review or meta-analysis has comprehensively examined the estimated pooled prevalence and associated risk factors in the context of South Asia. Therefore, through this comprehensive review, we aim to explore the overall burden of Coxiellosis in the region and identify key factors contributing to its spread and persistence.

## 2. Materials and Methods

This review adhered to the preferred reporting items for systematic reviews and meta-analyses (PRISMA) guidelines [9] and followed a protocol registered with the Open Science Framework [10]. The PRISMA checklist, PRISMA-S statement, and PRISMA 2020 abstract checklist can be found in Supporting Information 1, 2, and 3, respectively. Initially, one author (Md Mazharul Islam) conducted online literature searches and initially screened the articles by removing the duplicates. Two authors (Md Mazharul Islam and Pronesh Dutta) then independently reviewed the screened articles for eligibility, extracted data, and assessed the quality of the eligible studies. The extracted data and quality assessment reports were subsequently evaluated together by the same two authors (Md Mazharul Islam and Pronesh Dutta), who compiled them into a single document for further analysis. Finally, data analysis was performed by two authors (Md Mazharul Islam and Mohammad Mahmudul Hassan). Any conflicts or uncertainties that arose during the article screening, data extraction, quality assessments, and data evaluation were resolved through discussion among the authors.

### 2.1. Data Search

**Figure 1:**
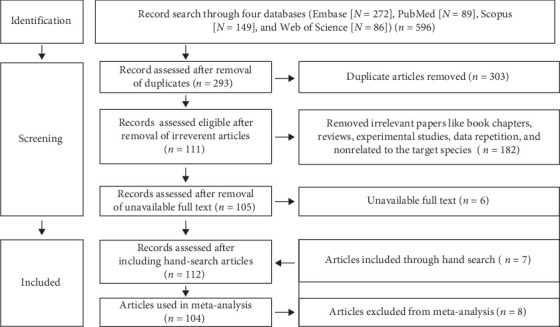
The PRISMA 2020 flow diagram describes the systematic review process for the selection of published articles including the inclusion/exclusion criteria applied in the study.

An optimized systematic search strategy was employed to find published articles on the history of South Asia across four databases: Embase, PubMed, Scopus, and Web of Science (Figure 1). The search was conducted on August 6, 2023, without any timeframe restriction. The keywords used were (Coxiellosis OR “Q fever” OR “Q-fever” OR “*Coxiella burnetii*” OR “*C. burnetii*”) AND (Afghanistan OR Bangladesh OR Bhutan OR India OR Maldives OR Nepal OR Pakistan OR “Sri Lanka”). The search targeted the title, abstract, keywords, and topic of the articles. The search results were downloaded and consolidated into a single EndNote file (EndNote X9, Clarivate Analytics, Philadelphia, PA, USA). Duplicates were then removed using EndNote, and the remaining articles were transferred to the Rayyan system (https://rayyan.qcri.org/), where further screening was conducted based on predefined inclusion and exclusion criteria.

The study's inclusion criteria encompassed cross-sectional studies, longitudinal studies, prevalence studies, risk factor studies, and case reports at any host level. Conversely, reviews, experimental studies, studies unrelated to *C. burnetii*, studies conducted outside South Asia, and non-English language articles were excluded. We obtained the full texts of eligible studies from various sources, including EndNote, PubMed, ScienceDirect, and ResearchGate. If articles were unavailable through these databases, we requested them via the Qatar National Library Interlending and Document Supply Service (https://qnl.qa/en). Additionally, we conducted a manual search of the references cited in the included articles to identify any relevant studies that may have been overlooked during the systematic search process.

### 2.2. Data Extraction

We extracted several variables, including sampling time and location (country and district/state/division), animal-specific data (species, sex, pregnancy, body condition, and history and type of reproductive disorders), ruminant data (herd type, farm management, and farm location), sample type (whole blood, serum, genital sample, aborted material, and milk), season of sampling, and any other relevant study data (Supporting Information 4).

### 2.3. Quality Assessment

We assessed the risk of bias in the included studies using a modified version of the critical appraisal tool for prevalence studies developed by Munn et al. [11]. This tool comprised a checklist of 10 questions to evaluate biases, with response options of “yes,” “no,” “not applicable,” and “not clear”. Each study received a score based on the percentage of “yes” responses out of the total number of “yes,” “no,” and “not clear” responses (excluding “not applicable” responses). Scores ranged from 0 to 100. To categorize the studies according to their risk of bias, we grouped the scores into three categories: low (<40), intermediate (≥40–<70), and high (≥70) [12, 13].

### 2.4. Data Analysis

The aggregated data were recorded in Microsoft Excel spreadsheets (MS Office, 2019), and descriptive analysis, including number, percentage, and 95% confidence interval (CI), was performed using R software (RStudio, Version 4.3.1). To analyze the risk factors, we categorized the animals into different subgroups. Ruminants were divided into large ruminants (cattle, camels, and buffalos) and small ruminants (sheep and goats). Nonruminant mammals included dogs, horses, pigs, and rodents. Additionally, nonmammal samples were classified into birds (pigeons, crows, swallows, chickens, parrots, owlets, and mynahs) and reptiles (snakes, tortoises, and lizards). The distribution of Coxiellosis in South Asia was visualized using ArcMap (Version 10.8). RStudio was used for quantitative and subgroup meta-analyses. The crude prevalence of the disease was divided into categories: estimated pooled seroprevalence (EPSP) for seropositive animals by ELISA and capillary agglutination test and estimated pooled carrier prevalence (EPCP) for animals positive with the pathogen by PCR and animal inoculation test. The crude estimated pooled prevalence of the disease, the 95% CI, and the *p*-value were calculated using a random effect model. The variability and level of heterogeneity among studies were assessed using chi-square analysis (*χ*^2^) with *p*-values, followed by *I*^2^ statistics to determine the degree of heterogeneity, and the Tau-squared (*τ*^2^) test to estimate the variance between the studies. We assigned weights to reflect the amount of information each study contained. The results of the meta-analysis were presented using forest plots. Additionally, funnel plots were generated to evaluate the influence of effect estimates from individual studies against a measure of each study's size or precision.

## 3. Results

### 3.1. Descriptive Statistics

The review identified a total of 112 articles published between 1954 and 2023 (Table 1). Notably, the majority of these articles (*n* = 74, 67.0%, 95% CI: 57.3–75.4) were published after 2010. No articles were found between the years 1955 and 1968, as well as between 1997 and 2008. In terms of geographical distribution, research in this field was predominantly conducted in India (*n* = 71, 63.4%, 95% CI: 53.7–72.1) and Pakistan (*n* = 16, 14.3%, 95% CI: 8.6–22.5). Regarding the methodologies employed in the reviewed studies, it is noteworthy that immunological tests were performed in a significant number of them (*n* = 72, 64.3%, 95% CI: 54.6–72.9), with ELISA emerging as the primary method for antibody detection. Interestingly, prior to 21st century, only one study used the ELISA method for detecting antibodies to the bacteria; instead, the capillary agglutination test was primarily employed for antibody detection. Conversely, the pathogen was successfully detected in 46 studies (41.1%, 95% CI: 32.0–50.8), with PCR being the main method utilized for identifying the bacteria. Additionally, a few articles utilized other methods, such as immunofluorescent antibody (IFA) detection test, modified Ziehl–Neelsen stain, histopathology, immunohistochemistry, and cell culture methods to identify either the bacteria or antibodies. These methods were used as supplementary techniques; hence, we did not incorporate the results from these methods into the meta-analysis.

Out of the 112 selected articles, 71 (66.3%, 95% CI: 56.3–76.9) were considered high quality, while 32 (30.8%, 95% CI: 22.3–40.7) were deemed of intermediate quality, with average quality scores of 86.3 and 54.8, respectively (Table 1, Supporting Information 4). The main factors of the two articles (1.9%, 95% CI: 0.7–8.6) contributing to the low scores included small sample sizes, insufficient statistical analysis, and the absence of subgroup analysis. All meta-analyses revealed heterogeneity in the forest plots; however, ~60% of the articles included in the meta-analysis fell outside the funnel plots (Supporting Information 5). In addition to high- and intermediate-quality articles, data from three articles (2.7%, 95% CI: 0.7–8.2)—one correspondence, one conference article, and one short communication (Supporting Information 4)—were included in the meta-analysis due to their transparent reporting of objectives, methods, and results, which aligned with the eligibility criteria for inclusion. However, these three articles, along with five case reports (4.5%, 95% CI: 1.7–10.6), were excluded from the formal quality assessment.

### 3.2. Distribution of the Disease

**Figure 2:**
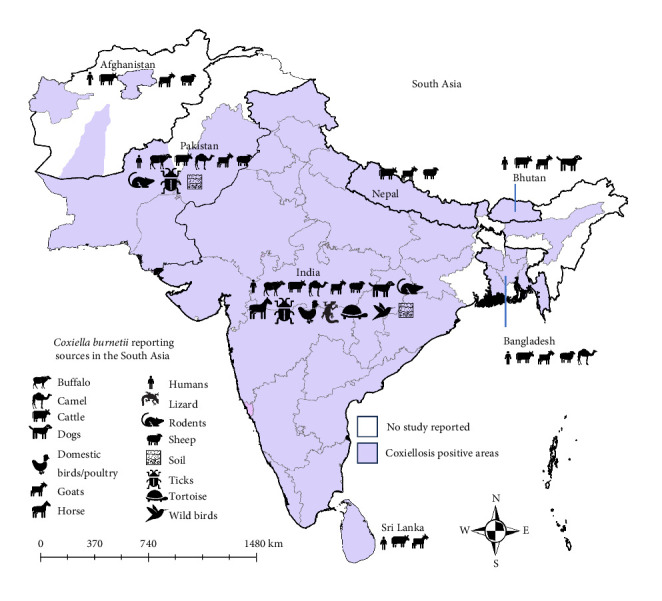
The countries of South Asia where Coxiellosis was reported at the human–animal–environment interface. The icons indicate the presence of Coxiellosis in those sources in the specified countries.

 Coxiellosis has beed observed across various South Asian countries, as shown in Figure 2. In Afghanistan, the disease has been documented in the Herat, Helmand, and Bamyan provinces. In Pakistan, positive cases have been identified solely in the Punjab, Balochistan, and Sindh provinces. In India, Coxiellosis has been reported in nearly every state and two territories, namely Jammu and Chhattisgarh. Furthermore, instances of the disease have also been observed in the Chattogram, Dhaka, Mymensingh, Khulna, and Rajshahi divisions of Bangladesh. Notably, there have been no reported studies in the Maldives.

Human Q fever has been reported in six South Asian countries: Afghanistan, Bangladesh, Bhutan, India, Pakistan, and Sri Lanka. The disease has also been detected in various livestock ruminants, including cattle, camels, sheep, goats, and buffaloes, across all the studied South Asian countries. Evidence of *C. burnetii* infection has been reported in dogs, cats, horses, rodents, birds, reptiles. Additionally, environmental samples, such as ticks and soil, have tested positive for *C. burnetii* using molecular methods, particularly in India and Pakistan.

### 3.3. Q Fever in Humans

**Figure 3:**
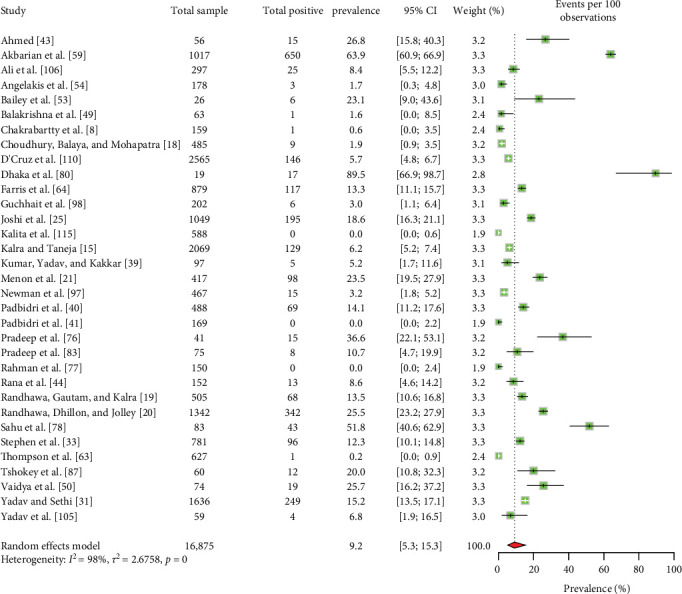
The estimated pooled seroprevalence of Q fever in humans in South Asia (the center dot represents point estimates and green squares represent the weight of each study to the meta-analysis).

**Figure 4:**
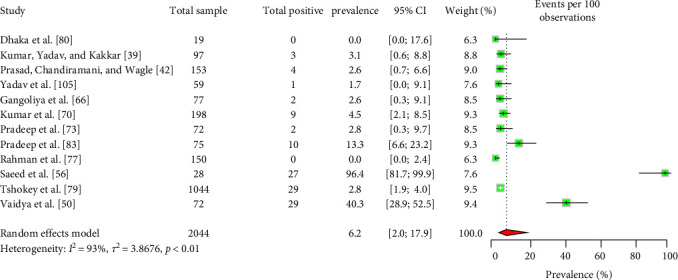
The estimated pooled carrier prevalence of Q fever in humans in South Asia (the center dot represents point estimates and green squares represent the weight of each study to the meta-analysis).

The majority of the human patients with Q fever in South Asia were suffering from fever of unknown origin [8, 43, 49, 53, 54, 109]. Some cases also involved endocarditis and pneumonia [43, 49, 64, 66, 109]. The EPSP of Q fever in humans is 9.2% (95% CI: 5.3–15.3) (Figure 3). Importantly, there is no significant variation in the EPSP of Q fever among different countries, sexes, or age groups (Table 2). Some studies [43, 59] suggested that factors, such as occupation, residence, living status, contact with animals, and consumption of raw milk may influence Q fever seroprevalence; however, the data were insufficient for conducting a meta-analysis. The human EPCP of the disease is 6.2% (95% CI: 2.0–17.9) (Figure 4). Unfortunately, the available data were inadequate for performing a subgroup analysis to identify related potential risk factors within this region. In addition to serum, human milk tested positive for the bacteria and relevant antibodies [39, 42].

### 3.4. Coxiellosis in Ruminants

#### 3.4.1. Herd Level Prevalence and Associated Risk Factors

**Figure 5:**
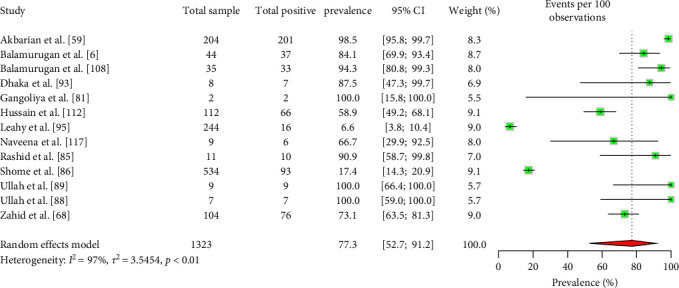
The estimated pooled ruminant herd-level seroprevalence of *C. burnetii* in South Asia (the center dot represents point estimates and green squares represent the weight of each study to the meta-analysis).

Approximately 77.3% (95% CI: 52.7–91.2) of ruminant herds carry seropositive animals (Figure 5). However, there are no significant differences in herd-level seroprevalence based on ruminant type, species, or countries of origin (Table 3). Furthermore, according to the available data, 74.6% (95% CI: 14.6–98.0) of the ruminant herds contain animals positive with Coxiellosis (Supporting Information 6).

#### 3.4.2. Ruminant Level Seroprevalence and Associated Risk Factors

**Figure 6:**
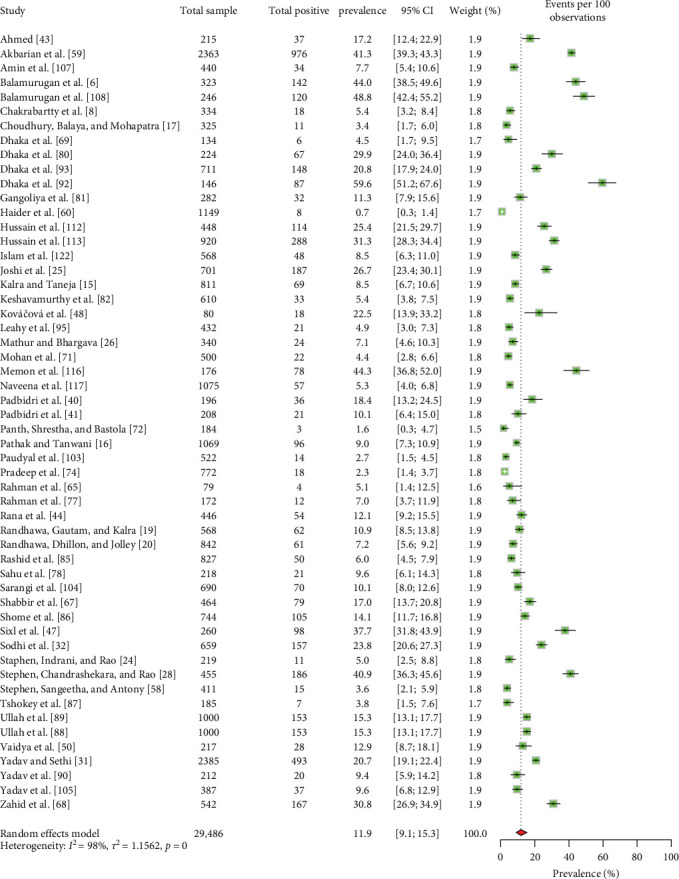
The estimated pooled seroprevalence of *C. burnetii* in livestock ruminants in South Asia (the center dot represents point estimates and gray squares represent the weight of each study to the meta-analysis).

Different types of reproductive disorders, such as abortion, repeat breeding, and retained placenta, were the primary concerns identified in the ruminants tested for Coxiellosis. The seroprevalence of Coxiellosis in livestock ruminants in South Asia was estimated at 11.9% (95% CI: 9.1–15.3) (Figure 6). Stratifying the ruminant-level seroprevalence data by various risk factors revealed (Supporting Information 6) significant differences (*p*  < 0.05) associated with the country of origin, tick infestation, and history of reproductive disorders (Table 4).

Among the general ruminant population (Table 4), Pakistan showed the highest EPSP (18.8%, 95% CI: 9.2–15.4), followed by India (12.0%, 95% CI: 8.8–16.1) and Bangladesh (4.2%, 95% CI: 1.7–9.8). Furthermore, within large ruminants, small ruminants, and goats, Pakistan maintained the highest seroprevalence rates (19.5%, 95% CI: 9.1–16.8; 18.2%, 95% CI: 11.3–28.0; and 19.3%, 95% CI: 11.7–30.0), followed by India (13.3%, 95% CI: 9.2–18.8; 10.7%, 95% CI: 6.8–16.4; and 10.7%, 95% CI: 6.9–16.2) and Bangladesh (2.9%, 95% CI: 0.9–8.9; 4.4%, 95% CI: 1.5–12.3; and 4.0%, 95% CI: 1.2–12.9, respectively) (Tables 5–7). In contrast, for cattle, India reported the highest EPSP (13.8%, 95% CI: 9.5–26.4), followed closely by Pakistan (13.2%, 95% CI: 6.1–26.4), while Bangladesh had the lowest prevalence (2.0%, 95% CI: 0.8–5.1) (Table 7).

Tick infestation was associated with higher seroprevalence rates of Coxiellosis. Infested ruminants showed significantly higher rates (49.8%, 95% CI: 23.3–76.3) compared to their noninfested counterparts (8.4%, 95% CI: 4.1–16.2) (*p*  < 0.01) (Table 4). This trend was also observed among small ruminants, where infested individuals demonstrated substantially higher seroprevalence rates (79.1%, 95% CI: 27.6–97.4) compared to noninfested ones (7.5%, 95% CI: 2.9–17.9) (*p*  < 0.01) (Table 6). Additionally, ruminants with history of reproductive disorders exhibited elevated seroprevalence rates (29.1%, 95% CI: 14.2–50.5) compared to those without such a history (10.4%, 95% CI: 5.1–20.1) (Table 4), a pattern also observed in large ruminants (Table 5).

When analyzing the data by age, we found that seroprevalence did not differ significantly across ages in the overall ruminant population (Table 4). However, in large ruminants, adults exhibited higher seroprevalence (11.09%, 95% CI: 6.8–19.9) compared to younger animals (2.5%, 95% CI: 1.4–4.3) (*p*  < 0.01) (Table 5). This pattern was also observed in cattle, where adults had a seroprevalence of 8.6% (95% CI: 3.5–19.5) compared to 1.9% (95% CI: 0.7–5.4) in young animals (*p*=0.03) (Table 7). In contrast, no significant age-related differences were observed in small ruminants (Table 6). Furthermore, in small ruminants, seroprevalence rates were significantly higher in emaciated animals (44.7%, 95% CI: 38.9–50.5) compared to healthy ones (9.1%, 95% CI: 7.9–10.5) (*p*  < 0.01) (Table 6). Notably, antibodies to the bacteria were also detected in the milk samples of ruminants, with an estimated pooled prevalence of 24.6% (95% CI: 17.8–32.9) (Supporting Information 6).

#### 3.4.3. Ruminant Carrier Prevalence and Associated Risk Factors

**Figure 7:**
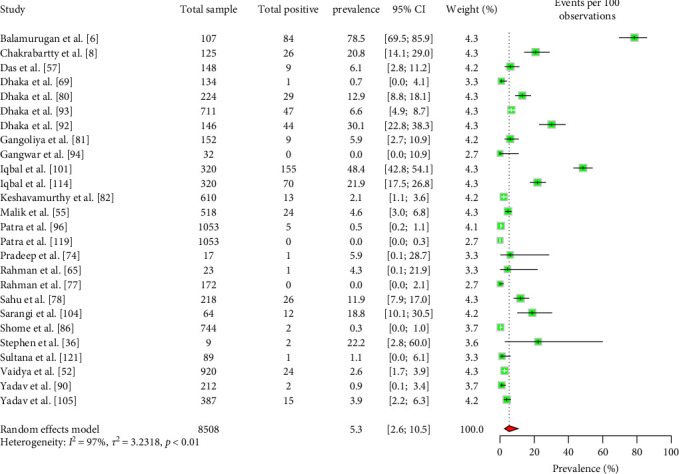
Forest plot of estimated pooled carrier prevalence of *C. burnetii* in ruminants in South Asia (the center dot represents point estimates and gray squares represent the weight of each study to the meta-analysis).

The estimated pooled prevalence of *C. burnetii* in livestock ruminants in South Asia was 5.3% (95% CI: 2.6–10.5) (Figure 7). No significant differences were observed in the EPCP among ruminant types, sample types, species, or the country of origin of the animals (Table 8).

#### 3.4.4. Additional Risk Factors

The reviewed articles examined several potential risk factors for Coxiellosis in livestock ruminants in South Asia. These factors included farm size and purpose, farm location, breed and breeding system, stock replacement, quarantine practice, floor type and space, separate parturition areas, ventilation system, acaricide usage, vaccination practices, carcass disposal, manure management, and seasonal variations. However, the available data were insufficient to conduct a comprehensive meta-analysis to confirm these factors.

A consistent trend observed in the literature is the significant variation in the prevalence of Coxiellosis among herds of different animal breeds [85, 95, 113]. Higher herd prevalence was associated with smaller herd sizes [86]. Additionally, herds with a greater number of lactating animals had a higher prevalence compared to those with fewer lactating animals [88]. Some studies indicated a higher prevalence during the summer season, while others reported a higher prevalence during winter, suggesting the influence of season on the disease prevalence [89, 90, 113]. Furthermore, nonpregnant and nonlactating animals had higher prevalence rates compared to pregnant and lactating animals, respectively [86, 88]. Certain practices, such as introducing new animals to a herd without proper quarantine and having earthen floors, were identified as contributing to an increased seroprevalence of the disease [23, 86, 95].

### 3.5. Coxiellosis in Other Animals and Birds

**Figure 8:**
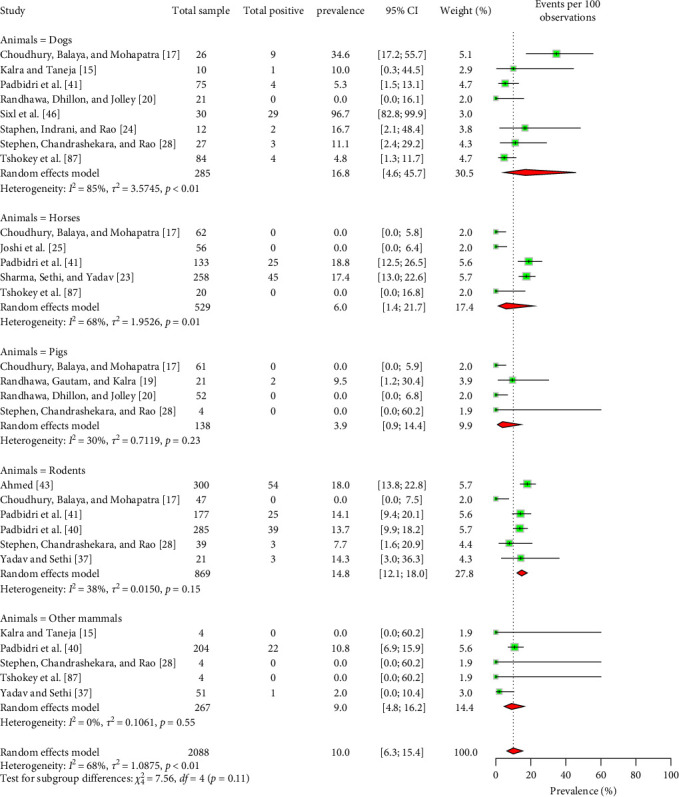
Forest plot of estimated pooled seroprevalence of *C. burnetii* in nonruminant mammals in South Asia (the center dot represents point estimates and gray squares represent the weight of each study to the meta-analysis). Other mammals include cat, monkey, bandicoot, shrew, bandicoot, bat, mongoose, ant eater, jackal, and jungle cat.

Among nonruminant mammals, 16.8% (95% CI: 4.6–45.7) of dogs, 6.0% (95% CI: 1.4–21.7) of horses, 3.9% (95% CI: 0.9–14.4) of pigs, and 14.8% (95% CI: 12.1–18.0) of rodents were found to be seropositive for Coxiellosis (Figure 8). One study reported a seropositivity rate of 10.8% (95% CI: 6.9–15.9) for monkeys [40]. An another study detected Coxiella like bacteria in dogs using molecular technique [118]. Moreover, several avian and reptile species were tested positive for Coxiellosis, with seroprevalence rates of 14.5% (95% CI: 2.3–55.1) and 29.2% (95% CI: 13.2–52.8), respectively (Supporting Information 4 and 6).

### 3.6. *C. burnetii* in Environmental Samples

*C. burnetii* DNA was detected in ticks, with an estimated pooled prevalence (EPCP) rate of 1.0% (95% CI: 0.4–2.4). Additionally, three studies identified the pathogen in soil samples, with a pooled prevalence estimate of 3.3% (95% CI: 1.5–7.1).

## 4. Discussion

The present study provides a comprehensive and updated overview of Coxiellosis at the human–animal–environment interface in South Asia. Previous reports have indicated that this disease is widespread globally, with at least 51 countries documenting cases in domestic ruminants [123]. Our findings reveal evidence of Coxiellosis across most states and regions in the South Asian countries, with the exception of the Maldives. As a country made up of islands, the Maldives has a limited number of livestock [124], which may explain the absence of suitable reservoir animals and the lack of detected Coxiellosis cases. Furthermore, it is evident that Coxiellosis has been largely overlooked in South Asia [125, 126], as our study corroborates. Our review highlights a scarcity of existing research, and the meta-analysis indicates significant heterogeneity, underscoring an information gap pertaining to Coxiellosis in this area.

The first recorded case of Q fever in humans occurred in Lahore, Pakistan, in 1943 [14]. However, due to insufficient details regarding that case, we could not include it in our analysis. The seroprevalence rates of Q fever, particularly in countries such as India—the largest in the region—appear relatively comparable to several countries worldwide [127–129]. Notably, Afghanistan exhibits a higher seroprevalence rate in humans than most other countries [127, 128], which may be linked to the effects of recent wars and conflicts. Such situations have been documented to contribute to the increased prevalence of zoonotic diseases due to intensified interactions between humans and animals, economic challenges, and weakened healthcare systems [130].

The EPSP and EPCP of Coxiellosis at the ruminant herd level in South Asia align with figures observed in many American and European countries. For instance, reported rates include Belgium: 71%, Canada: 67%, Mexico: 82%, France: 84%, and the United States ranging from 38% to 100% [123]. However, research indicates that the herd-level seroprevalence of Coxiellosis in South Asia is higher than that found in neighboring countries, such as Iran (17%) and Turkey (35%) [123]. Additionally, the EPSP in South Asia is lower than that reported in Nigeria, Sudan, Zimbabwe, Canada, and Japan, where seroprevalence rates exceed 20% [123]. The reviewed articles reveal variations in carrier prevalence and seroprevalence of Coxiellosis across different geographic regions, potentially attributed to differences in sampling methodology, climate, topography, and soil composition [85, 86, 88, 95, 113, 116]. Nevertheless, our analyses emphasize that Pakistan reports the highest prevalence of Coxiellosis in domestic ruminants within this region.

Coxiellosis has been identified as a cause of reproductive disorders, including abortion and stillbirth, in ruminants [2], as strongly suggested by one of the reviewed articles in this study [6]. Moreover, it negatively impacts the overall health of animals, leading to physical weakness [88, 89, 107, 114]. Consequently, affected animals may exhibit an emaciated body condition, which is particularly significant in small ruminants, as highlighted in the meta-analysis.

In this region, many animal owners often house animals of different ages and species together within the same compound due to local traditions, poverty, and limited resources. Unfortunately, the implementation of biosafety practices in these situations is inadequate. Essential biosafety measures for such settings include adhering to quarantine protocols, vaccinating animals, consulting with registered veterinarians, maintaining separation between farm animals and extraneous or stray animals, providing an isolation shed for sick animals, and ensuring cleanliness and vector control on the farm. Although testing and culling are suggested methods for the prevention of Coxiellosis at the human–animal interface, such culling practices are not feasible due to the local sociocultural and economic factors of the region. Inadequate biosafety practices can lead to the introduction of diseases into farms via sick, exotic, or newly arrived animals, as well as their spread from one animal to another within a farm or from one farm to another. This transmission can be facilitated by prevailing vectors, such as ticks, or through contact with fomites, aborted materials, and parturition waste [5].

Animal owners incur financial losses attributed to abortion, stillbirth, reduced body condition, decreased milk production, and compromised milk quality [131, 132]. Moreover, the disease poses an occupational hazard, primarily affecting individuals such as veterinarians, milkers, artificial insemination workers, animal attendants, and those who consume unpasteurized or inadequately boiled milk, placing them at a higher risk of infection [133]. Due to a lack of awareness and insufficient veterinary and medical support, cases in humans or animals may go undiagnosed or be recorded as cases of fever of unknown origin. Nonetheless, the disease has been reported in humans in most South Asian countries [125]. Therefore, it is crucial to prioritize prevention and control measures targeting the pathogen.

Our findings reveal that two human studies reported higher seroprevalence rates [59, 80] compared to other studies. Unlike the majority of human studies, which primarily tested for Q fever in patients with persistent fever, myocarditis, or other chronic diseases, these two studies focused on individuals who were animal owners or direct handlers of animals. Although the data were insufficient to conduct a meta-analysis, these studies strongly suggest that Q fever poses a significant occupational health risk. This conclusion aligns with another survey conducted in Afghanistan [56], where 27 out of 28 suspected patients—most of whom were animal handlers—tested positive for the disease.

The study suggests that large ruminant farms with a history of abortion or reproductive disease should be tested for Coxiellosis, as such histories can lead to a high prevalence of the disease [6]. Following an abortion caused by Coxiellosis in livestock, the bacteria can persist in the farm environment for several months [2]. Contamination can occur in various areas, including water alleys, food pens, gutters, and floors [2, 134, 135]. Additionally, arthropods, such as fleas and ticks, act as further sources of infection transmission, as they can carry the pathogen [135]. One potential risk identified in this study is the seropositivity of antibodies to the bacteria in various domestic and wild mammals and nonmammals. Therefore, the effective implementation of biosafety and biosecurity measures, prevention and control of arthropods, regular cleaning of animal sheds, and limiting domestic animal exposure to wildlife are essential for preventing and controlling the disease. The knowledge, attitude, and practice (KAP) of livestock farmers are essential tools in preventing infectious diseases, particularly Coxiellosis at the farm level [136, 137]. Consequently, it is imperative to assess farmers' KAP regarding the risks associated with *C. burnetii* infection and to enhance their understanding and practices for disease prevention and control in South Asia.

The pathogen exhibits a high level of environmental resistance. Given the substantial impact of this disease at the human–animal–ecosystem interface, we recommend adopting a One Health approach as an effective strategy for its prevention and control. This approach entails conducting risk assessments, systematic field investigations, and comprehensive analyses, with targeted interpretations across human, animal, and environmental realms. Embracing a One Health perspective enables early detection, prevention, and response to potential health threats associated with Coxiellosis. The program should incorporate multi-faceted strategies, including public health interventions, veterinary measures, and environmental management practices. By implementing these interventions, the transmission and impact of Coxiellosis within livestock farming communities in South Asia can be reduced. The One Health approach has proven successful in mitigating the spread of infectious diseases in many countries [138]. Its implementation necessitates active involvement from a diverse range of professionals, including medical health practitioners, veterinarians, acarologists, microbiologists, environmental specialists, public health experts, social scientists, local political and religious leaders, school teachers, media representatives, and both local and international policymakers. Together, they form a comprehensive One Health team [139–141]. Thus, we propose implementing a One Health approach to prevent and control Coxiellosis at the human–animal–ecosystem interface.

## 5. Conclusions

Coxiellosis poses a significant public health concern at the human–animal–environment interface in South Asia, with the highest seroprevalence and molecular prevalence rates observed in Pakistan. To address this issue effectively, it is recommended to prioritize diseases affecting cattle and goats, especially those manifesting as emaciation and reproductive disorders. Establishing a comprehensive One Health surveillance system and intervention program is of utmost importance to effectively prevent and control Coxiellosis across the South Asian countries. By integrating efforts across human, animal, and environmental health sectors, proactive measures can be taken to mitigate the spread of this disease and safeguard public health.

## Figures and Tables

**Table 1:** Characteristics of the reviewed studies.

Factors	Number of articles (%) (95% CI)	References
Publication years		
1951–1960^a^	2, 1.8 (0.3–6.9)	[14, 15]
1961−1971^a^	1, 0.9 (0.0–5.6)	[16]
1971–1980	21, 18.8 (12.2–27.5)	[17–37]
1981–1990	10, 8.9 (4.6–16.2)	[38–47]
1991–2000^a^	1, 0.9 (0.0–5.6)	[48]
2001–2010^a^	4, 3.6 (1.2–9.4)	[49–52]
2011–2020	46, 41.1 (32.0–50.8)	[7, 53–97]
2021–2023	27, 24.1 (16.7–33.3)	[6, 8, 13, 98–122]
Coxiellosis tests methods		
Immunologic	72, 64.3 (54.6–72.9)	[6–8, 14, 15, 17–21, 25, 26, 30, 31, 33, 38–44, 47–50, 52–54, 58–60, 63–65, 67–69, 71, 72, 74–78, 80–89, 92, 93, 95, 97–99, 103–108, 110, 112, 115–117]
Molecular	46, 41.1 (32.0–50.8)	[6–8, 50, 52, 55–57, 61, 62, 65, 67, 69, 70, 73, 74, 77–84, 86, 88, 90, 92–94, 96, 99–102, 104, 105, 108, 111, 112, 114, 118–121]
Others	7, 6.3 (2.8–12.9)	[14, 35, 36, 39–42]
Sampling sources/hosts		
Humans	44, 39.3 (30.3–49.0)	[8, 14, 15, 18–21, 25, 31, 33, 39–44, 49–51, 53, 54, 56, 59, 61, 63, 64, 66, 70, 73, 76–80, 83, 87, 91, 97, 98, 105, 106, 109, 110, 115]
Sheep	31, 27.7 (19.8–37.1)	[15–17, 20, 24, 25, 28, 31, 36, 41, 43, 44, 52, 55, 58, 59, 65, 67, 68, 74, 81, 87–89, 95, 101, 103, 107, 116, 121, 122]
Goats	45, 40.2 (31.2–49.88)	[7, 8, 15–17, 19, 20, 24, 25, 28, 31, 36, 41, 43, 44, 47, 48, 52, 55, 58–60, 65, 67, 68, 71, 74, 77, 78, 81, 84, 87–89, 94–96, 101, 103, 105, 107, 116, 119, 121, 122]
Cattle	46, 41.1 (32.0–50.8)	[6–8, 15–17, 19, 20, 24, 25, 28, 31, 32, 38, 40, 41, 43, 44, 47, 48, 52, 55, 57, 59, 60, 65, 69, 72, 74, 75, 77, 80, 82, 85–87, 92, 93, 103–105, 108, 112, 114, 117, 122]
Buffalo	27, 24.1 (16.7–33.3)	[15–17, 19, 20, 24, 28, 31, 32, 36, 40, 41, 43, 44, 52, 55, 57, 74, 75, 82, 85, 86, 90, 93, 112, 114, 117]
Camels	6, 5.4 (2.2–11.7)	[17, 25, 26, 55, 113, 122]
Yak	1, 0.9 (0.0–5.6)	[87]
Cats	2, 1.8 (0.3–6.9)	[37, 87]
Dogs	10, 8.9 (4.6–16.2)	[15, 17, 20, 24, 28, 36, 41, 46, 87, 118]
Horses	5, 4.5 (1.7–10.6)	[17, 23, 25, 41, 87]
Donkey	1, 0.9 (0.0–5.6)	[17]
Bandicoots	3, 2.7 (0.7–8.2)	[28, 36, 37]
Mongoose	1, 0.9 (0.0–5.6)	[37]
Ant eater	1, 0.9 (0.0–5.6)	[37]
Jackal	1, 0.9 (0.0–5.6)	[37]
Rodents	8. 7.1 (3.4–14.0)	[17, 28, 36, 37, 40, 41, 43, 111]
Pigs	5, 4.5 (1.7–10.6)	[17, 19, 20, 28, 57]
Bats	2, 1.8 (0.3–6.9)	[36, 37]
Reptiles	3, 2.7 (0.7–8.2)	[29, 30, 37]
Birds	14, 12.5 (7.3–20.4)	[17, 22, 24, 34, 37, 40, 41, 45, 77, 96, 102, 118–120]
Fishes	1, 0.9 (0.0–5.6)	[30, 37]
Ticks	16, 14.3 (8.6–22.5)	[7, 8, 27, 35, 40, 41, 77, 80, 88, 92, 96, 99, 102, 118–120]
Soil	3, 2.7 (0.7–8.2)	[62, 67, 80]
Studied countries		
Afghanistan	7, 6.3 (2.8–12.9)	[51, 53, 56, 59, 64, 91, 97, 111]
Bangladesh	7, 6.3 (2.8–12.9)	[7, 8, 60, 65, 77, 121, 122]
Bhutan	3, 2.7 (0.7–8.2)	[63, 79, 87]
India	71, 64.0 (54.2–72.7)	[6, 14–42, 44, 49, 50, 52, 55, 57, 58, 61, 66, 69–71, 73–76, 78, 80–84, 86, 90, 92–96, 98, 102, 104, 105, 108–110, 115, 117–120]
Nepal	2, 1.8 (0.3–6.9)	[72, 103]
Pakistan	16, 14.3 (8.6–22.5)	[43, 62, 67, 68, 85, 88, 89, 99–101, 106, 107, 112–114, 116]
Sri Lanka	5, 4.5 (1.7–10.6)	[45–48, 54]
Quality assessment		
High	71, 68.3 (58.3–76.9)	[6, 8, 15, 18–25, 30–37, 40–43, 48, 50, 53, 56, 59, 60, 62, 64, 65, 67, 68, 74, 76, 78–80, 82, 83, 85, 86, 88, 89, 93, 95–102, 104–108, 110–120]
Intermediate	32, 30.8 (22.3–40.7)	[16, 17, 26–29, 38, 39, 44–47, 49, 51, 52, 54, 55, 57, 58, 66, 69–73, 77, 81, 87, 90, 92, 94, 103, 121]
Low	2, 1.9 (0.3–7.5)	[7, 75]

^a^There was not study published between 1955–1968 and 1997–2008.

**Table 2:** Risk factors associated with Q fever in humans based on seroprevalence.

Sl no.	Factors		Number of articles studied	Estimated pooled seroprevalence, 95% CI	Heterogeneity, *I*^2^ (%)	*p* value
1	Country	Afghanistan	4	18.7 (4.3–54.7)	99	0.48
		India	22	10.9 (6.0–18.7)		

2	Sex	Female	7	11.6 (6.9–18.8)	96	0.98
		Male	5	11.5 (5.2–23.4)		

3	Age	Young	4	6.9 (4.4–19.7)	98	0.51
		Adult	5	9.6 (4.4–19.7)		

*Note:* For detail of this table, please refer to Supporting Information 6.

**Table 3:** Risk factors associated with Q fever in ruminant herds based on seroprevalence.

Sl no.	Factors		Number of articles studied	Estimated pooled seroprevalence, 95% CI	Heterogeneity, *I*^2^ (%)	*p* value
1	Ruminant type	Large ruminant	7	73.5 (45.9–90.0)	96	0.89
		Small ruminant	5	69.9 (20.9–95.4)		

2	Species	Cattle	4	85.3 (70.6–93.3)	36	0.11
		Goat	3	70.9 (58.0–81.1)		

3	Country	India	7	62.2 (25.2–89.0)	96	0.43
		Pakistan	5	77.5 (58.0–89.6)		

*Note:* For detail of this table, please refer to Supporting Information 6.

**Table 4:** Estimated pooled seroprevalence of Coxiellosis in livestock ruminants based on different risk factors in South Asia.

Sl no.	Factor	Conditions	Number of articles studied	Estimated pooled prevalence, 95% CI	Heterogeneity, *I*^2^ (%)	*p* value
1	Ruminant type	Large ruminant	42	11.2 (7.8–15.7)	98	0.84
		Small ruminant	34	11.7 (8.4–16.2)		

2	Species	Buffalo	22	11.2 (7.6–16.1)	96	0.92
		Camel	5	14.3 (7.0–26.9)		
		Cattle	39	10.6 (7.2–15.3)		
		Goats	32	11.9 (8.3–16.7)		
		Sheep	27	13.0 (9.7–18.8)		

3	Country	Bangladesh	5	4.2 (1.7–9.8)	98	<0.01
		India	33	12.0 (8.8–16.1)		
		Pakistan	10	18.8 (9.2–15.4)		

4	Age	Adult	16	13.3 (8.8–19.6)	96	0.09
		Young	8	5.3 (1.9–14.0)		

5	Sex	Female	22	12.4 (7.7–19.3)	98	0.17
		Male	9	5.9 (2.2–15.0)		

6	Breed	Cross	6	6.2 (3.3–11.4)	90	0.41
		Local	7	4.2 (2.1–8.1)		

7	Pregnancy	Nonpregnant	3	18.4 (13.0–25.4)	83	0.86
		Pregnant	3	17.1 (7.8–33.6)		

8	Parity	Multiparous	3	26.1 (14.0–43.3)	89	0.97
		Nulliparous	3	27.0 (13.6–46.7)		
		Primiparous	3	24.9 (18.6–32.6)		

9	Body condition	Emaciated	6	12.0 (1.6–53.5)	97	0.64
		Healthy	6	7.3 (2.9–17.2)		

10	Grazing type	Intensive	8	21.7 (9.2–43.2)	97	0.32
		Semi-intensive	8	12.2 (5.3–25.6)		

11	Biosafety (contact with other species)	Does not have	5	9.8 (4.2–21.0)	96	0.18
		Have	5	19.9 (9.7–36.5)		

12	Tick infestation	Present	8	49.8 (23.3–76.3)	98	<0.01
		Absent	7	8.4 (4.1–16.2)		

13	Season	Summar	4	15.6 (7.8–28.7)	95	0.32
		Winter	4	21.6 (19.7–23.6)		

14	History of reproductive disorder	Present	10	29.1 (14.2–50.5)	98	0.04
		Absent	6	10.4 (5.1–20.1)		

15	Type of reproductive disorder	Abortion	8	29.2 (12.9–53.3)	97	0.58
		Others^a^	5	18.6 (3.5–59.2)		

16	History of abortion	Present	6	30.9 (12.5–58.4)	98	0.24
		Absent	5	14.7 (5.3–34.5)		

*Note*: For detail of this table, please refer to Supporting Information 6.

^a^Retained placenta and still birth.

**Table 5:** Estimated pooled seroprevalence of Coxiellosis in large ruminants based on different risk factors in South Asia.

Sl no.	Factor	Conditions	Number of articles studied	Estimated pooled prevalence, 95% CI	Heterogeneity, *I*^2^ (%)	*p* value
1	Country	Bangladesh	4	2.9 (0.9–8.9)	97	0.02
		India	28	13.3 (9.2–18.8)		
		Pakistan	4	19.5 (9.1–16.8)		

2	Age	Adult	12	11.9 (6.8–19.9)	97	<0.01
		Young	5	2.5 (1.4–4.3)		

3	Sex	Female	14	13.1 (7.7–21.2)	97	0.16
		Male	4	3.9 (0.7–18.4)		

4	Breed	Cross	6	6.0 (3.0–11.5)	90	0.40
		Local	6	4.0 (2.1–7.6)		

5	Grazing type	Intensive	6	9.0 (3.3–22.2)	97	0.48
		Semi-intensive	3	14.3 (5.8–30.9)		

6	Biosafety (contact with other species)	Have	3	14.8 (6.0–32.4)	97	0.94
		Does not have	3	14.1 (4.3–37.6)		

7	Tick infestation	Infested	5	32.6 (15.0–57.1)	98	0.06
		Not infested	4	8.0 (2.0–37.4)		

8	History of reproductive disorder	Yes	6	33.5 (18.0–53.5)	98	0.05
		No	4	9.6 (2.8–28.7)		

9	Type of reproductive disorder	Abortion	5	18.9 (5.3–49.3)	97	0.41
		Others^a^	3	6.6 (0.6–44.2)		

*Note*: For detail of this table, please refer to Supporting Information 6.

^a^Retained placenta and still birth.

**Table 6:** Estimated pooled seroprevalence of Coxiellosis in small ruminants based on different risk factors in South Asia.

Sl no.	Conditions	Risk factors	Number of articles studied	Estimated pooled prevalence, 95% CI	Heterogeneity, *I*^2^ (%)	*p* value
1	Country	Bangladesh	4	4.4 (1.5–12.3)	97	0.03
		India	18	10.7 (6.8–16.4)		
		Pakistan	7	18.2 (11.3–28.0)		

2	Age	Adult	4	20.5 (7.8–43.8)	97	0.66
		Young	4	14.4 (3.6–43.5)		

3	Sex	Female	8	20.2 (11.1–33.8)	98	0.42
		Male	5	15.7 (12.6–19.4)		

4	Body condition	Healthy	3	9.1 (7.9–10.5)	98	<0.01
		Emaciated	3	44.7 (38.9–50.5)		

5	Grazing type	Intensive	3	14.1 (1.9–58.0)	97	0.58
		Semi-intensive	4	25.3 (7.7–57.9)		

6	Tick infestation	Infested	3	79.1 (27.6–97.4)	98	<0.01
		Not infested	3	7.5 (2.9–17.9)		

7	Type of reproductive disorders	Abortion	4	33.6 (9.0–72.0)	95	0.86
		Others^a^	3	28.2 (3.9–79.4)		

8	History of abortion	Present	3	45.9 (12.5–83.5)	98	0.40
		Absent	3	23.0 (5.3–61.5)		

*Note*: For detail of this table, please refer to Supporting Information 6.

^a^Retained placenta and still birth

**Table 7:** Estimated pooled seroprevalence of Coxiellosis in different ruminant species based on different risk factors in South Asia.

Sl no.	Conditions	Risk factors	Number of articles studied	Estimated pooled prevalence, 95% CI	Heterogeneity, *I*^2^ (%)	*p* value
1	Country_Cattle	Bangladesh	4	2.0 (0.8–5.1)	96	<0.01
		India	27	13.8 (9.5–26.4)		
		Pakistan	3	13.2 (6.1–26.4)		

2	Country_Sheep	India	15	11.6 (6.4–20.2)	93	0.23
		Pakistan	7	17.9 (11.7–26.4)		

3	Country_Goat	Bangladesh	4	4.0 (1.2–12.9)	96	0.03
		India	18	10.7 (6.9–16.2)		
		Pakistan	7	19.3 (11.7–30.0)		

4	Age_Cattle	Adult	8	8.6 (3.5–19.5)	96	0.03
		Young	3	1.9 (0.7–5.4)		

5	Breed_Cattle	Cross	4	3.4 (1.0–10.6)	*72*	0.58
		Exotic	4	4.8 (2.9–8.1)		

*Note:* For details of this table, please refer to Supporting Information 6.

**Table 8:** Estimated pooled carrier prevalence of Coxiellosis in livestock ruminants based on different risk factors in South Asia.

Sl no.	Factor	Conditions	Number of articles studied	Estimated pooled prevalence, 95% CI	Heterogeneity, *I*^2^ (%)	*p* value
1	Ruminant type	Large ruminant	17	8.6 (3.9–17.8)	96	0.25
		Small ruminant	13	4.1 (1.4–10.9)		

2	Sample type	Whole blood/plasma/serum	14	5.0 (1.5–15.0)	96	0.61
		Genital	8	4.8 (2.0–11.0)		
		Milk	6	7.6 (4.3–13.0)		

3	Species	Buffalo	8	6.4 (2.8–14.0)	94	0.38
		Cattle	15	12.2 (5.4–25.3)		
		Goats	13	4.3 (1.4–12.8)		
		Sheep	8	11.3 (4.6–25.4)		

4	Country	India	20	4.6 (2.1–10.0)	96	0.72
		Bangladesh	4	3.2 (0.5–18.9)		

*Note:* For details of this table, please refer to Supporting Information 6.

## Data Availability

All data were derived from publicly available sources and are included in the online supporting information.

## Supporting Information

Supporting Information 1 The PRISMA 2020 checklist describes the checklist item, and location where the item was reported.

Supporting Information 2 The PRISMA S checklist describes the extension to PRISMA statement for literature search reporting.

Supporting Information 3 The PRISMA 2020 for Abstract checklist.

Supporting Information 4 Extracted data from the selected articles.

Supporting Information 5 Funnel plots depict the seroprevalence and carrier prevalence of Coxiellosis in humans and animals of the South Asian countries.

Supporting Information 6 Forest plots of meta-analysis depict the seroprevalence and carrier prevalence of Coxiellosis in humans and animals of the South Asian countries.
