# Supplementary material for: Prevalence and Risk Factors of Coxiellosis at the Human–Animal–Environment Interface in the South Asian Countries: A Systematic Review and Meta-Analysis
Source: Transbound Emerg Dis. 2025 Jan 31;2025:2890693. doi: 10.1155/tbed/2890693 (PMC12016896; doi:10.1155/tbed/2890693)
Supplement: Supporting Information 4 — Extracted data from the selected articles. [file 2890693.f4.docx]

Prevalence of Coxiellosis in livestock ruminants in South Asian countries: a systematic review and meta-analysis

# Supplementary table 2: Extracted data from the selected articles

| **#** | **Author and country of study and study time** | **Antibody in serum or milk** | **Pathogen or nucleic acid detection** | **Other important findings, Quality grade and marks, and remarks** |
| --- | --- | --- | --- | --- |
| 1 | Ahmed, 1987 [1]  Pakistan (Sindh and Baluchistan) | Method: CFT in serum  Humans: 56/15  Ruminants: 215/37  LR: 90/23; SR: 125/14  Abattoir: 215/37  Buffalo: 55/19  Cattle: 35/4  Sheep: 60/11  Goats: 65/3  Rodents: 300/54  *Ratttus rattus:* 45/14  *Rattus norvegicus*: 30/16  *Tatera indica*: 75/4  *Meriones hurrianae*: 70/16  *Gerbillus nanus*: 30/4  *Nesokia indica*: 20/0  *Bandicota bengalensis*: 10/0  *Milardia meltada*: 20/0 |  | The human samples were patients suffering from enteric fever, typhoid fever, and pneumonia.  High (70%) |
| 2 | Aich et al., 1981 [2]  India (West Bengal)  1975-76 | Method: CAT in milk  Ruminant milk: 131/21  LR milk: 131/12  Cattle milk: 131/12 |  | Intermediate (50%) |
| 3 | Akbarian et al., 2015[3]  Afghanistan (Herath province) December 2012 to January 2013 | Method: ELISA (IgG) in serum  Humans (Animal owners): 1017/650  Herd: 204/201  Ruminants: 2363/976  Sex: F: 2363/976  LR: 344/18  Sex: F: 344/18  SR: 2019/958  Sex: 2019/958  Sheep: 1143/496  Sex: F: 1143/496  Goats: 876/462  Sex: F: 876/462  Cattle: 344/18  Sex: F: 344/18 |  | The sampled humans were households of the animals which were sampled too, considered as animal handlers. In humans, seroprevalence was higher in males than females and it was increasing with age.  In animals, seroprevalence of Q fever was increased with age; High (100%) |
| 4 | Ali et al., 2022 [4]  Pakistan (Punjab) | Method: ELISA (IgG in serum)  Humans (women): 297/25  Urban: 137/17; Rural: 160/8  Living status: High: 97/6; Medium: 83/3; Low: 117/16  Pregnancy: P: 147/17; N: 150/8  HA: No: 158/8; Yes: 139/17  Occupation: Housewife: 66/5; Teacher: 57/3; Student: 67/3; Livestock farmer: 65/12; Business women: 42/2  Contact with animals: Yes: 157/14; No: 140/11  Consumption of raw milk: Yes: 111/6; No: 186/19  Age: ≤20 Y: 65/4; 21-40: 154/6; >40: 77/15 |  | High (90%) |
| 5 | Amin et al., 2022[5]  Pakistan (Punjab province) | Method: ELISA (Indirect) in serum  Ruminants: 440/34  Sex: F: 260/29; M: 40/5  Age: A: 272/34; Y: 28/0  HA: P: 25/3; A: 275/31  TI: P: 52/26; A: 248/8  Grazing: Int: 262/25; SInt: 38/9  CS: P: 45/22; A: 255/12  PS: Healthy: 262/15; Emaciated: 38/19  Residence: Urban: 133/12; Rural: 167/22  Breed: L: 440/34  SR: 440/34  Sex: F: 260/29; Male: 40/5  Age: A: 272/34; Y: 28/0  HA: P: 25/3; A: 275/31  TI: P: 52/26; A: 248/8  Grazing: Int: 262/25; SInt: 38/9  CS: P: 44/23; A: 45/22  PS: Healthy: 262/15; Emaciated: 38/19  Residence: Urban: 133/12; Rural: 167/22  Breed: L: 440/34  Sheep: 142/7  Breed: L: 142/7  Goats: 158/27  Breed: L: 158/27 |  | Contact with fomites, TI, and contact with other species of animals, sedentary production system, and emaciated animals had higher Q fever prevalence; High (100%) |
| 6 | Anderson and Kalra, 1954 [6]  India (Bangalore) 1952 | Method: CFT in serum  *Ricketsia burnetii (Coxiella burnetii*) was confirmed in one American physician living in India | Animal inoculation test in Guinea pig confirmed the pathogen as *Coxiella burnetii* | Case report; A previous epidemic outbreak was reported in Pakistan (Lahore) in 1943. Not included in Meta-analysis |
| 7 | Angelakis et al., 2012 [7]  Sri Lanka (Kandy) 2009 | Method: IFA  Human serum: 178/3 |  | The sampled humans were suffering from fever. Intermediate (50%) |
| 8 | Bailey et al., 2011 [8]  Afghanistan (Halmand)  May to October 2008 | Method: IFA  Human serum: 26/6 |  | British soldiers, suffering from undifferentiated febrile illness in Afghanistan; High (70%) |
| 9 | Balakrishnan et al., 2008 [9]  India (Chennai) August 2005 to December 2006 | Method: Microagglutination test in serum  Humans: 63/1 |  | The patients were suffering from endocarditis. Intermediate (50) |
| 10 | Balamurugan et al., 2021[10]  India  (14 states: Andhra Pradesh, Chhattisgarh, Gujarat, Haryana, Himachal Pradesh, Jharkhand, Karnataka, Madhya Pradesh, Maharashtra, Punjab, Sikkim, Tamil Nadu, Telangana, Uttarakhand) | Method: ELISA (Indirect) and CFT in serum  Herd: 44/37  LR herd: 44/37  Cattle herd: 44/37  Ruminants: 323/142  Sex: F: 323/142  Age: A: 323/142  HRD1: P: 323/142  HRD2: Abortion: 117/70; Others: 206/72  LR: 323/142  Sex: F: 323/142  Age: A: 323/142  HRD1: P: 323/142  HRD2: Abortion: 117/70; Others: 206/72  Cattle: 323/142  Sex: F: 323/142  Age: A: 323/142  HRD1: P: 323/142  HRD2: Abortion: 117/70; Others: 206/72 | Method: *Trans* PCR  Sample: Serum  Herd: 44/43  Ruminants: 107/84  LR: 107/84  Cattle: 107/84 | Q fever is associated with Abortion, infertility, and other repro. Disorder; High (80%) |
| 11 | Balamurugan et al., 2022 [11]  India (Haryana, Jharkhand, Sikkim, Madhya Pradesh, Chhattisgarh, Gujarat, Uttarakhand states; Andhra Pradesh, Karnataka, Tamil Nadu, Telangana, and Maharashtra) April 2015 to March 2021 | Method: ELISA in serum  Herd: 35/33  LR herd: 35/33  Cattle herd: 35/33  Ruminant: 246/120  LR: 246/120  Cattle: 246/120  Sex: F: 246/120  HRD1: P: 246/120  Age: A: 246/120 |  | High (70%). As all the tested animal history of reproductive disorder, the animals were considered as adult |
| 12 | Balasubramanian et al., 2022 [12]  India |  | Two human cases  Pathogen was detected by real time PCR | Case report of patients, suffering from endocarditis. Not included in Meta-analysis |
| 13 | Chakrabartty et al., 2016 [13] Bangladesh (Rajshahi and Dhaka divisions) December 2014-December 2015 | Method: indirect ELISA (Ab) in serum  Ruminants: 172/12  Sex: F: 172/12  Age: F: 172/12  HRD1: 172/12  LR: 81/5  Cattle: 81/5  Sex: F: 81/5  HRD1: P: 81/5  Age: A: 81/5  SR: 91/7  Goats: 91/7  Sex: 91/7  HRD1: P: 91/7  Age: A: 91/7 | Method: real time PCR  Sample: Serum  Ruminants: 172/0  LR: 81/0  SR: 91/0  Cattle: 81/0  Goats: 91/0  Ticks: 24/0 | Q fever is more prevalent with aged goat; Low (40%); Not included in Meta-analysis |
| 14 | Chakrabartty et al., 2021 [14] Bangladesh  (Rajshahi and Dhaka divisions) 2018 to 2021 | Method: ELISA (Ab) in serum  Humans: 159/1  Ruminants: 334/18  Sex: F: 334/18  HRD1: 334/18  HRD2: Abortion: 186/12; Others: 148/6  Age: Y: 188/4; A: 146/14  LR: 162/5  Age: 3-5 Y: 94/2; > 5 Y: 68/3  Sex: F: 162/5  Breed: L: 48/2; CB/Ex: 114/3  HRD1: 162/5  HRD2: Abortion: 86/2; Others: 76/3  SR: 172/13  Sex: F: 172/5  Age: ≤2 Y: 94/2; > 2 Y: 78/11  HRD1: 172/13  HRD: Abortion: 100/10; Others: 72/3  Cattle: 162/5  Sex: F: 162/5  Age: 3-5 Y: 94/2; > 5 Y: 68/3  Breed: L: 48/2; Cross/exotic: 114/3  HRD1: 162/5  HRD2: Abortion: 86/2; Others: 76/3  Goats: 172/13  Sex: F: 172/5  Age: ≤2 Y: 94/2; > 2 Y: 78/11  HRD1: 172/5  HRD2: Abortion: 100/10; Others: 72/3  Ruminant milk: 119/26  LR milk: 119/26  Cattle milk: 119/26 | Method: real time PCR  Ruminants: 125/26  LR: 119/26  SR: 6/0  Genital organ (aborted material)  Goats: 6/0  Milk  Cattle: 119/26  Ticks: 126/1 | The humans were suffering from pyrexia of unknown origin. Additional test: Cell culture: 125/0; High (90) |
| 15 | Chaudhary et al., 2018 [15]  India (Gujrat) | Method: indirect ELISA (Ab) in Milk  Ruminant: 104/30  LR: 104/30  Cattle: 82/23  Buffalo: 22/7 |  | Low (30%); Not included in Meta-analysis |
| 16 | Choudhury et al., 1971 [16]  India (Delhi) | Method: CAT in serum  Ruminants: 325/11  Sex: F: 204/10; M: 121/1  SR: 91/1  Sex: F: 36/1; M: 55/0  LR: 234/10  Sex: F: 168/9; M: 66/1  Camel: 42/5  Cattle: 81/3  Sex: F: 40/2; M: 41/1  Buffalo: 153/7  Sex: F: 128/7; M: 25/0  Sheep: 219/8  Sex: F: 13/0; M: 26/0  Goats: 441/27  Sex: F: 23/1; 29/0  Pigs: 61/0  Sex: Male: 61/0  Horse: 62/0  Sex: Female: 26/0; Male: 36/0  Donkey: 6/0  Sex: Female: 1/0; Male: 5/0  Dogs: 26/9  Rodents: 47/0  Chickens: 51/0 |  | Intermediate (50%) |
| 17 | Choudhury et al., 1972 [17]  India (Delhi) | Method: CFT  Human serum: 485/9 |  | The humans were suffering from pyrexia of unknown origin. High (70%) |
| 18 | D’Cruz et al., 2022 [18]  India (Erode, Nilgiris, Salem, Tiruvannamalai and Vellore Northern  Tamil Nadu) September 2017 to January  2020 | Methods: ELISA in serum  Human: 2565/146  Sex: F: 1540/81; M: 1025/65  Age: 15-25: 364/14; 26-35: 520/24; 36-45: 594/39; 46-55: 459/23; 56-65: 432/27; Above 65: 19/226  Residence: Rural: 1772/89; Urban: 793/57 |  | The human participants were village residents, did not selected as sick patients. High (100%) |
| 19 | Das et al., 2014[19]  India (Utter Pradesh, Goa, Orissa (samples were negative) |  | Method: *Trans* PCR in blood  Ruminants: 148/9  LR: 148/9  Cattle: 88/4  Buffalo: 60/5  Pig: 10/0 | The tested animals had HRD; Intermediate (60%) |
| 20 | Dhaka et al., 2017[20]  India (Kerala) January-May/2017 | Method: indirect ELISA (Ab) in serum  Ruminants: 134/6  Sex: F: 134/6  Age: A: 134/6  HRD1: P: 8/4; A: 126/2  Breed: Cross: 134/6  LR: 134/6  Sex: F: 134/6  Age: A: 134/6  HRD1: P: 8/4; A: 126/2  Cross: 134/6  Cattle: 134/6  Sex: F: 134/6  Age: A: 134/6  HRD1: P: 8/4; A: 126/2  Breed: Cross: 134/6 | Method: *Trans* PCR in serum  Ruminants: 134/1  LR: 134/1  Cattle: 134/1 | 35.55% of animals which had HRD were positive; Intermediate (50%) |
| 21 | Dhaka et al., 2019[21]  India (Utter Pradesh)  August 2015 to February 2016 | Method: indirect ELISA (Ab) in serum and milk  Ruminants: 224/67  Sex: F: 224/67  Age: A: 224/67  HRD1: P: 90/32; A: 134/35  LR: 224/67  Sex: F: 224/67  Age: A: 224/67  HRD1: P: 90/32; A: 134/35  Cattle: 224/67  Sex: F: 224/67  Age: A: 224/67  HRD1: P: 90/32; A: 134/35  Ruminant milk: 217/58  LR milk: 217/58  Cattle milk: 217/58  Human serum: 19/17  Farm workers: 19/17 | Method: *Trans* PCR  Cattle  (Serum): 224/29  Genital sample (Vaginal swab): 224/38  Milk: 217/12  Ticks: 114/0  Feed: 4/0  Environmental sample: 13/0  Soil: 1 positive  Human serum: 19/0  Farm workers: 19/0 | High (90%) |
| 22 | Dhaka et al., 2020a [22]  India (Uttar Pradesh, Rajasthan, Chhattisgarh, Haryana) | Method: indirect ELISA (Ab)  Herd: 8/7  LR herd: 8/7; Cattle herd: 5/5; Buffalo herd: 3/2  Ruminants: 711/148  Sex: F: 711/148  Grazing: SInt: 242/36; Int: 301/60  Quarantine practice: P: 287/42; A: 256/54  LR: 711/148  Sex: F: 711/148  Grazing: SInt: 242/36; Int: 301/60  Quarantine practice: P: 287/42; A: 256/54  Cattle: 543/96  Sex: F: 543/96  Grazing: SInt: 242/36; Int: 301/60  Quarantine practice: P: 287/42; A: 256/54  Buffalo: 168/14 | Method: *Trans* PCR  Whole Blood/serum (Blood)  Herd prevalence: 8/6  Ruminants: 711/47  LR: 711/47  Cattle: 543/46  Buffalo: 168/1 | Q fever is more prevalent in Cattle than buffalo. Prevalence increases with age. Presence of mastitis and other repro. disorder can influence higher prevalence of Q fever; High (90%) |
| 23 | Dhaka et al., 2020b [23] India (Uttar Pradesh, Rajasthan, Haryana) | Methods: ELISA  Ruminant: 146/87  TI: P: 146/87  LR: 146/87  TI: P: 146/87  Cattle: 146/87  TI: P: 146/87 | Methods: Trans PCR  Ruminant: 146/44  TI P: 146/44  LR: 146/44  TI: P: 146/44  Cattle: 146/44  TI: P: 146/44  Ticks: 1648/0 | Short communication. |
| 24 | Essbauer et al., 2022 [24] Afghanistan  2009 to 2012 |  | Methods: real-time PCR  Rodent liver: 199/0 | High (80%) |
| 25 | Farris et al, 2016 [25] Afghanistan 2001-2010 | Method: Immunodot assay  Human Serum: 879/117 |  | US army in Afghanistan; High (80%) |
| 26 | Gangoliya et al., 2016 [26] India (Karnataka) January 2014 and April 2015 |  | Real-time PCR  Human blood: 77/2 | The patients were suffering from atypical pneumonia. Intermediate (60%) |
| 27 | Gangoliya et al., 2019[27]  India (Jammu) | Method: indirect ELISA (Ab)  Herd: 2/2  SR: 2/2  Goat: 2/2  Ruminant: 282/32  Grazing: SInt: 282/32  SR: 282/32  Grazing: SInt: 282/32  Sheep serum: 170/21  Grazing: SInt: 170/21  Goat serum: 112/11  Grazing: SInt: 112/11  Ruminant milk: 148/51  SR milk: 148/51  Sheep milk: 110/42  Goat milk: 38/9 | Method: *Nested* PCR  Genital sample (Vaginal swab)  Ruminants: 152/09  SR: 152/09  Sheep: 123/09  Goats: 29/0 | Intermediate (67%) |
| 28 | Gangwar et al., 2020[28]  India |  | Method: *real time* PCR  Ruminants: 32/0  SR: 32/0  Goats: 32/0  Sex: M: 32/0  Genital organ (Prepetual swab): 32/0 | Intermediate (50%) |
| 29 | Glennie an Bailey, 2010 [29] Afghanistan  01 July 2005 to 30 June 2009 |  | Human: 131/21 | US Army deployed in Afghanistan got Q fever. Method: unclear  Based on the description, the diagnosis was considered as pathogen detection; Intermediate (67%) |
| 30 | Guchhait et al., 2021 [30] India (Karnataka) | Method: IFA  Human blood: 202/6 |  | High (100%) |
| 31 | Haider et al., 2015 [31]  Bangladesh (Chittagong and Rajshahi divisions)  May 2009 – August 2010 | Method: ELISA (Ab)  Ruminants: 1149/8  Sex: F: 713/5; M: 436/3  PS: Healthy: 507/7; Emaciated: 641/1  Breed: L: 1008/5; Cross: 141/3  SR: 529/4  Breed: L: 480/2; Cross: 49/2  LR: 620/4  Breed: L: 528/3; Cross: 92/1  Goats: 529/4  Breed: L: 480/2; Cross: 49/2  Cattle: 620/4  Breed: L: 528/3; Cross: 92/1 |  | Short communication: High (100%) |
| 32 | Hussain et al., 2021 [32] Pakistan (Panjab) |  | Method: Real-time PCR  Ixodid tick pool: 65/25 | The ticks were collected from camels; High (90%) |
| 33 | Hussain et al., 2022a [33]  Pakistan  (Punjab)  October 2020 to January 2021 | Method: indirect ELISA (Ab)  Herd: 112/66  LR herd: 112/66  Ruminants: 448/114  HA: P: 194/91; A: 254/23  TI: P: 179/70; A: 269/44  CS: P: 333/53; A: 115/17  Quarantine practice: P: 152/41; A: 296/73  Pregnancy: P: 162/49; N: 286/65  LR: 448/114  HA: P: 194/91; A: 254/23  TI: P: 179/70; A: 269/44  CS: P: 333/53; A: 115/17  Quarantine practice: P: 152/41; A: 296/73  Pregnancy: P: 162/49; N: 286/65  Cattle: 224/53  Buffalo: 224/61 |  | Acaricide use reduce the Q fever prevalence. Mixed species farming, presence of tick is positively associated and separate parturition area is negatively associated with Q fever prevalence. High (100%) |
| 34 | Hussain, et al., 2022b[34]  Pakistan  (Punjab)  June 2018 to December 2019 | Method: indirect ELISA (Ab)  Ruminants: 920/288  Age: <10 Y:651/163; >10 Y: 269/125  Season: Sum: 619/221; Win: 301/67  HRD1: P: 233/142; A: 687/146  TI: P: 273/140; A: 647/148  Grazing: Int: 251/98; SInt: 320/89; Ex: 349/101  CS: P: 448/129; A: 465/150  PS: Healthy: 437/143; Emaciated: 28/7  LR: 920/288  Age: <10 Y:651/163; >10 Y: 269/125  Season: Sum: 619/221; Win: 301/67  HRD1: P: 233/142; A: 687/146  TI: P: 273/140; A: 647/148  Grazing: Int: 251/98; SInt: 320/89; Ex: 349/101  CS: P: 448/129; A: 465/150  PS: Healthy: 437/143; Emaciated: 28/7  Camel: 920/288  Age: <10 Y:651/163; >10 Y: 269/125  Season: Sum: 619/221; Win: 301/67  Sex: F: 591/231; M: 329/57  HRD1: P: 233/142; A: 687/146  TI: P: 273/140; A: 647/148  Grazing: Int: 251/98; SInt: 320/89; Ex: 349/101  PS: Healthy: 437/143; Emaciated: 28/7 | Method: real time PCR  The molecular work was done on the pooled samples. So, beside demographic statistics, no further analysis was conducted with this study. However, 13 out of 81 pooled samples were positive by real time PCR | Q fever prevalence is different in different geographical areas of Pakistan. The disease is higher prevalent in summer than winter, Barella breed than Marecha breed, aged animal, female than male, tick infested animals, relatively small herd sized farms, Int farming system, animals having repro. disorder, and desert climatic zones. High (100%) |
| 35 | (Iatta et al., 2021) [35] Pakistan (Bahwalpur) October 2018 to November 2019 |  | Method: ET-qPCR  Dog blood: 49/0 | High (90%) |
| 36 | Iqbal et al., 2021  Pakistan[36]  (Punjab) |  | Method: *conventional* PCR  Whole Blood/serum (Blood)  Ruminants: 320/155  Sheep: 160/75  Goats: 160/80 | Occurrences of Coxeilliosis is significantly associated with TI. High (90%)  Repro. disorder and infertility are another associating factor of Q fever prevalence. |
| 37 | Iqbal et al., 2022  Pakistan[37]  (Punjab) |  | Method: *conventional* PCR  Whole Blood/serum (Blood)  Herd prevalence: 160/36  Ruminants: 320/70  Cattle: 160/52  Buffalo: 160/20 | Q fever prevalence is increased with the presence of repro. disorders, such as subclinical mastitis, Abortion, post-Abortion infertility. Higher age animals, species difference, parity, body condition, and TI also increase Q fever prevalence. High (100%) |
| 38 | (Islam et al., 2023) [38] Bangladesh  2017-2018 | Method: ELISA (competitive)  Ruminant: 568/48  LR: 92/8; SR: 476/40  Camel: 36/6  Cattle: 56/2  Sheep: 357/28  Goat: 119/12 |  | Conference article (included in meta-analysis) |
| 39 | Joshi et al., 1979 [39]  India (Rajasthan) | Method: CAT in serum  Humans: 1049/195  Ruminants: 701/187  SR: 354/86; LR: 347/101  Cattle: 338/101  Sheep: 96/38  Goats: 258/48  Horse: 56/0  Camel: 9/0 |  | High (100%) |
| 40 | Kalita et al., 2022 [40] India (Assam)  October 2014-September 2016 | Method: ELISA in serum  Human blood: 588/0 |  | High (90%) |
| 41 | Kalra and Taneja, 1954 [41] India (Madras, Jammu, Amritsar, Jaipur)  1952 | Method: CFT  Human serum: 2269/129  Ruminants: 811/69  SR: 377/36; LR: 434/33  Sheep: 287/33  Buffalo: 143/14  Cattle: 291/19  Goats: 90/3  Dogs: 10/1  Monkeys: 4/0 |  | High (80%) |
| 42 | Keshavamurthy et al., 2019[42]  India (Punjab)  2017-2018 | Method: indirect ELISA (Ab)  Ruminants: 610/33  Sex: M: 21/0; F: 589/33  Age: <1 Y: 52/1; 1-3 Y: 101/2; > 3 Y: 457/30  LR: 610/33  Sex: M: 21/0; F: 589/33  Age: <1 Y: 52/1; 1-3 Y: 101/2; > 3 Y: 457/30  Cattle: 378/24  Breed: Cross: 322/21; L: 56/3  Buffalo: 232/9 | Method: *trans* PCR  Mix of blood and genital sample  Ruminants: 610/13  Cattle: 378/11  Breed: Cross: 378/11; L: 56/2  Buffalo: 232/2 | High (90%) |
| 43 | Kovacova et al., 1996 [43]  Sri Lanka | Method: indirect ELISA (Ab)  Ruminants: 80/18  SR: 40/11; LR: 40/7  Cattle: 40/7  Goats: 40/11 |  | IFA test was also applied but not included in the Meta-analysis; High (90%) |
| 44 | Krishnamoorthy et al., 2021 [44] India (Karnataka and Kerala) October 2017 to September 2018 |  | Method: PCR  Ticks: 240/0 | Tick pools were used; High (80%) |
| 45 | Kumar et al., 1981 [45] India | Method: CAT  Human milk: 97/5 | Method: Mice inoculation  Human milk: 97/3 | Intermediate (60%) |
| 46 | Kumar et al., 2017 [46]India (Karnataka)  June 2013 to December 2013 |  | Method: Real time PCR  Human serum: 198/9 | Intermediate (60%) |
| 47 | Leahy et al., 2020[47]  India (Assam and Odisha)  March to December 2020 | Method: indirect ELISA (Ab)  Herd: 244/16  SR herd: 244/16  Ruminants: 432/21  Quarantine practice: A: 432/21  SR: 432/21  Quarantine practice: A: 432/21  Goats: 411/20  Sheep: 21/1 |  | Herd prevalence is significantly different among geographical locations. Mixed species and smaller herd size increase the herd prevalence. Individual animal level seroprevalence also differ among geographic locations and increased in male animals, introduction of new animals to the farm, and mixed animal farming system; High (80%) |
| 48 | Malik et al., 2013[48]  India (Goa, Maharashtra, Odisha, Rajasthan, and Uttar Pradesh) |  | Method: *trans* PCR  Ruminants milk: 518/24  Cattle milk: 105/2  Buffalo milk: 309/21  Sheep milk: 70/1  Camel milk: 34/0  Goat meat (with HRD): 40/0 | Intermediate (50%) |
| 49 | Mathur and Bhargava, 1979 [49]  India (Rajsthan) | Method: CAT  Ruminants: 340/24  LR: 340/24  Camel: 340/24  Resident: Village: 130/10; City: 210/14 |  | Intermediate (60%) |
| 50 | Menon et al., 1974 [50] India (Kota, Bundi, Barmer, Rajasthan) | Method: CFT  Human serum: 417/98 |  | Males were more positive than females; High (70%) |
| 51 | Mohan et al., 2017[51]  India  (Uttar Pradesh) November 2014 to August 2015 | Method: indirect ELISA (Ab)  Ruminant: 500/22  SR: 500/22  Goat: 500/22 |  | Intermediate (60%) |
| 52 | Memon et al., 2022[52]  Pakistan  (Sindh) | Method: indirect ELISA (Ab)  Ruminants: 176/78  Sex: F: 176/78  Parity: NP: 20/8; PP: 54/18; MP: 98/36  SR: 176/78  Sex: F: 176/78  Parity: NP: 20/8; PP: 54/18; MP: 98/36  Sheep: 32/12  Sex: F: 32/12  Goats: 146/66  Sex: F: 146/66 |  | Seroprevalence can differ among geographic locations and different parity of animals High (80%) |
| 53 | Naveena et al., 2022[53]  India (Maharashtra, Gujarat, Andra, Telangana, Madhya Pradesh, and Punjab)  June 2019 to December 2019 | Method: ELISA (Ab)  Herd: 9/6  LR: 9/6  Cattle herd: 9/6; Buffalo herd: 1/0  Ruminants: 1075/57  Grazing: Int: 1075/57  Breed: Cross: 615/39; Ex: 145/2; L: 244/17  LR: 1075/57  Grazing: Int: 1075/57  Breed: Cross: 615/39; Ex: 145/2; L: 244/17  Cattle: 1004/57  Buffalo: 71/0 |  | High (80%) |
| 54 | Newman et al., 2014 [54]  Afghanistan  (Helmand province)  March 2008 to October 2011 | Method: ELISA  Humans: 467/15  Adult: 467/57 |  | British army deployed in Afghanistan. All samples were considered as adult.  High (80%) |
| 55 | Padbidri et al., 1982 [55]  India (Karnataka) | Method: CFT  Human serum: 488/69  Sex: F: 165/21; M: 323/48  Ruminants: 196/36  LR: 196/36  Cattle: 173/33  Buffalo: 23/3  Monkeys: 67+137/6+16  Rodents: 285/39  *Rattus rattus*: 93/17  *Rattus blanfordi*: 86/6  *Funambulus tristriatus*: 48/2  *Mus booduga*: 18/1  *Suncus murinus*: 18/1  *Rattus rattus*: 14/3  *Mus platythrix*: 5/2  *Hystrix indica*: 2/0  *Petaurista philippensis*: 1/1 | Method: Hemolymph test  Tick: 126/7 | High (90%) |
| 56 | Padbidri et al., 1984 [56]  India (Pune)  1947-1977 | Method: CFT  Human serum: 169/0  Human Sex: M: 117/0; F: 52/0  Ruminants: 208/21  LR: 25/0; SR: 173/21  Cattle: 22/0  Buffalo: 3/0  Goat: 96/11  Sheep: 77/10  Horse: 133/25  Dogs: 75/4  Rodents: 177/25  *Rattus rattus*: 104/16  *Suncus murinus*: 16/2  *Rattus blanfordi*: 17/1  *Mus platythrix*: 13/4  *Rattus rattus*: 4/0  *Mus meltada*: 5/0  *Mus sexicola*: 5/2  *Glunda ellioti*: 1/0  Other small mammals: 12/0 | Method: Hemolymph test  Ticks: 1478/9 | High (80%) |
| 57 | (Panjwani et al., 2015) [57]India (Northern)  2014 |  | Case report of 35 Y old male  PCR positive | Human case report; Not included in Meta-analysis |
| 58 | Panth et al., 2017 [58] Nepal  October 2016 to December 2016 | Method: indirect ELISA (Ab)  Ruminants: 184/3  Age: Y: 189/0; A: 175/3  Sex: F: 184/3  TI: P: 48/3; A: 136/0  PS: healthy: 60/2; emaciated: 121/1  LR: 184/3  Age: Y: 189/0; A: 175/3  Sex: F: 184/3  TI: P: 48/3; A: 136/0  PS: healthy: 60/2; emaciated: 121/1  Cattle: 184/3  Sex: F: 184/3  Age: Y: 189/0; A: 175/3  TI: P: 48/3; A: 136/0  PS: healthy: 60/2; emaciated: 121/1 |  | Intermediate (60%) |
| 59 | Pathak and Tanwani, 1969 [59] India (Madhya Pradesh) | Method: CAT  Ruminant: 1069/96  SR: 534/66; LR: 535/30  Cattle: 375/19  Buffalo: 160/11  Goats: 520/65  Sheep: 14/1 |  | Intermediate (60%) |
| 60 | Patra et al., 2020 [60] India (Mizoram, Manipur,  Meghalaya, Tripura, Nagaland, and Arunachal Pradesh)  June 2018 to July 2019 |  | Method: PCR  Ruminants: 1053/5  SR: 1053/5  Goats: 1053/5  Ticks pool: 30/12 | High (100%) |
| 61 | Patra et al., 2022a [61] India (Mizoram, Manipur, Nagaland, Tripura, Meghalaya, and Arunachal Pradesh)  April 2019 to March 2020 |  | PCR (blood)  Goats: 1053/0  Ticks: 485/10 | High (80%) |
| 62 | (Patra et al., 2022b) [62] India (Mizoram, Manipur, Nagaland, Tripura, Meghalaya, and Arunachal Pradesh)  April 2019 to March 2020 |  | Method: PCR  Dogs: 245/8  Age: Puppies: 45/0, Y: 75/5; A: 125/5  Sex: M: 110/3; F: 135/5  Season: Sum: 80/2; Monsoon: 95/5; Win: 70/1  Tick pool: 478/18 | As it was Coxiella like bacteria, to avoid non-conformity, the data were not included in Meta-analysis. High (100%) |
| 63 | Paudyal et al., 2021 [63]  Nepal | Method: indirect ELISA (Ab)  Ruminants: 522/14  Grazing: Int: 522/14  LR: 162/2  Grazing: Int: 162/2  SR: 360/12  Grazing: Int: 360/12  Sheep: 118/4  Goats: 242/8  Cattle: 162 /2 |  | Intermediate (50%) |
| 64 | Pradeep et al., 2017a [64]  India (Tamil Nadu)  January 2014-December 2015 | Method: indirect ELISA (Ab)  Ruminants: 772/18  SR: 411/15  LR: 394/3  Abattoir: 394/3  Sheep: 216/4  Goats: 195/11  Cattle: 206/1  Buffalo: 188/2 | Method: trans PCR  Whole Blood/serum (Serum)  Ruminants: 17/1  Sheep: 4/0  Goats: 11/0  Cattle: 1/1  Buffalo: 2/0 | Slaughterhouse sample; Intermediate (60%) |
| 65 | Pradeep et al., 2017b [65]  India (Tamil Nadu)  March, 2016 to March, 2017 |  | Method: PCR  Human blood: 72/2  Age: <18 Y: 9/0; >19 Y: 63/2 | High (80%) |
| 66 | Pradeep et al., 2018 [66]  India (Tamil nadu) April 2014-July 2015 | Method: IFA  Human serum: 41/15 |  | High (86%) |
| 67 | Pradeep et al., 2019 [67]  India (Tamil nadu) January 2015-March 2018 | Method: IFA  Human (Animal handlers) serum: 75/8 | Method: PCR  Human (Animal handlers) blood: 75/10 | High (86%) |
| 68 | Prashad et al., 1986 [68]  India (Haryana) | Method: CAT  Human milk: 153/22 | Method: mice inoculation  Human milk: 153/4 | High (75%) |
| 69 | Rahman et al., 2016 [69]  Bangladesh  (Dhaka, Chittagong, Khulna, and Rajshahi divisions)  2007 to 2008 | Method: indirect ELISA (Ab)  Ruminants: 79/4  SR: 51/3; LR: 28/1  Sex: M: 15/1; F: 64/3  Pregnancy: P: 26/2; N: 38/1  Cattle: 28/1  Sheep: 21/2  Goats: 30/1  Ruminant bulk milk: 109/17 | Method: *real time* PCR  Genital organ (Placenta)  Ruminants: 23/1  Cattle: 5/0  Goats: 10/0  Sheep: 8/1 | High (80%) |
| 70 | Rahman et al., 2018 [70]  Bangladesh  (Rajshahi) | Method: indirect ELISA (Ab)  Human serum: 150/0  Ruminant (Cattle and goats): 172/12  Sex: F: 172/12  Age: A: 172/12 | Method: *real time* PCR  Whole Blood/serum (Serum)  Humans: 150/0  Ruminants: 172/0  Ticks: 127/1 | Intermediate (50%) |
| 71 | Rajagunalan et al., 2019[71]  India  (Tamil Nadu) |  | Method: *trans* PCR  Placenta (Genital)  Ruminant/SR/goat: 1/1 | Goat case report; Not included in Meta-analysis |
| 72 | Rana et al., 1987 [72]  India (Delhi) | Method: CAT  Humans (animal handlers) serum: 152/13  Ruminants: 446/54  SR: 368/50  LR: 78/4  Cattle (Rural resident): 48/2  Buffalo (slaughter house): 30/2  Sheep (slaughter house): 278/41  Goats (slaughter house): 90/9 |  | Intermediate (60%) |
| 73 | Randhawa et al., 1972 [73]  India (Punjab) | Method: CAT  Human serum: 505/68  Age: Y: 182/28; A: 323/40  Sex: F: 176/20; M: 329/48  Ruminants: 568/62  SR: 503/40; LR: 65/22  Cattle: 28/10  Buffalo: 37/12  Goats: 503/40  Pigs: 21/2 |  | ≤20 years: Young  >20 years: Adult  High (100%) |
| 74 | Randhawa et al., 1973 [74]  India (Punjab) | Method: CAT  Human serum: 1342/342  Age: Y: 228/12, A: 1114/330  Sex: F: 438/96; 904/246  Ruminants: 842/61  SR: 660/35; LR: 182/26  Cattle: 130/21  Buffalo: 52/5  Sheep: 219/8  Goats: 441/27  Pigs: 52/0  Dogs: 21/0 |  | High (100%) |
| 75 | Rarotra et al., 1978 [75] India (Uttar Pradesh and Rajasthan) | Method: CAT  Poultry: 589/78  Young: 270/15; Adult: 319/63; |  | <6 months: Young  >6 months: Adult  High (80%) |
| 76 | Rashid et al., 2019[76]  Pakistan  (Punjab) | Method: ELISA (Ab)  Herd: 11/10  LR herd: 11/10  Ruminants: 827/50  Sex: F: 774/50; M: 53/0  Age: ≤2 Y: 187/6; >2-5 Y: 423/33; >5 Y: 217/11  HRD1: P: 121/16; A: 758/40  HRD2: Abortion: 52/6; Others: 69/10  HA: P: 52/6; A: 775/44  TI: P: 59/12; A: 768/38  Grazing: SInt: 724/46; Int: 103/4  CS: P: 673/43; A: 154/7  LR: 827/50  Sex: F: 774/50; M: 53/0  Age: ≤2 Y: 187/6; >2-5 Y: 423/33; >5 Y: 217/11  TI: P: 59/12; A: 768/38  Grazing: SInt: 724/46; Int: 103/4  CS: P: 673/43; A: 154/7  HRD1: P: 121/16; A: 758/40  HRD2: Abortion: 52/6; Others: 69/10  HA: P: 52/6; A: 775/44  Cattle: 419/32  Breed: Cross: 38/1; L: 454/24  Buffalo: 408/18 |  | Seroprevalence can differ among different geographical locations, different age group and breeds of animals. The seroprevalence increase in mixed farming, presence of ticks, and reproductive disorders.  High (100%) |
| 77 | Rialch et al., 2022 [77] India (Uttarakhand) |  | Method: PCR  Tick pool: 79/0 | As the study was Coxiella like endosymbionts, the data were not included in meta-analysis. High (86%) |
| 78 | Saeed et al., 2013 [78] Afghanistan (Bamyan)  Oct, 2019 to March, 2020 |  | Method: PCR  Human blood: 28/27 | High (80%) |
| 79 | Sahu et al., 2018 [79]  India (Chhattisgarh and Odisha) | Method: ELISA (Ab)  Humans (Animal handler) serum: 83/43  Ruminants: 218/21  SR: 218/21  Goats: 218/21 | Method: trans PCR  Goats  Whole Blood/serum (Blood): 218/26  Milk: 28/0 | High (80%) |
| 80 | Sarangi et al., 2021[80]  India  2016 to 2018 | Method: ELISA (Ab)  Ruminants: 690/70  Resident: Organized/urban: 690/70  Sex: F: 690/70  Age: A: 690/70  HA: P: 690/70  Grazing: Int: 690/70  Quarantine practice: A: 690/70  LR: 690/70  Resident: Organized/urban: 690/70  F: 690/70  Age: A: 690/70  HA: P: 690/70  Grazing: Int: 690/70  Quarantine practice: A: 690/70  Cattle: 690/70  Resident: Organized/urban: 690/70  F: 690/70  Age: A: 690/70  HA: 690/70  Grazing: Int: 690/70  Quarantine practice: A: 690/70 | Method: *real time* PCR  Cattle Genital sample (aborted material): 64/12 | Longitudinal study; All animals had HA; High (70%) |
| 81 | Shabbir et al., 2015 [81] Pakistan (Lahore) |  | Method: RT-PCR  Soil: 145/7 | High (80%) |
| 82 | Shabbir et al., 2016[82]  Pakistan (Punjab)  2011-2014 | Method: ELISA (Ab)  Ruminants: 464/79  SR: 464/79  Sheep: 184/33  Goats: 280/46 | Method: RT-PCR  Soil: 2425/48 | Farm location is a factor to reduce the disease prevalence. Higher distance from the main road or canal/stream/drain reduces the prevalence of the disease. High (100%) |
| 83 | Sharma et al., 1978 [83]  India (Utter Pradesh) | Method: CAT  Equine: 258/45  Horse: 258/45  Farm: 258/45 |  | High (70%) |
| 84 | Shome et al., 2019[84]  India (Bihar and Assam)  2015-2016 | Method: ELISA (Ab)  Herd: 534/93  LR herd: 534/93  Ruminants: 744/105  Breed: Cross: 524/93; L: 214/11  LR: 744/105  Breed: Cross: 524/93; L: 214/11  Cattle: 719/98  Buffalo: 25/7 | Method: *conventional* PCR  Whole Blood/serum (Blood)  Ruminants: 744/2  LR: 744/2 | Different geographic location has different seroprevalence. Stall feeding, urban farm, higher number of milking animals, introduction of new animals to the herd, earthen floor increases the seroprevalence of the disease. High (100%) |
| 85 | Sixl et al., 1988a [85]  Sri Lanka | Method: Microagglutination test  Ruminants: 260/98  Slaughter house: 260/98  SR: 184/51, LR: 76/47  Cattle: 76/47  Goats: 184/51 |  | Intermediate (50%) |
| 86 | Sixl et al., 1988b [86] Sri Lanka (Colombo) | Method: Microagglutination test  Crow: 40/38 |  | Title level 1:10 to 1: 80 were considered as positive; Intermediate (50%) |
| 87 | Sixl et al., 1988c [87] Sri Lanka (Colombo) | Method: CFT and MAT  Dogs: 30/29 |  | Intermediate (44%) |
| 88 | Sodhi et al., 1980 [88] India (Punjab) | Method: CAT  Ruminant: 659/157  LR: 659/157  Cattle: 198/46  Buffalo: 461/111 |  | High (70%) |
| 89 | Stephen and Rao, 1979a  [89] India (Karnataka) | Method: MAT and CFT  Reptiles: 31/16  Lizard: 1/1 |  | Intermediate (56%) |
| 90 | Stephen and Rao, 1979b [90] India, Karnataka | Method: MAT  Ticks: 80/2 |  | No ticks detail available; Intermediate (56%) |
| 91 | Staphen et al., 1978 [91] India (Karnataka) | Method: CFT  Ruminant: 219/11  LR: 14/2; SR:205/109  Cattle: 10/1  Buffalo: 4/1  Goat: 125/75  Sheep: 80/34  Dogs: 12/2  Chicken: 61/1 |  | High (70%) |
| 92 | Stephen et al., 1979 [92]  India (Karnataka) | Method: MAT and CFT  Ruminants: 455/186  SR: 339/174; LR: 116/12 Cattle: 102/11  Buffalo: 14/1  Sheep: 142/73  Goats: 197/101  Dogs: 27/3  Rodents: 39/3  Pigs: 4/0  Bandicoots: 4/0 |  | High (80%) |
| 93 | (Stephen et al., 1980a) [93] India (Manipal) | Method: MAT and CFT  Human serum: 781/96 |  | High (80%) |
| 94 | (Stephen et al., 1980b) [94] India (Karnataka) | Method: CFT  Chicken: 337/6  L: 260/4; Ex(white leg horn): 77/2 |  | High (70%) |
| 95 | (Stephen et al., 1980c) [95] India (Karnataka) |  | Method: Guineapig inoculation  Tick pool: 20/1 | High (71%) |
| 96 | Stephen et al., 1980d [96] India (Karnataka) |  | Method: Animal inoculation  Ruminant: 9/2  SR: 8/2; LR: 1/0  Sheep: 6/1  Goats: 2/1  Buffalos: 1/0  Dogs: 1/1  Rodents: 16/4  Bandicoots:2/0  Bats: 1/0 | The results were not clear; however, assumed; Intermediate (60%) |
| 97 | Stephen et al., 2014[97]  India (Tamil Nadu)  July 2012 to June 2013 | Method: ELISA (Ab)  Ruminants: 411/15  SR: 411/15  Goats: 195/11  Sheep: 216/4 |  | Intermediate (60%) |
| 98 | Sultana et al., 2022 [98]  Bangladesh  (Dhaka division)  June 2019-January 2020 |  | Method: *conventional* PCR  Visceral organ (liver, lung, spleen, kidney)  Ruminants: 89/1  SR: 89/1  Goats: 75/1  Sheep:14/0 | Slaughter house sample; Other methods include histopathology; Intermediate (50%) |
| 99 | (Thompson et al., 2015) [99] Nepal (Kathmandu)  July of 2008 and August of 2011 | Method: ELISA, IFA  Human serum: 627/1 |  | Correspondence; some data were used in Meta-analysis, but quality evaluation was not done |
| 100 | (Tshokey et al., 2018) [100] Bhutan  October 2014 to June 2015 |  | Method: qPCR  Human blood: 1044/29  Age: <13: 164/0, 13-24: 229/4; 25-36: 263/7; >48: 198/9  Occupation: Farmer: 274/11; Office worker: 269/8; Student: 286/5; Housewife: 149/3; Unemployed: 16/2 | High (100%) |
| 101 | Tshokey et al., 2019 [101]  Bhutan  January to April, 2015 | Method: IFA (Ab)  Human serum: 60/12  Ruminants: 185/7  LR: 10/3; SR: 65/4  Residence: Rural: 164/7; Urban:130/4  Cattle: 120/3  Goats: 45/4  Sheep: 20/0  Dogs: 84/4  Horse: 20/0  Yak: 10//0  Cat: 4/0 |  | Intermediate (60%) |
| 102 | Ullah et al., 2019a[102]  Pakistan (Punjab) January to June, 2016 | Method: indirect ELISA (Ab)  Herd: 9/9  SR herd: 9/9  Goat herd: 4/4  Sheep herd: 9/8  Ruminants: 1000/153  TI: P: 163/98; A: 837/55  CS: P: 833/143; A: 167/10  PS: Healthy: 828/79; Emaciated: 40/16  Season: Sum: 760/102; Win: 240/51  SR: 1000/153  TI: P: 163/98; A: 837/55  CS: P: 833/143; A: 167/10  PS: Healthy: 828/79; Emaciated: 172/74  Season: Sum: 760/102; Win: 240/51  Goats: 500/75  Sheep: 500/78 |  | Mixed species farming, presence of TI, emaciated animals, and wet season increase the seroprevalence of the disease. In addition, the prevalence is different among different geographic region. High (100%) |
| 103 | Ullah et al., 2019b [103]  Pakistan  (Punjab) | Method: indirect ELISA (Ab)  Herd: 7/7  Ruminants: 1000/153  Sex: F: 893/133; M: 107/20  Age: <1 year: 133/22; >1 to 2.5: 186/20; >2.5-4: 241/47; >4: 440/64  HRD1: P: 178/75; A: 822/78  HRD2: Abortion:93/48; Others: 85/27  HA: P: 93/48; A: 907/105  PS: Healthy: 828/79; Emaciated: 70/32  Pregnancy: P: 320/43; N: 573/99  Parity: NP: 198/29; MP: 582/82; PP: 113/22  SR: 1000/153  Sex: F: 893/133; M: 107/20  Age: <1 year: 133/22; >1 to 2.5: 186/20; >2.5-4: 241/47; >4: 440/64  HRD1: P: 178/75; A: 822/78  HRD2: Abortion:93/48; Others: 85/27  HA: P: 93/48; A: 907/105  PS: Healthy: 828/79; Emaciated: 70/32  Pregnancy: P: 320/43; N: 573/99  Parity: NP: 198/29; MP: 582/82; PP: 113/22  Goats: 500/75  Sheep: 500/78 | Pooled seropositive and suspected samples were subjected for real time PCR and found positive, however not used for Meta-analysis  Tick pool: 55/11 | Variation in geographic location causes variation of seroprevalence. Tick infested, lactating, non-pregnant, reproductive disorder, and poor body condition animals had higher seroprevalence of Q fever. High (100%) |
| 104 | (Vaidya et al., 2008) [104]  India (northern) | Method: IFA  Human serum: 74/19  Women: 74/19 | Method: Trans PCR  Humans: 74/16  Women: 74/16  Placenta: 74/9; Genital: 74/9; Fecal: 74/5; Urine: 72/6  Method: qPCR  Humans: 74/16  Women: 74/16  Placenta: 74/11; Genital: 74/9; Fecal: 74/5; Urine: 72/8 | Culture: 76/5; High (88%) |
| 105 | Vaidya et al., 2010 [105]  India (Uttar Pradesh) | Method: IFA and ELISA (Ab)  Ruminants: 217/28  Sex: F: 217/28  Age: A: 217/28  HRD1: P: 217/28  LR: 121/20  Sex: 121/20  Age: A: 121/20  HRD1: P: 121/20  SR: 96/8  Sex: 96/8  Age: A: 96/8  HRD1: P: 96/8  Cattle: 88/14  Sex: F: 88/14  Age: A: 88/14  HRD1: P: 88/14  Buffalo: 33/6  Sex: F: 33/6  Age: A: 33/6  HRD1: P: 33/6  Sheep: 43/5  Sex: F: 43/6  Age: A: 43/6  HRD1: P: 33/6  Goats: 53/3  Sex: F: 53/6  Age: A: 53/6  HRD1: P: 53/6 | Method: *Trans* PCR and *Real time* PCR  Ruminants: 920/24  Genital sample (swab): 217/8  Fecal swab: 217/6  Milk: 162/13  Urine: 107/8    LR: 121/16  Genital sample (swab): 121/6  Fecal swab: 121/4  Milk: 98/9  Urine: 63/4  Cattle: 88/11  Cattle Genital sample (swab): 88/4  Cattle fecal: 88/3  Cattle Milk: 74/7  Cattle Urine: 45/2  Buffalo: 33/5  Buffalo Genital sample (swab): 33/2  Buffalo Fecal: 33/1  Buffalo Milk: 24/2  Buffalo Urine: 18/2  SR: 96/8  Genital sample (swab): 96/2  Fecal swab: 96/2  Milk: 64/4  Urine: 44/4  Sheep: 43/5  Sheep Genital sample (swab): 43/1  Sheep Fecal: 43/2  Sheep Milk: 28/3  Sheep Urine: 17/1  Goats: 53/3  Goats Genital sample (swab): 53/1  Goats Fecal: 53/0  Goats Milk: 36/1  Goats Urine: 27/3 | All sampled animals had reproductive disorder  Additional methods: Cell culture; Intermediate (50%) |
| 106 | (Yadav and Sethi, 1979a [106] India (Uttar Pradesh)  October 1976 – March 1978 | Method: CAT  Snake (including python): 53/13  Tortoise: 16/2  Fish: 15/0  Toad: 66/0  Frog: 7/0 |  | High (90%) |
| 107 | Yadav and Sethi, 1979b[107]  India (Uttar pradesh and Delhi) | Method: CAT  Human serum: 1636/249  Ruminants: 2385/493  Sex: F: 1331/323; M: 1054/170  Age: Y: 327/39; A: 668/158  SR: 1652/329; LR: 733/164  Season: Sum: 304/35; Win: 1039/231  SR  Sex: F: 1119/253; M: 533/76  Age: Y: 304/38; A: 524/125  Sum: 278/32; Win: 813/170  LR  Sex: F: 212/70; M: 521/94  Age: Y: 23/1; A: 144/33  Sum: 26/3; Win: 226/61  Cattle: 402/108  Sex: F: 81/28; M: 321/80  Age: Y: 23/1; A: 144/33  Sum: 26/3; Win: 226/61  Buffalo: 331/56  Sex: F: 131/42; M: 200/14  Sheep: 286/64  Sex: F: 205/51; M: 81/13  Age: Y: 15/2; A: 58/15  Season: Sum: 42/7; Win: 48/11  Goats: 1366/265  Sex: F: 914/202; M: 452/63  Age: Y: 289/36; A: 466/110  Sum: 236/25; Win: 765/159 |  | High (90%) |
| 108 | (Yadav and Sethi, 1980) [108]  India (Uttar Pradesh)  October 1976 – November 1979 | Method: CAT  Mynah (bird): 69/19  Pigeon (bird): 11/1  House sparrow (bird): 12/0  Crow (bird): 13/0  Vulture (bird): 5/0  Owlet (bird): 6/1  Swallow (bird): 200/6  Parrot (bird): 56/13  Heron (bird): 1/0  Duck (bird): 17/0  Guinea fowl (bird): 2/0  Hawks (bird): 4/0  Brahminy kite (bird): 2/0  Rodents: 21/3  Shrew: 21/1  Bandicoot: 1/0  Bats: 14/2  House mouse: 4/0  Mongoose: 5/0  Ant eater: 1/0  Jackal: 2/0  Jungle cat: 3/0  Snake (including python): 35/7  Tortoise: 2/0  Monitor (lizard): 2/0  Eel: 2/0  Unidentified small black bird: 3/0 |  | High (70%) |
| 109 | Yadav et al., 2019 [109]  India  (Uttar Pradesh) | Method: indirect ELISA (Ab)  Ruminants: 212/20  LR: 212/20  Slaughter house: 212/20  Buffalo: 212/20  Sum: 107/9  Win: 105/11 | Method: Conventional PCR and Real time PCR  Buffalo genital sample (uterine): 212/1  Whole Blood/serum (serum): 212/2 | Additional test: Phylogenetic analysis. Winter season is more prevalent than summer season; Intermediate (60%) |
| 110 | Yadav et al., 2021 [110]  India (Utter Pradesh)  February to June 2018 | Method: indirect ELISA (Ab)  Human serum: 59/4  Ruminants: 387/37  Age: A: 387/37  Sex: F: 387/37  LR: 387/37  Age: A: 387/37  Sex: F: 387/37  Cattle: 387/37  Age: A: 387/37  Sex: F: 387/37 | Method: Conventional PCR  Cattle  Whole Blood/serum (serum): 387/15  Genital sample (vaginal swab): 387/7  Milk: 131/9  Human serum: 59/1 | High (75%) |
| 111 | Zahid et al., 2016 [111]  Pakistan  (Punjab) | Method: indirect ELISA (Ab)  Herd: 104/76  SR herd: 104/76  Sheep herd: 52/40  Goat herd: 52/36  Ruminants: 542/167  Sex: F: 482/152; M: 60/13  Age: ≤ 1 Y: 46/16; 2-3 Y: 143/45; >3 Y: 172/63  Parity: NP: 46/16; PP: 121/30; MP: 375/121  HRD: P: 164/122; A: 378/45  HRD: Abortion: 71/55; Others: 93/67  HA: P: 71/55; A: 33/21  TI: P: 95/93; A: 447/74  Grazing: SInt: 94/70; Int: 10/6  SR: 542/167  Sex: F: 482/152; M: 60/13  Age: ≤ 1 Y: 46/16; 2-3 Y: 143/45; >3 Y: 172/63  Parity: NP: 46/16; PP: 121/30; MP: 375/121  HRD: P: 164/122; A: 378/45  HRD: Abortion: 71/55; Others: 93/67  HA: P: 71/55; A: 33/21  TI: P: 95/93; A: 447/74  Grazing: SInt: 94/70; Int: 10/6  Sheep: 271/77  Goats: 271/90 |  | TI is a significant issue to increase the seroprevalence of the disease. High (100%) |
| 112 | Zaibaq-Krill et al., 2019 [112] Afghanistan |  | Human case report. US army member in Afghanistan | Case report; not included in Meta-analysis |

SR = Small ruminants; LR = Large ruminants; Tick Infestation = TI; Present = P, Absent = N, History of Abortion = HA; History of reproductive disorder: HRD; Physiological status = PS; Nulliparous = NP; Primiparous = PP; Multiparous = MP; Semi-intensive = SInt; Intensive = Int; CFT: Complement fixation test; ELISA: Enzyme Linked Immunosorbent Assay; PCR: Polymerase Chain Reaction; MAT: Microagglutination Test; IFA: Immunofluorescent Assay; Adult: A; Young: Y; Contact with other species: CS; Local: L; Years: Y; Summar: Sum; Winter: Win

# References:

[1] Ahmed IP. A serological investigation of Q fever in Pakistan. Journal of the Pakistan Medical Association. 1987;37(4):126-129.

[2] Aich A, Halder S, Khanna P, et al. Lactoserological study for detection of Q fever infection in freshly secreted bovine milk. Indian Journal of Public Health. 1981;24(3):154-156.

[3] Akbarian Z, Ziay G, Schauwers W, et al. Brucellosis and Coxiella burnetii Infection in Householders and Their Animals in Secure Villages in Herat Province, Afghanistan: A Cross-Sectional Study [Article]. PLoS Neglected Tropical Diseases. 2015;9(10).

[4] Ali S, Saeed U, Rizwan M, et al. Serological prevalence of and risk factors for Coxiella burnetti infection in women of Punjab Province, Pakistan. International Journal of Environmental Research and Public Health. 2022;19(8):4576.

[5] Amin F, Ali S, Javid A, et al. Sero-Epidemiology of Coxiella burnetii Infection in Small Ruminants in the Eastern Region of Punjab, Pakistan [Article]. Pathogens. 2022;11(6).

[6] Anderson R, Kalra S. Q fever studies in India: a case of human Q fever. Indian Journal of Medical Research. 1954;42(3):307-14.

[7] Angelakis E, Munasinghe A, Yaddehige I, et al. Detection of rickettsioses and Q fever in Sri Lanka. The American journal of tropical medicine and hygiene. 2012;86(4):711.

[8] Bailey MS, Trinick T, Dunbar J, et al. Undifferentiated febrile illnesses amongst British troops in Helmand, Afghanistan. BMJ Military Health. 2011;157(2):150-155.

[9] Balakrishnan N, Menon T, Fournier P-E, et al. Bartonella quintana and Coxiella burnetii as causes of endocarditis, India. Emerging infectious diseases. 2008;14(7):1168.

[10] Balamurugan V, Alamuri A, Kumar KV, et al. Prevalence of Coxiella burnetii Antibodies in Dairy Cattle Associated with Abortions and Reproductive Disorders [Article]. Proceedings of the National Academy of Sciences India Section B - Biological Sciences. 2021;91(2):353-359.

[11] Balamurugan V, Kumar KV, Alamuri A, et al. Prevalence of Toxoplasma gondii, Leptospira spp., and Coxiella burnetii-associated antibodies in dairy cattle with reproductive disorders. Veterinary World. 2022;15(12):2844.

[12] Balasubramanian R, Fournier PE, Ganesan PS, et al. Q fever endocarditis in India: A report of two cases [Article]. Indian Journal of Medical Microbiology. 2022;40(2):315-316.

[13] Chakrabartty A, Bhattacharjee PA, Sarker R, et al. Prevalence of Coxiella burnetii infection in cattle, Black Bengal goats and ticks in Bangladesh. Bangladesh Journal of Veterinary Medicine. 2016 07/21;14:65-68.

[14] Chakrabartty A, Nahar N, Rahman M, et al. Sero-molecular investigation of Coxiella burnetii infection in domestic ruminants and humans and associated risk factors based on ‘one health’ perspectives in Bangladesh. Journal of Veterinary Medical and One Health Research. 2021 06/30;3, Issue 1.

[15] Chaudhary J, Nayak JB, Brahmbhatt MN, et al. Prevalence of Coxiella burnetii from Raw Milk Samples in and Around Anand. International Journal of Pure and Applied Biosience. 2018 11/20;6(5):447-451.

[16] Choudhury S, Balaya S, Mohapatra LN. Serologic evidence of Coxiella burnetii infection in domestic animals in Delhi and surrounding areas [Article]. The Indian journal of medical research. 1971;59(8):1194-1202.

[17] Choudhury S, Balaya S, Mohapatra L. Presence of complement fixing antibody against Coxiella burnetii in man in Delhi. The Indian Journal of Medical Research. 1972;60(2):178-181.

[18] D’Cruz S, Perumalla SK, Yuvaraj J, et al. Geography and prevalence of rickettsial infections in Northern Tamil Nadu, India: a cross-sectional study. Scientific Reports. 2022;12(1):20798.

[19] Das DP, Malik SVS, Rawool DB, et al. Isolation of Coxiella burnetii from bovines with history of reproductive disorders in India and phylogenetic inference based on the partial sequencing of IS1111 element [Article]. Infection, Genetics and Evolution. 2014;22:67-71.

[20] Dhaka P, Malik SS, Yadav JP, et al. Seroscreening of lactating cattle for Coxiellosis by Trans-PCR and commercial ELISA in Kerala, India. Journal of experimental biology and agricultural sciences. 2017 JUN;5(3):377-383.

[21] Dhaka P, Malik SS, Yadav JP, et al. Seroprevalence and molecular detection of coxiellosis among cattle and their human contacts in an organized dairy farm [Article]. Journal of Infection and Public Health. 2019;12(2):190-194.

[22] Dhaka P, Malik SVS, Yadav JP, et al. Apparent prevalence and risk factors of coxiellosis (Q fever) among dairy herds in India [Article]. PLoS ONE. 2020;15(9 September).

[23] Dhaka P, Malik SVS, Yadav JP, et al. Molecular investigation of the status of ticks on infected cattle for Coxiella burnetii in India. Acta Parasitologica. 2020;65:779-782.

[24] Essbauer S, Baumann K, Schlegel M, et al. Small mammals as reservoir for zoonotic agents in Afghanistan. Military Medicine. 2022;187(1-2):e189-e196.

[25] Farris CM, Pho N, Myers TE, et al. Seroconversions for Coxiella and rickettsial pathogens among US marines deployed to Afghanistan, 2001-2010 [Article]. Emerging Infectious Diseases. 2016;22(8):1491-1493.

[26] Raj Gangoliya S, Kumar S, Imteyaz Alam S, et al. First molecular evidence of Coxiella burnetii in patients with atypical pneumonia, India. Journal of Medical Microbiology. 2016;65(3):255-256.

[27] Gangoliya SR, Kumar S, Alam SI, et al. First molecular and serological evidence of Coxiella burnetti infection among sheep and goats of Jammu province of India. Microb Pathog. 2019 May;130:100-103.

[28] Gangwar C, Kumaresan G, Mishra AK, et al. Molecular detection of important abortion-causing microorganisms in preputial swab of breeding bucks using PCR-based assays. Reprod Domest Anim. 2020 Nov;55(11):1520-1525.

[29] Glennie JS, Bailey MS. UK Role 4 military infectious diseases at Birmingham Heartlands Hospital in 2005-9 [Article]. Journal of the Royal Army Medical Corps. 2010;156(3):162-164.

[30] Guchhait P, Devi DRG, Indumathi VA, et al. Detection of atypical pathogens in community acquired pneumonia by indirect immunofluorescence assay [Article]. Journal of Clinical and Diagnostic Research. 2021;15(2):DC10-DC14.

[31] Haider N, Rahman MS, Khan SU, et al. Serological Evidence of Coxiella burnetii Infection in Cattle and Goats in Bangladesh. Ecohealth. 2015 Jun;12(2):354-8.

[32] Hussain S, Saqib M, Ashfaq K, et al. First molecular evidence of Coxiella burnetii in ticks collected from dromedary Camels in Punjab, Pakistan. Pak Vet J. 2021:1-5.

[33] Hussain S, Hussain A, Aziz MU, et al. First serological evidence of Q fever in large ruminants and its associated risk factors in Punjab, Pakistan [Article]. Scientific Reports. 2022;12(1).

[34] Hussain S, Saqib M, El-Adawy H, et al. Seroprevalence and Molecular Evidence of Coxiella burnetii in Dromedary Camels of Pakistan. Frontires in Veterinary Science. 2022 JUN 16;9.

[35] Iatta R, Sazmand A, Nguyen V-L, et al. Vector-borne pathogens in dogs of different regions of Iran and Pakistan. Parasitology Research. 2021:1-10.

[36] Iqbal MZ, Durrani AZ, Khan JA, et al. Molecular epidemiology of Coxiella Brunetii in small ruminants in Punjab, Pakistan: a novel reporting analytical cross sectional study [Article]. Tropical Animal Health and Production. 2021;53(1).

[37] Iqbal MZ, Durrani AZ, Khan JA, et al. Molecular Identification of Coxiella burnetii, and Incidence and Risk Factors of Coxiellosis in Bovines of Punjab, Pakistan [Article]. Pakistan Journal of Zoology. 2022;54(4):1859-1867.

[38] Islam S, Rahman M, Abedin J, et al. SEROPREVALENCE OF AND RISK FACTORS OF RIFT VALLEY FEVER AND Q FEVER IN DOMESTIC RUMINANTS OF BANGLADESH. International Journal of Infectious Diseases. 2023;130:S147.

[39] Joshi MV, Padbidri VS, Rodrigues FM, et al. Prevalence of Coxiella burnetii infection among humans and domestic animals of Rajasthan State, India [Article]. Journal of Hygiene Epidemiology Microbiology and Immunology. 1979;23(1):67-73.

[40] Kalita D, Deka S, Sharma KR, et al. Seasonal predominance of atypical agents in adult community-acquired pneumonia in India's northeastern region: Is it the time to look again at empirical therapy guidelines? [Article]. Tropical Doctor. 2022;52(2):304-306.

[41] Kalra S, Taneja B. Q fever in India: a serological survey. Indian Journal of Medical Research. 1954;42(3):315-318.

[42] Keshavamurthy R, Singh BB, Kalambhe DG, et al. Prevalence of Coxiella burnetii in cattle and buffalo populations in Punjab, India [Article]. Preventive Veterinary Medicine. 2019;166:16-20.

[43] Kováčová E, Sixl W, Stünzner D, et al. Serological examination of human and animal sera from six countries of three continents for the presence of rickettsial antibodies [Article]. European Journal of Epidemiology. 1996;12(1):85-89.

[44] Krishnamoorthy P, Sudhagar S, Goudar AL, et al. Molecular survey and phylogenetic analysis of tick-borne pathogens in ticks infesting cattle from two South Indian states. Veterinary Parasitology: Regional Studies and Reports. 2021;25:100595.

[45] Kumar A, Yadav M, Kakkar S. Human milk as a source of Q-fever infection in breast-fed babies. 1981.

[46] Kumar S, Gangoliya SR, Alam SI, et al. First genetic evidence of Coxiella burnetii in cases presenting with acute febrile illness, India. Journal of medical microbiology. 2017;66(3):388-390.

[47] Leahy E, Shome R, Deka RP, et al. Risk factors for Brucella spp. and Coxiella burnetii infection among small ruminants in Eastern India [Article]. Infection Ecology and Epidemiology. 2020;10(1).

[48] Malik SVS, Das D, Rawool D, et al. Screening of Foods of Animal Origin for Coxiella burnetii in India. Advances in Animal and Veterinary Sciences. 2013 08/28;1:107-110.

[49] Mathur KN, Bhargava SC. Seroprevalence of Q fever and brucellosis in camels of Jorbeer and Bikaner, Rajasthan State [Article]. Indian Journal of Medical Research. 1979;70(3):391-393.

[50] Menon R, Padbidri V, Joshi M, et al. A preliminary note on the survey of antibodies to certain rickettsial agents in human sera collected from Rajasthan in the year 1971. The Indian journal of medical research. 1974;62(10):1534-1537.

[51] Mohan V, Nair A, Kumar M, et al. Seropositivity of goats for coxiellosis in Bareilly Region of U.P. India [Short Survey]. Advances in Animal and Veterinary Sciences. 2017;5(5):226-228.

[52] Memon A, Kamboh AA, Soomro SA, et al. Sero-Epidemiological Investigation of Abortifacient Bacteria in Goats and Sheep in Three Districts of Sindh Province of Pakistan. Pakistan Journal of Zoology. 2022 01/01.

[53] Naveena T, Sarangi LN, Rana SK, et al. Seroprevalence to common infectious abortifacient and infertility causing agents in the dairy herds of India. Iran J Vet Res. 2022;23(3):189-195.

[54] Newman ENC, Johnstone P, Bridge H, et al. Seroconversion for infectious pathogens among UK military personnel deployed to Afghanistan, 2008–2011 [Article]. Emerging Infectious Diseases. 2014;20(12):2015-2022.

[55] Padbidri V, Bhat H, Rodrigues F, et al. Tick-borne rickettsioses in Karnataka. Indian journal of medical research. 1982;75:507-514.

[56] Padbidri V, Rodrigues J, Shetty P, et al. Tick-borne rickettsioses in Pune district, Maharashtra, India. International journal of zoonoses. 1984;11(1):45-52.

[57] Panjwani A, Shivaprakasha S, Karnad D. Acute Q Fever Pneumonia. The Journal of the Association of Physicians of India. 2015;63(12):83-84.

[58] Panth Y, Shrestha SP, Bastola RC, editors. Demonstration of circulating antibodies of Coxiella burnetii in dairy cattle of Rupandehi district, Nepal2017.

[59] Pathak P, Tanwani S. Serological investigations into Q fever. The Indian Veterinary Journal. 1969;46(7):551-553.

[60] Patra G, Ghosh S, Priyanka, et al. Molecular detection of Coxiella burnetii and Borrelia burgdorferi in ticks infesting goats in North-Eastern states of India. International Journal of Acarology. 2020;46(6):431-438.

[61] Patra G, Polley S, Efimova M, et al. Prevalence and molecular detection of tick borne pathogens in goats and ticks from different parts of North Eastern regions of India. International Journal of Acarology. 2022;48(2):106-113.

[62] Patra G, Ghosh S, Polley S, et al. Molecular detection and genetic characterization of Coxiella-like endosymbionts in dogs and ticks infesting dogs in Northeast India. Experimental and Applied Acarology. 2022;86(4):549-566.

[63] Paudyal N, Poudel S, Pandey D, et al. Sero-detection of Coxiella burnetii infection in cattle, sheep and goats in selected regions of Nepal. Vet Med Sci. 2021 Jul;7(4):1211-1215.

[64] Pradeep J, Stephen S, Pooja P, et al. Coxiellosis in domestic livestock of Puducherry and Tamil Nadu: Detection of Coxiella burnetii DNA by polymerase chain reaction in slaughtered ruminants. Vet World. 2017 Jun;10(6):667-671.

[65] Pradeep J, Stephen S, Ambroise S, et al. Diagnosis of acute Q fever by detection of Coxiella burnetii DNA using real-time PCR, employing a commercial genesig easy kit. Journal of clinical and diagnostic research: JCDR. 2017;11(9):DC10.

[66] Pradeep J, Kumar S, Stephen S, et al. Detection of acute Q fever human cases by indirect immunofluorescence & real-time polymerase chain reaction in a tertiary care hospital in Puducherry. The Indian Journal of Medical Research. 2018;148(4):449.

[67] Pradeep J, Stephen S, Sangeetha B, et al. Application of immunofluorescence assay and nested polymerase chain reaction for query fever diagnosis in animal handlers of Puducherry, South India, and phylogenetic analysis based on IS1111 repetitive gene element. Veterinary World. 2019;12(11):1769.

[68] Prasad B, Chandiramani N, Wagle A. Isolation of Coxiella burnetii from human sources. International journal of zoonoses. 1986;13(2):112-117.

[69] Rahman MA, Alam MM, Islam MA, et al. Serological and Molecular Evidence of Q Fever in Domestic Ruminants in Bangladesh [Article]. Veterinary Medicine International. 2016;2016.

[70] Rahman MS, Chakrabartty A, Sarker RR, et al. Molecular epidemiology of Coxiella burnetii in human, animals and ticks in Bangladesh. African Journal of Microbiology Research. 2018 02/14;12(6):136-140.

[71] Rajagunalan S, Gururaj K, Lakshmikantan U, et al. Detection of the presence of Coxiella burnetii in a case of goat abortion: a first report from India [Article]. Tropical Animal Health and Production. 2019;51(4):983-986.

[72] Rana UV, Sehgal S, Bhatia R, et al. Antibody against Coxiella burnetti in animals and humans in Delhi [Article]. The Journal of communicable diseases. 1987;19(2):152-155.

[73] Randhawa AS, Gautam OP, Kalra DS. Serological prevalence of Q fever in man and animals of Punjab [Article]. The Indian journal of medical research. 1972;60(7):1016-1021.

[74] Randhawa AS, Dhillon SS, Jolley WB. Serologic prevalence of Q fever in the state of Punjab, India [Article]. American journal of epidemiology. 1973;97(2):131-134.

[75] Rarotra J, Yadav M, Sethi M. Sero-epidemiology of Q-fever in poultry. Avian diseases. 1978:167-169.

[76] Rashid I, Saqib M, Ahmad T, et al. Sero-Prevalence and Associated Risk Factors of Q Fever in Cattle and Buffaloes Managed at Institutional Dairy Farms. Pakistan Veterinary Journal. 2019;39(2):221-225.

[77] Rialch A, Sankar M, Silamparasan M, et al. Molecular detection of Coxiella-like endosymbionts in Rhipicephalus microplus from north India. Veterinary Parasitology: Regional Studies and Reports. 2022;36:100803.

[78] Saeed KMI, Ahadi J, Sahak MN, et al. Concurrent Brucellosis and Q fever infection: a case control study in Bamyan Province, Afghanistan. Central Asian Journal of Global Health. 2013;2(2).

[79] Sahu R, Kale SB, Vergis J, et al. Apparent prevalence and risk factors associated with occurrence of Coxiella burnetii infection in goats and humans in Chhattisgarh and Odisha, India [Article]. Comparative Immunology, Microbiology and Infectious Diseases. 2018;60:46-51.

[80] Sarangi LN, Tharani N, Polapally S, et al. Infectious bovine abortions: observations from an organized dairy herd [Article]. Brazilian Journal of Microbiology. 2021;52(1):439-448.

[81] Shabbir MZ, Jamil T, Ali AA, et al. Prevalence and distribution of soil-borne zoonotic pathogens in Lahore district of Pakistan. Frontiers in Microbiology. 2015;6:917.

[82] Shabbir MZ, Akram S, Hassan ZU, et al. Evidence of Coxiella burnetii in Punjab province, Pakistan. Acta Trop. 2016 Nov;163:61-9.

[83] Sharma V, Sethi M, Yadav M. PREVALENCE OF AGGLUTINATING ANTIBODIES TO COXIELLA BURNETII AND BRUCELLA ABORTUS IN SAMPLES OF EQUID SERA FROM UP (INDIA). Equine Veterinary Journal. 1978;10(2):126-128.

[84] Shome R, Deka RP, Milesh L, et al. Coxiella seroprevalence and risk factors in large ruminants in Bihar and Assam, India [Article]. Acta Tropica. 2019;194:41-46.

[85] Sixl W, Withalm H, Stünzner D, et al. Serological examinations of slaughtered animals (cattle and goats) in the slaughter-house of Colombo/Sri Lanka towards antibodies against brucellosis, Q-fever, RMSF-rickettsia-group, listeriosis, echinococcosis and adenoviruses. Geogr Med Suppl. 1988;1:77-80.

[86] Sixl W, Wisidagama E, Stünzner D, et al. Serological examinations of crows in Colombo's slaughter-house Sri Lanka. Geographia medica Supplement= Geographia medica Sonderband. 1988;1:87-88.

[87] Sixl W, Wisidagama E, Stünzner D, et al. Serological examinations of dogs (Canis familiaris) in Colombo/Sri Lanka. Geographia medica Supplement= Geographia medica Sonderband. 1988;1:89-92.

[88] Sodhi S, Joshi D, Sharma D, et al. Seroprevalence of brucellosis and Q fever in dairy animals. Zentralblatt für Veterinärmedizin Reihe B. 1980;27(8):683-685.

[89] Stephen S, Rao KA. Coxiellosis in reptiles of South Kanara district, Karnataka. Indian Journal of Medical Research. 1979;70:937-941.

[90] Stephen S, Achyutha Rao KN. Q fever in South Kanara district: natural occurrence of Coxiella burnetii in the tick (Aponomma gervaisi)--preliminary report. Indian J Med Res. 1979 Feb;69:244-6.

[91] Staphen S, Indrani R, Rao KA. Q fever antibodies in domestic animals in south Kanara-a preliminary report. Indian Journal of Medical Research. 1978;68(July):39-43.

[92] Stephen S, Chandrashekara I, KNA R. Complement fixing and agglutinating antibodies to Coxiella burnetii in several mammals of Karnataka State. 1979.

[93] Stephen S, Chandrashekara I, Laxminarayan Rao H, et al. Prevalence of human Q fever in south Kanara district, Karnataka. Indian Journal of Medical Research. 1980;71:510-515.

[94] Stephen S, Chandrashekara I, Rao K. Coxiellosis in fowls of Karnataka State. The Indian journal of medical research. 1980;71:363-364.

[95] Stephen S, Chandrashekara I, Rao K. Natural occurrence of Coxiella burnetii in the brown dog tick Rhipicephalus sanguineus. The Indian Journal of Medical Research. 1980;71:865-869.

[96] Stephen S, Chandrashekara I, Rao KG, et al. Natural occurrence of Coxiella burnetii in domestic mammals of Karnataka State. Indian J Med Res. 1980 Apr;71:516-21.

[97] Stephen S, Sangeetha B, Antony PX. Seroprevalence of coxiellosis (Q fever) in sheep & goat in Puducherry & neighbouring Tamil Nadu. Indian J Med Res. 2014 Dec;140(6):785-7.

[98] Sultana N, Pervin M, Sultana S, et al. Pathological study and molecular detection of zoonotic diseases in small ruminants at slaughterhouses in Mymensingh, Bangladesh [Article]. Veterinary World. 2022;15(9):2119-2130.

[99] Thompson CN, Blacksell SD, Paris DH, et al. Undifferentiated febrile illness in Kathmandu, Nepal. The American journal of tropical medicine and hygiene. 2015;92(4):875.

[100] Tshokey T, Stenos J, Durrheim DN, et al. Rickettsial Infections and Q Fever Amongst Febrile Patients in Bhutan. Trop Med Infect Dis. 2018 Jan 25;3(1).

[101] Tshokey T, Stenos J, Tenzin T, et al. Serological Evidence of Rickettsia, Orientia, and Coxiella in Domestic Animals from Bhutan: Preliminary Findings [Article]. Vector-Borne and Zoonotic Diseases. 2019;19(2):95-101.

[102] Ullah Q, Jamil H, Qureshi ZI, et al. Sero-epidemiology of Q fever (coxiellosis) in small ruminants kept at government livestock farms of Punjab, Pakistan [Article]. Pakistan Journal of Zoology. 2019;51(1):135-140.

[103] Ullah Q, El-Adawy H, Jamil T, et al. Serological and Molecular Investigation of Coxiella burnetii in Small Ruminants and Ticks in Punjab, Pakistan. Int J Environ Res Public Health. 2019 Nov 4;16(21).

[104] Vaidya V, Malik S, Kaur S, et al. Comparison of PCR, immunofluorescence assay, and pathogen isolation for diagnosis of Q fever in humans with spontaneous abortions. Journal of clinical microbiology. 2008;46(6):2038-2044.

[105] Vaidya VM, Malik SVS, Bhilegaonkar KN, et al. Prevalence of Q fever in domestic animals with reproductive disorders [Article]. Comparative Immunology, Microbiology and Infectious Diseases. 2010;33(4):307-321.

[106] Yadav M, Sethi M. Poikilotherms as reservoirs of Q-fever (Coxiella burnetii) in Uttar Pradesh. Journal of wildlife diseases. 1979;15(1):15-17.

[107] Yadav MP, Sethi MS. Sero-epidemiological studies on coxiellosis in animals and man in the state of Uttar Pradesh and Delhi (India). Int J Zoonoses. 1979 Dec;6(2):67-74.

[108] Yadav M, Sethi M. A study on the reservoir status of Q-fever in avifauna, wild mammals and poikilotherms in Uttar Pradesh (India). International journal of zoonoses. 1980;7(2):85-89.

[109] Yadav JP, Malik SVS, Dhaka P, et al. Seasonal variation in occurrence of Coxiella burnetii infection in buffaloes slaughtered in India [Article]. Biological Rhythm Research. 2019;52(8):1-7.

[110] Yadav JP, Malik SVS, Dhaka P, et al. Coxiella burnetii in cattle and their human contacts in a gaushala (cattle shelter) from India and its partial com1 gene sequence-based phylogenetic analysis [Article]. Animal Biotechnology. 2021.

[111] Zahid MU, Hussain MH, Saqib M, et al. Seroprevalence of Q Fever (Coxiellosis) in Small Ruminants of Two Districts in Punjab, Pakistan. Vector Borne Zoonotic Dis. 2016 Jul;16(7):449-54.

[112] Zaibaq-Krill J, Weber FH. Cholestatic jaundice: A rare presentation of coxiella burnetii [Conference Abstract]. American Journal of Gastroenterology. 2019;114:S1348-S1349.
