# Supplementary material for: Prevalence and Risk Factors of Coxiellosis at the Human–Animal–Environment Interface in the South Asian Countries: A Systematic Review and Meta-Analysis
Source: Transbound Emerg Dis. 2025 Jan 31;2025:2890693. doi: 10.1155/tbed/2890693 (PMC12016896; doi:10.1155/tbed/2890693)
Supplement: Supporting Information 5 — Funnel plots depict the seroprevalence and carrier prevalence of Coxiellosis in humans and animals of the South Asian countries. [file 2890693.f5.docx]

Title: Prevalence of Coxiellosis in livestock ruminants in South Asian countries: a systematic review and meta-analysis

**Supplementary file 5**


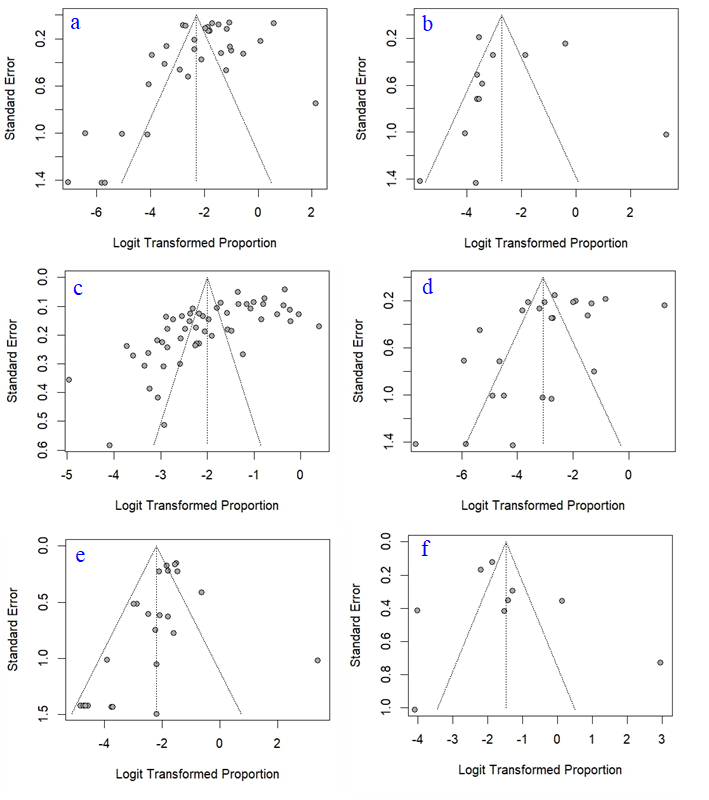


**Figure:** Funnel plots depict the seroprevalence and pathogen prevalence of coxiellosis in humans and animals of South Asian countries. The first panel illustrates seroprevalence (a) and pathogen (b) prevalence in humans; the second panel depicts the seroprevalence (c) and pathogen (d) prevalence in ruminants; the third panel shows the seroprevalence (e) in non-ruminant mammals, and non-mammals (f).
