# Supplementary material for: Prevalence and Risk Factors of Coxiellosis at the Human–Animal–Environment Interface in the South Asian Countries: A Systematic Review and Meta-Analysis
Source: Transbound Emerg Dis. 2025 Jan 31;2025:2890693. doi: 10.1155/tbed/2890693 (PMC12016896; doi:10.1155/tbed/2890693)

**Prevalence and risk factors of Coxiellosis at the Human-Animal-Environment interface in the South Asian countries: a systematic review and meta-analysis**

**Supplementary file 6: forest plots**

# Forest plots of meta-analysis

## Q fever in humans

### Seroprevalence and associated risk factors


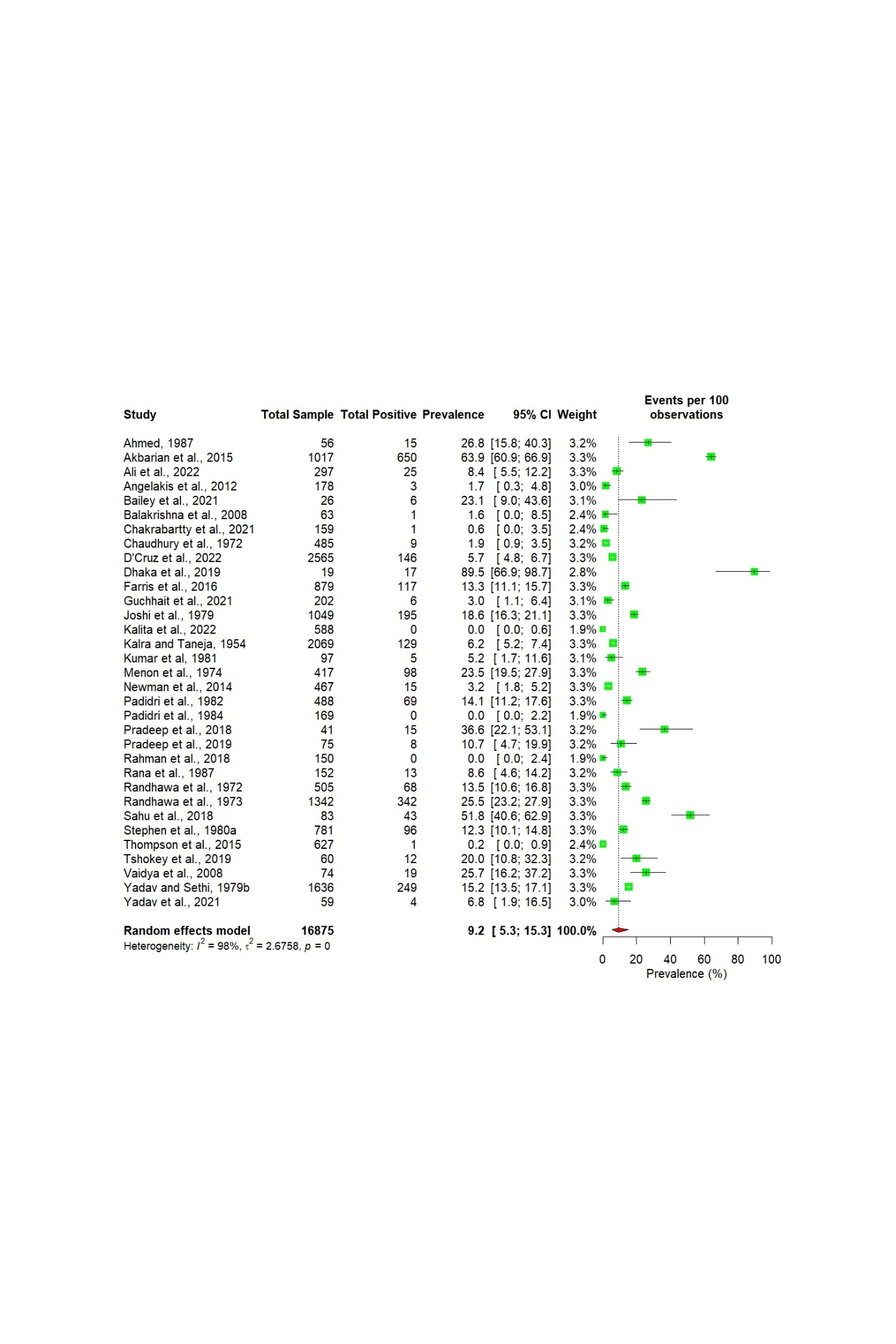


#### Country-wise difference in seroprevalence


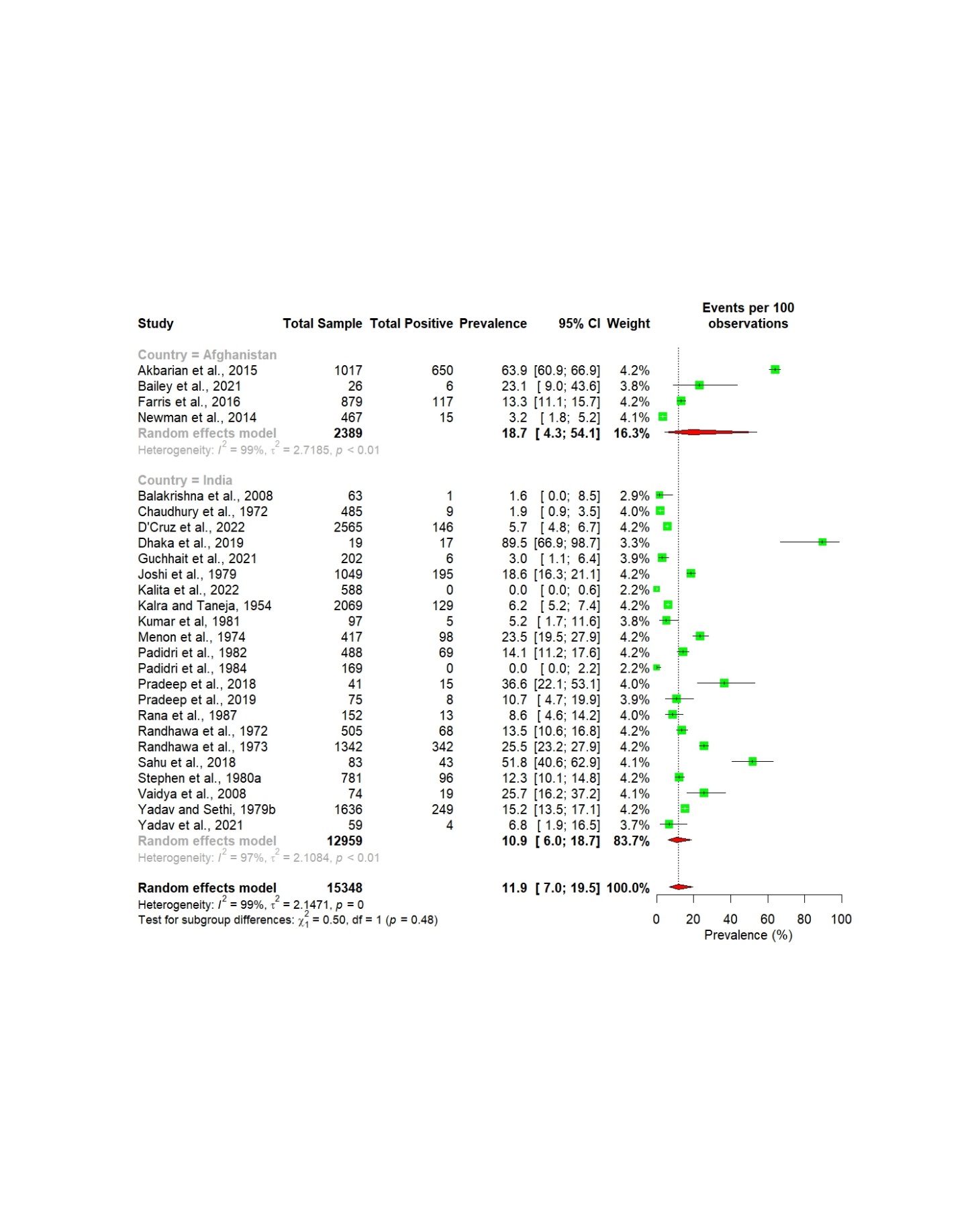


#### Sex-wise difference in seroprevalence


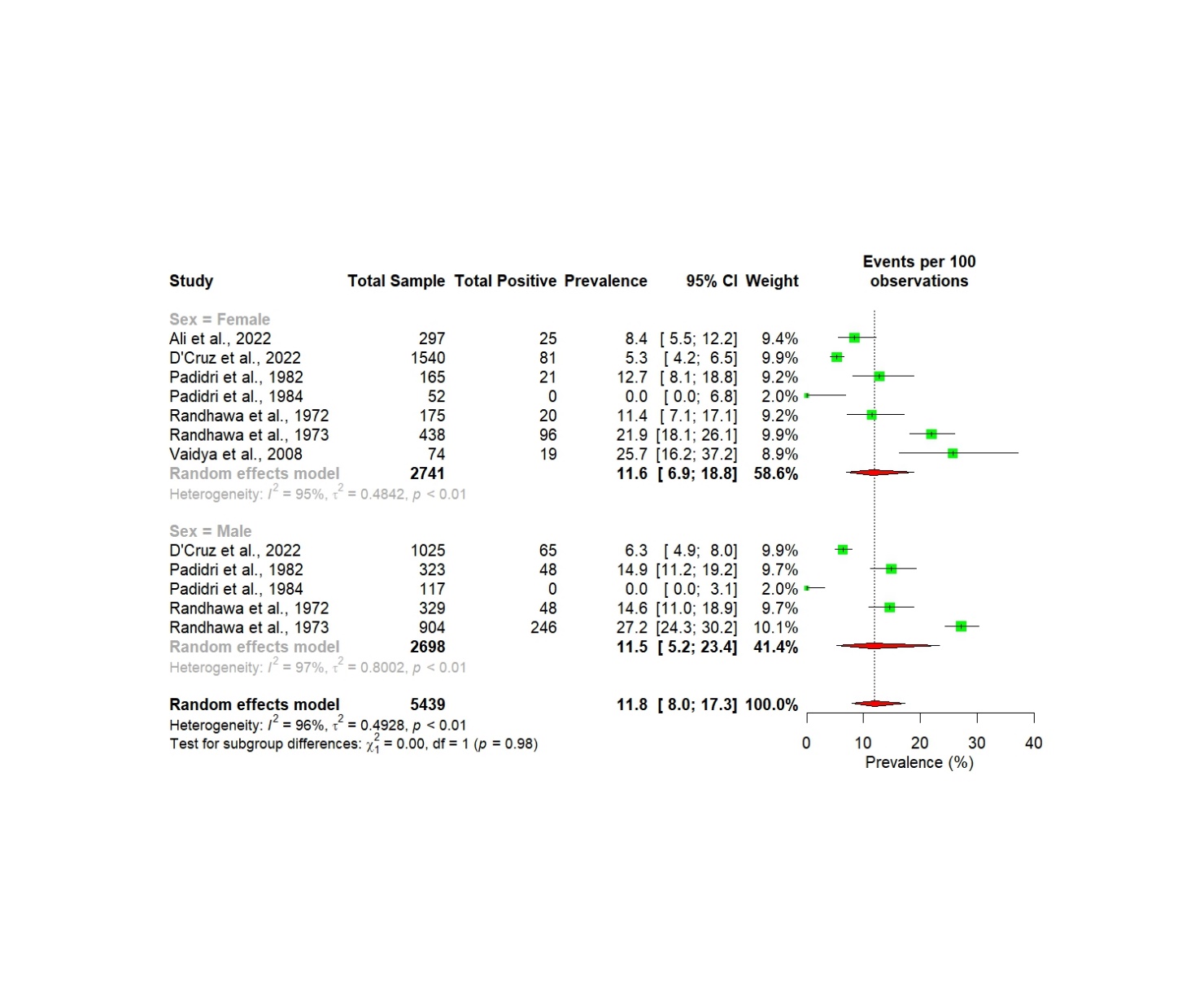


#### Age-wise difference in seroprevalence


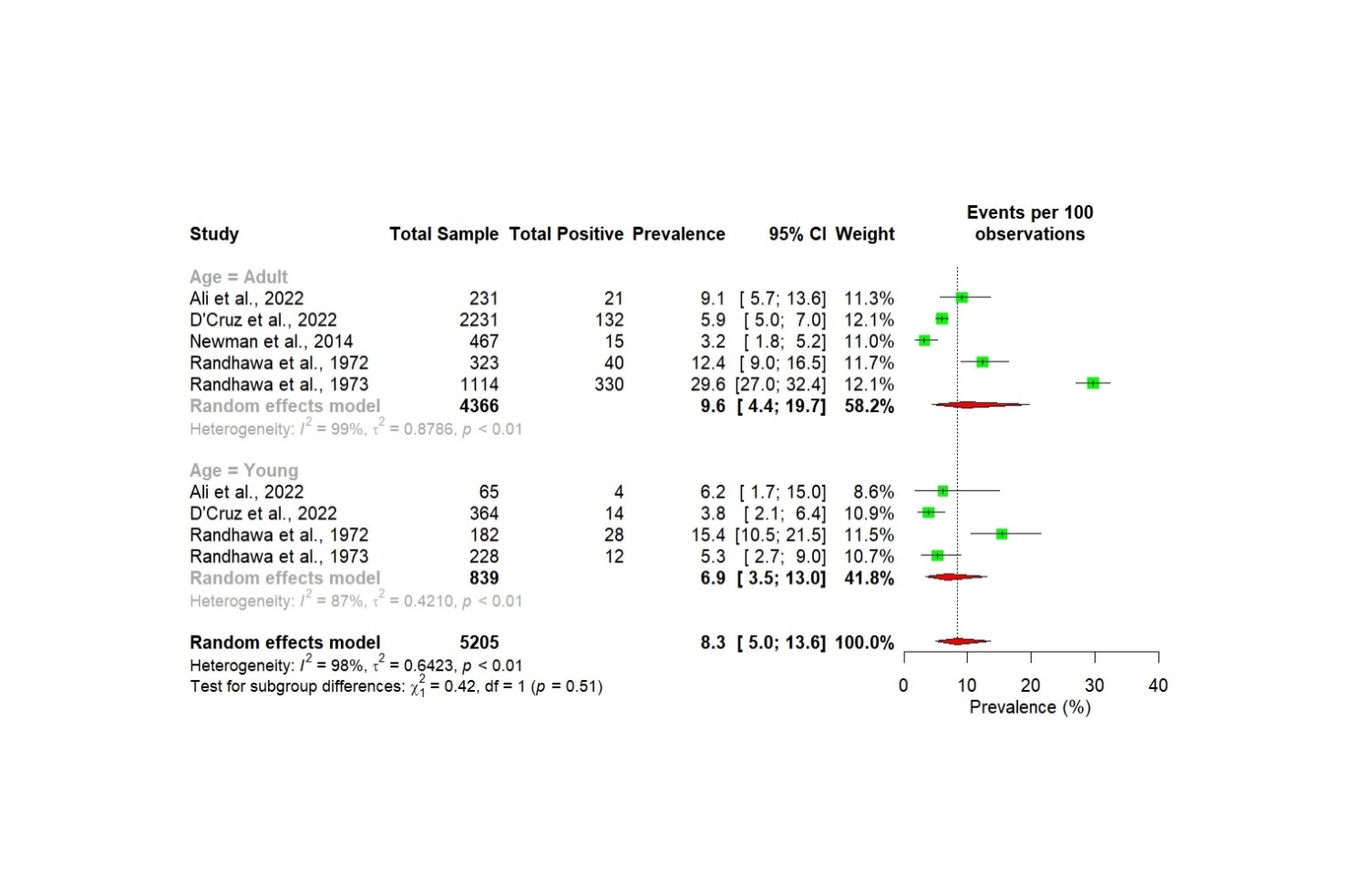


### Carrier prevalence


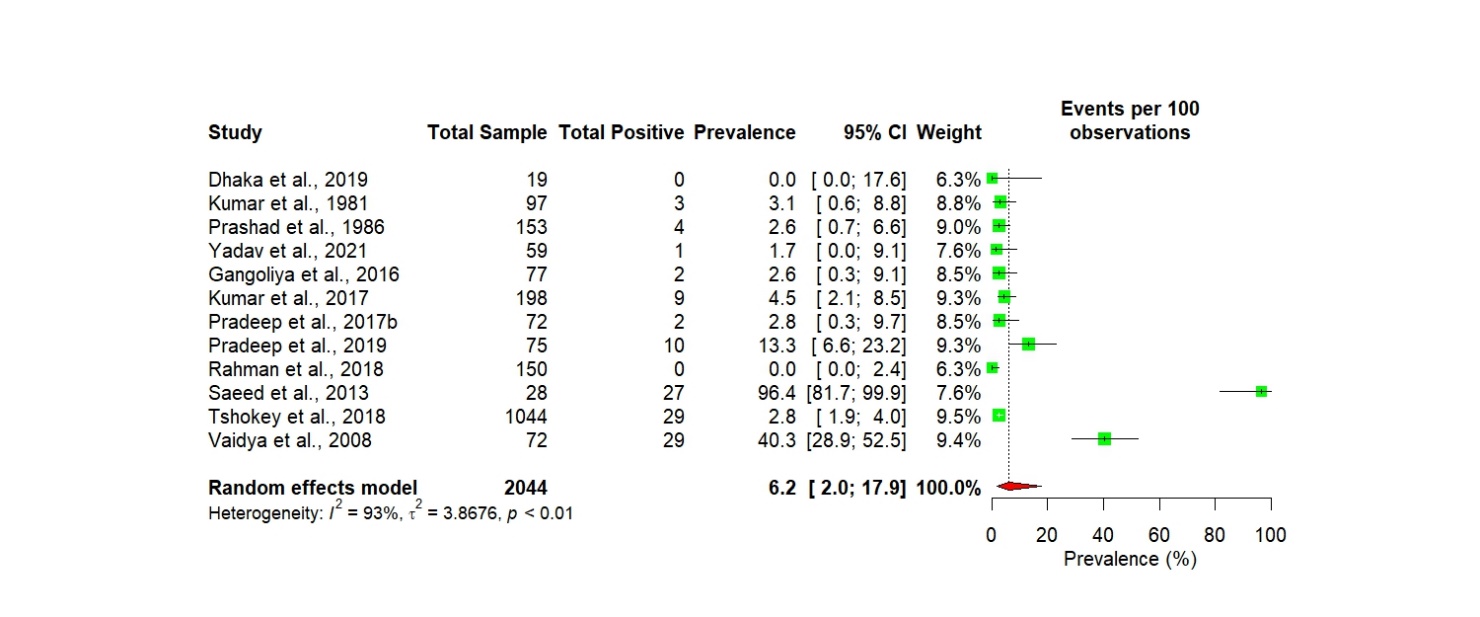


## Coxiellosis in ruminants

### Herd level seroprevalence and associated risk factors


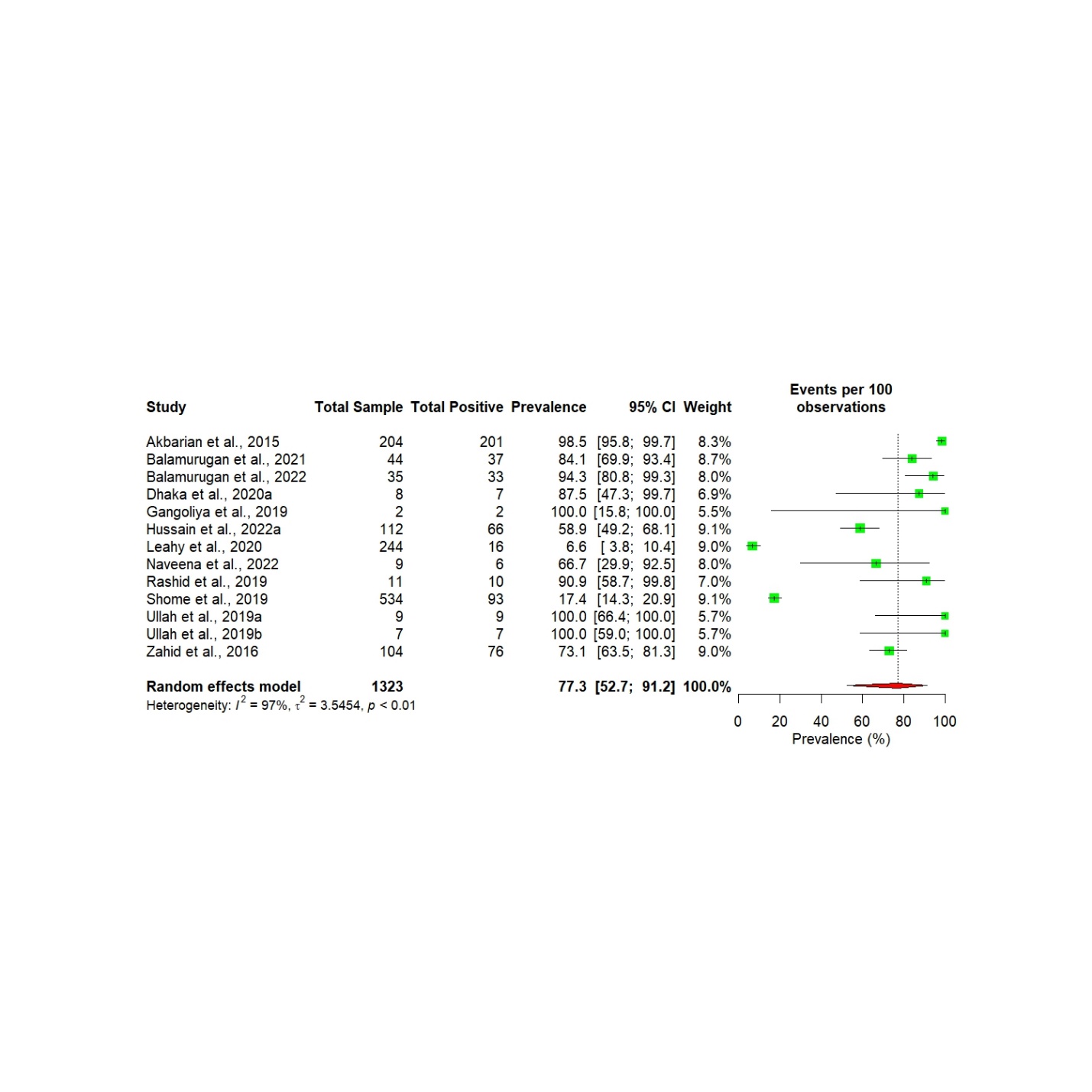


#### Large ruminant vs small ruminant herd level seroprevalence


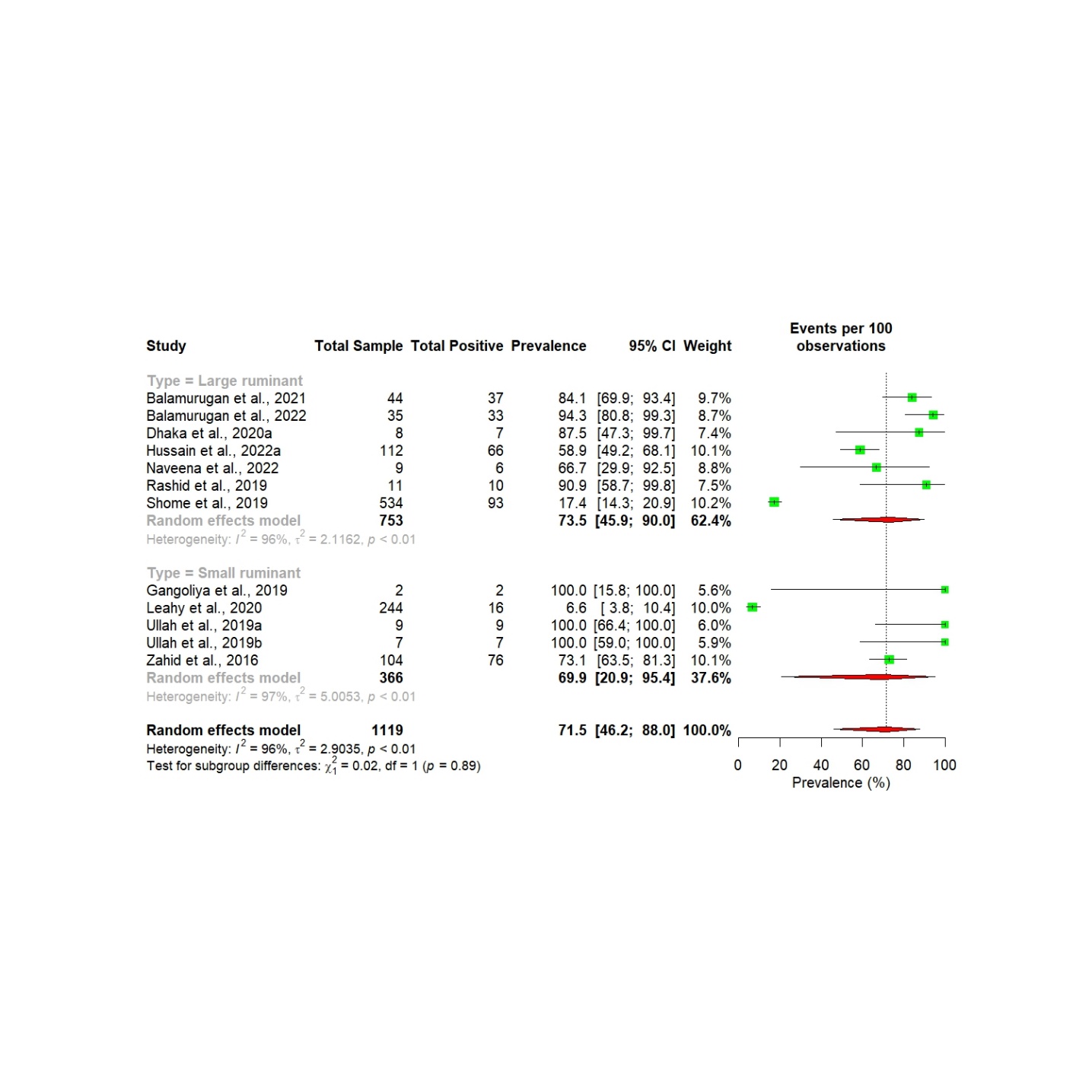


#### Species herd-level seroprevalence


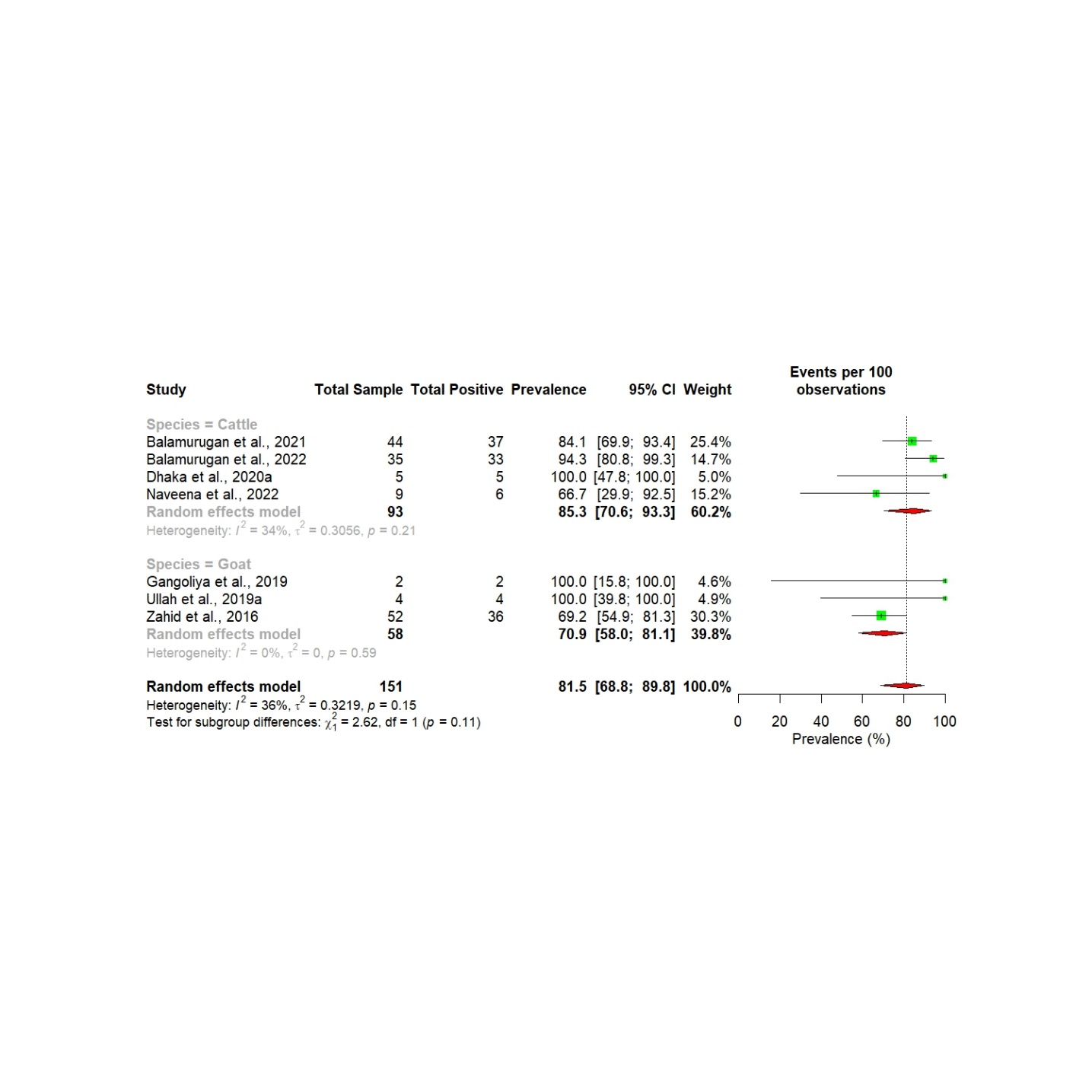


#### Country of origin herd-level seroprevalence


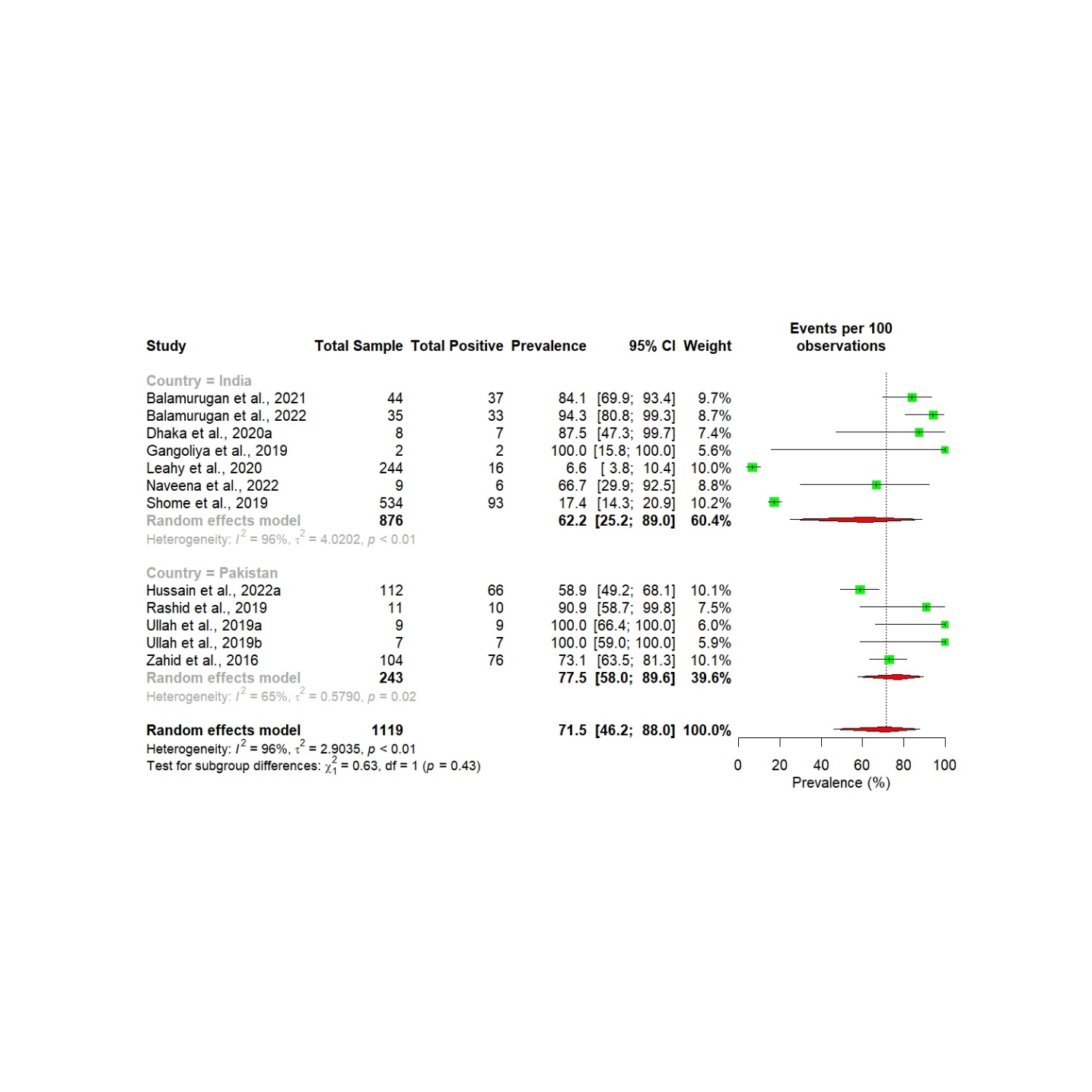


### Herd level carrier prevalence


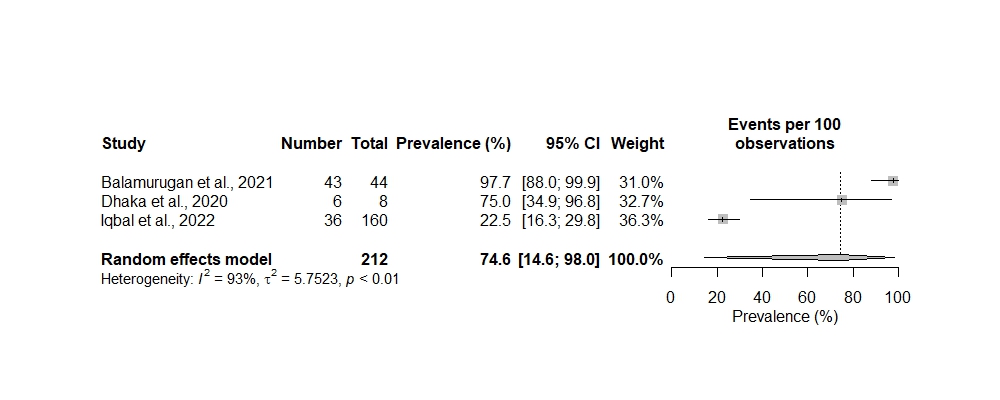


### Ruminant level seroprevalence and associated risk factors

#### Seroprevalence at individual ruminant level


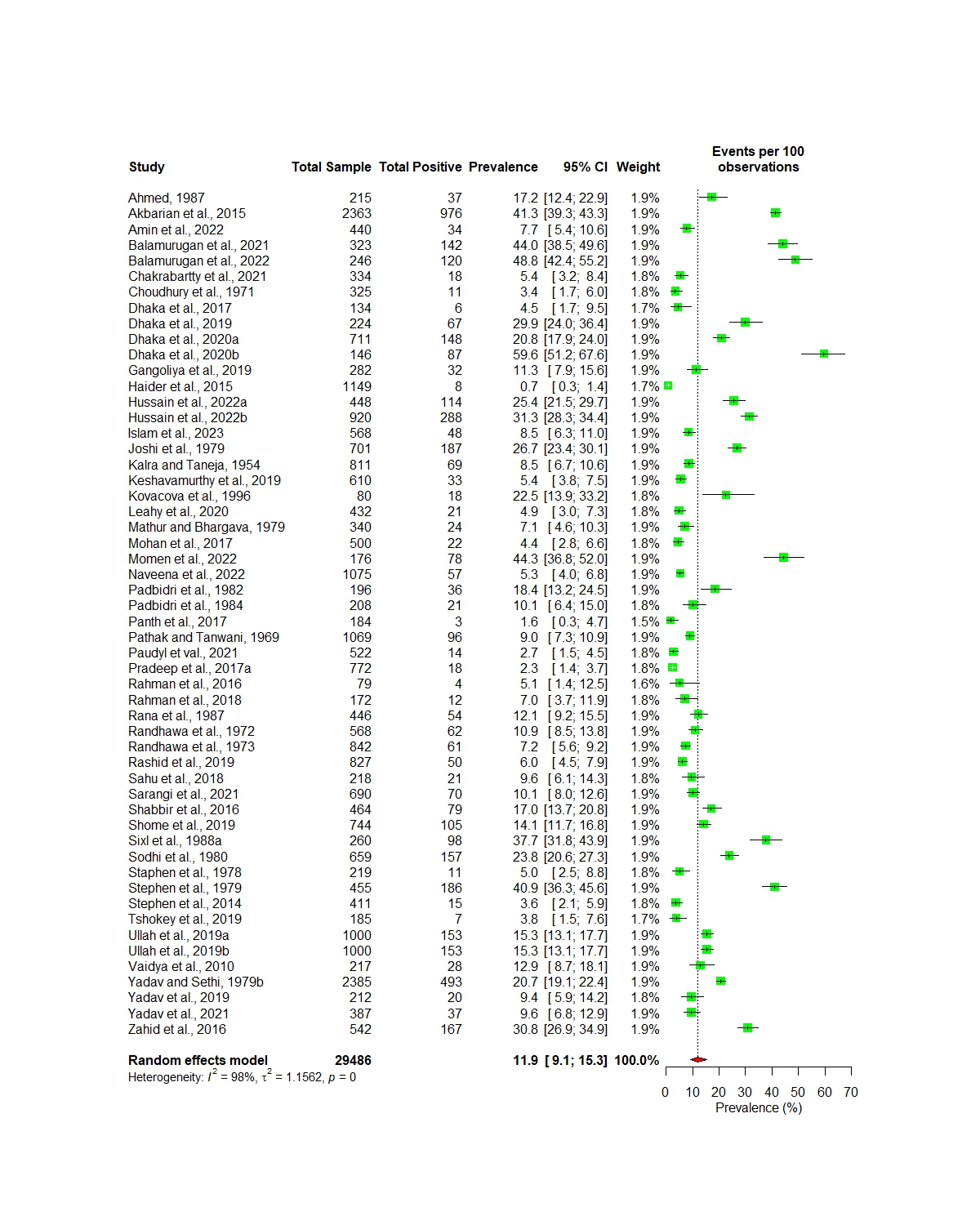


#### Seroprevalence based on ruminant type (Large ruminant vs small ruminant)


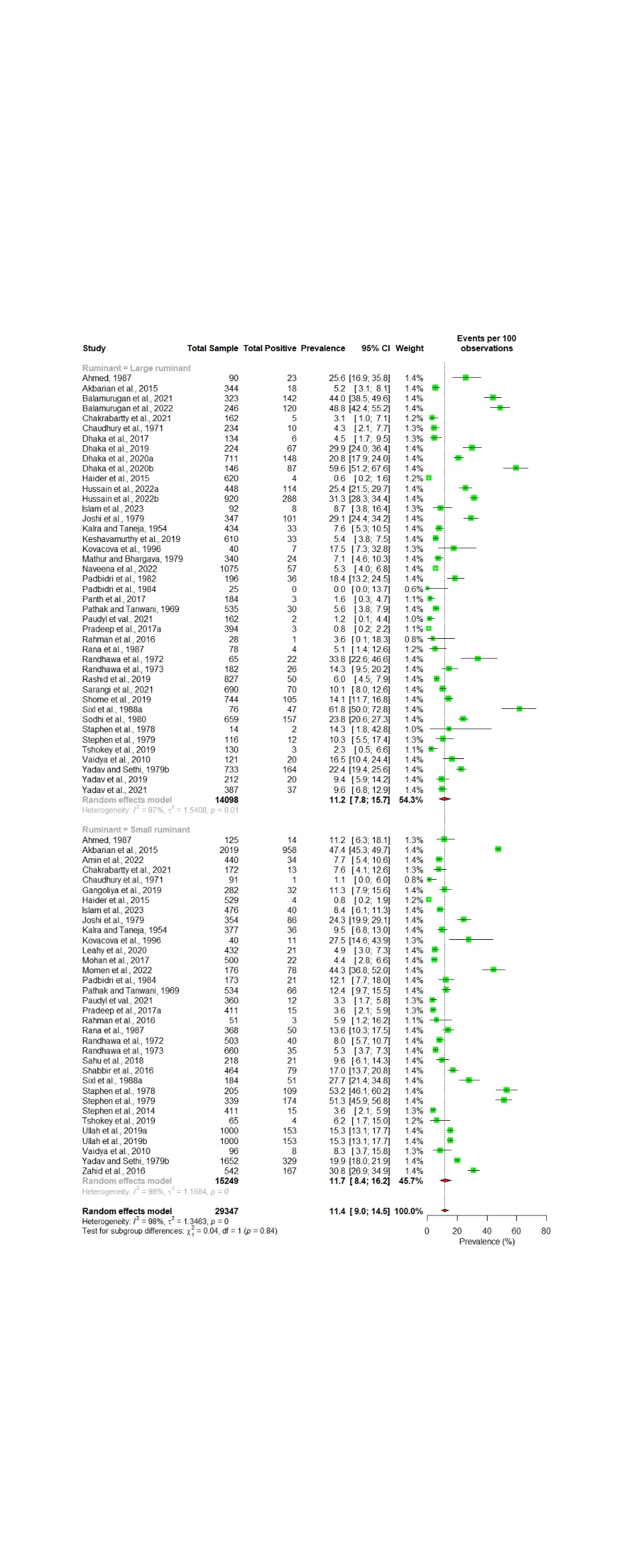


#### Species


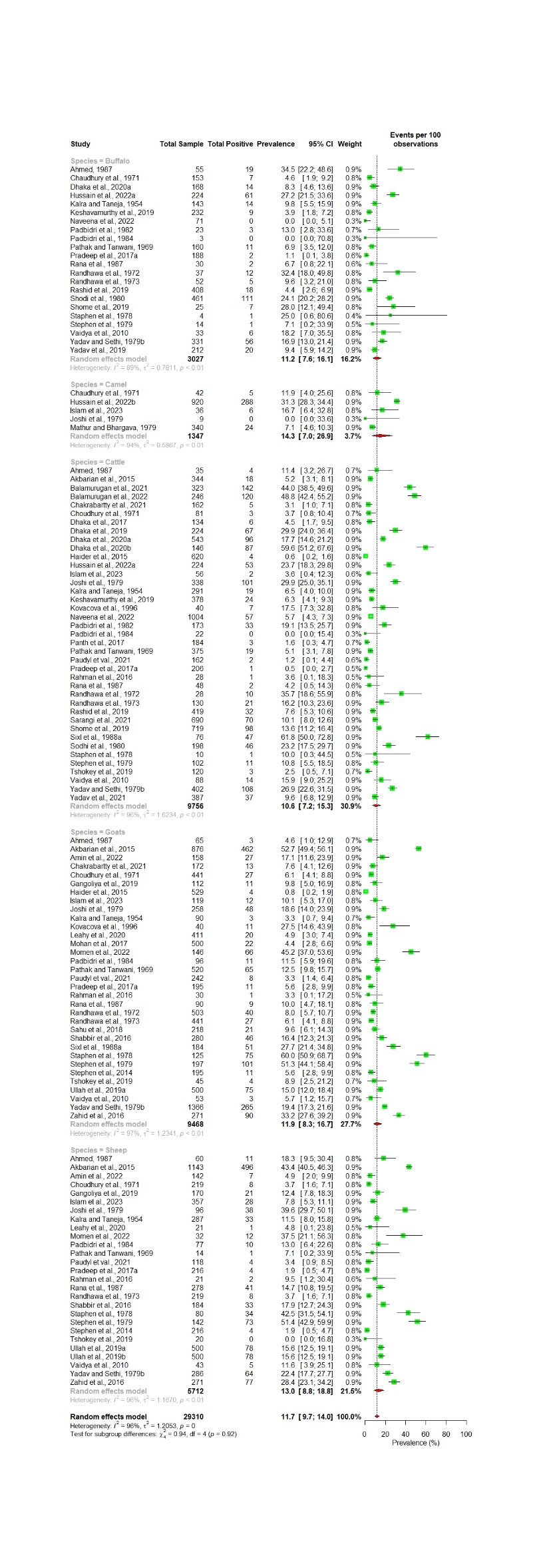


Figure 1: Species-wise seroprevalence of coxiellosis in South Asia

#### Country of origin


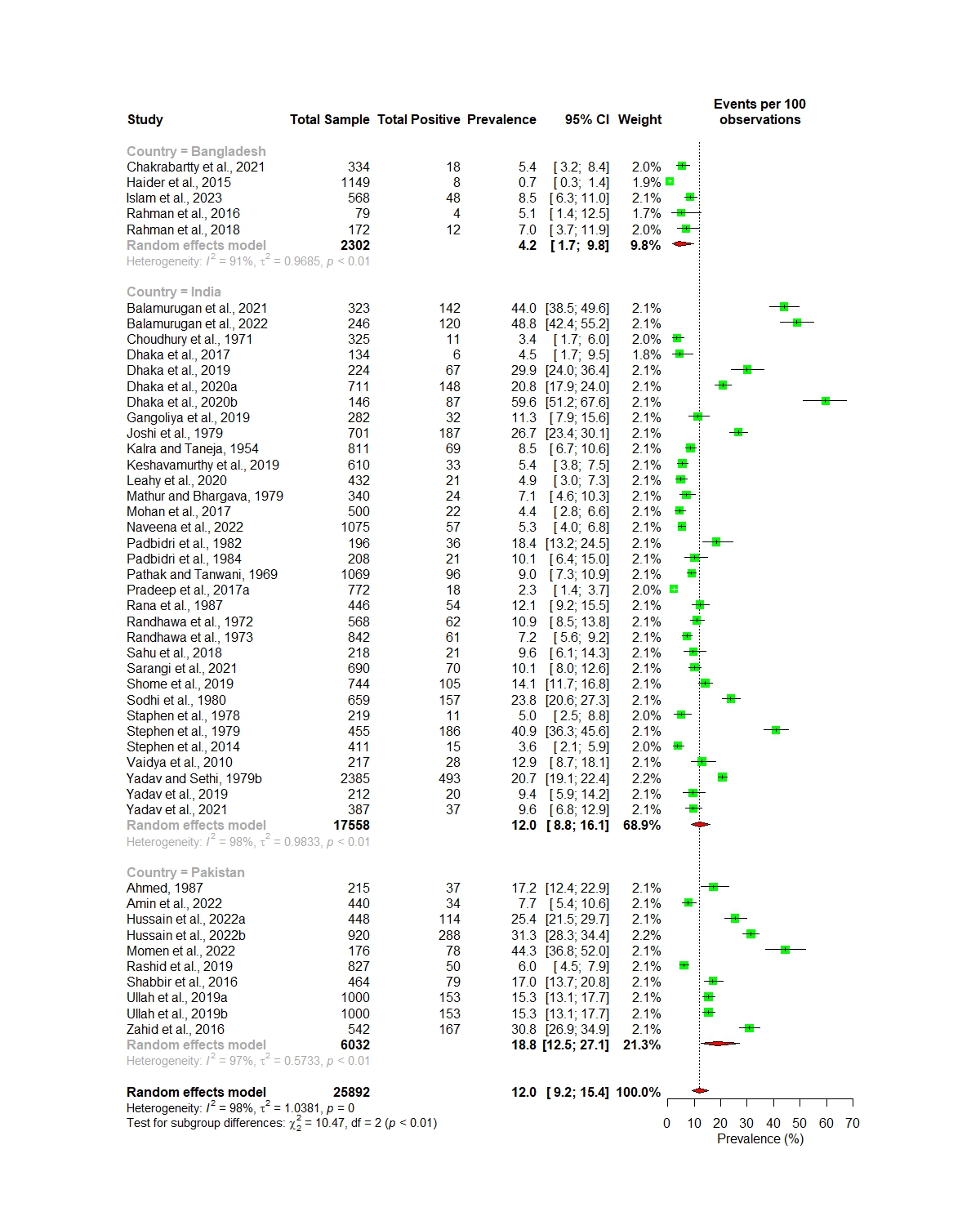


#### Ruminant age


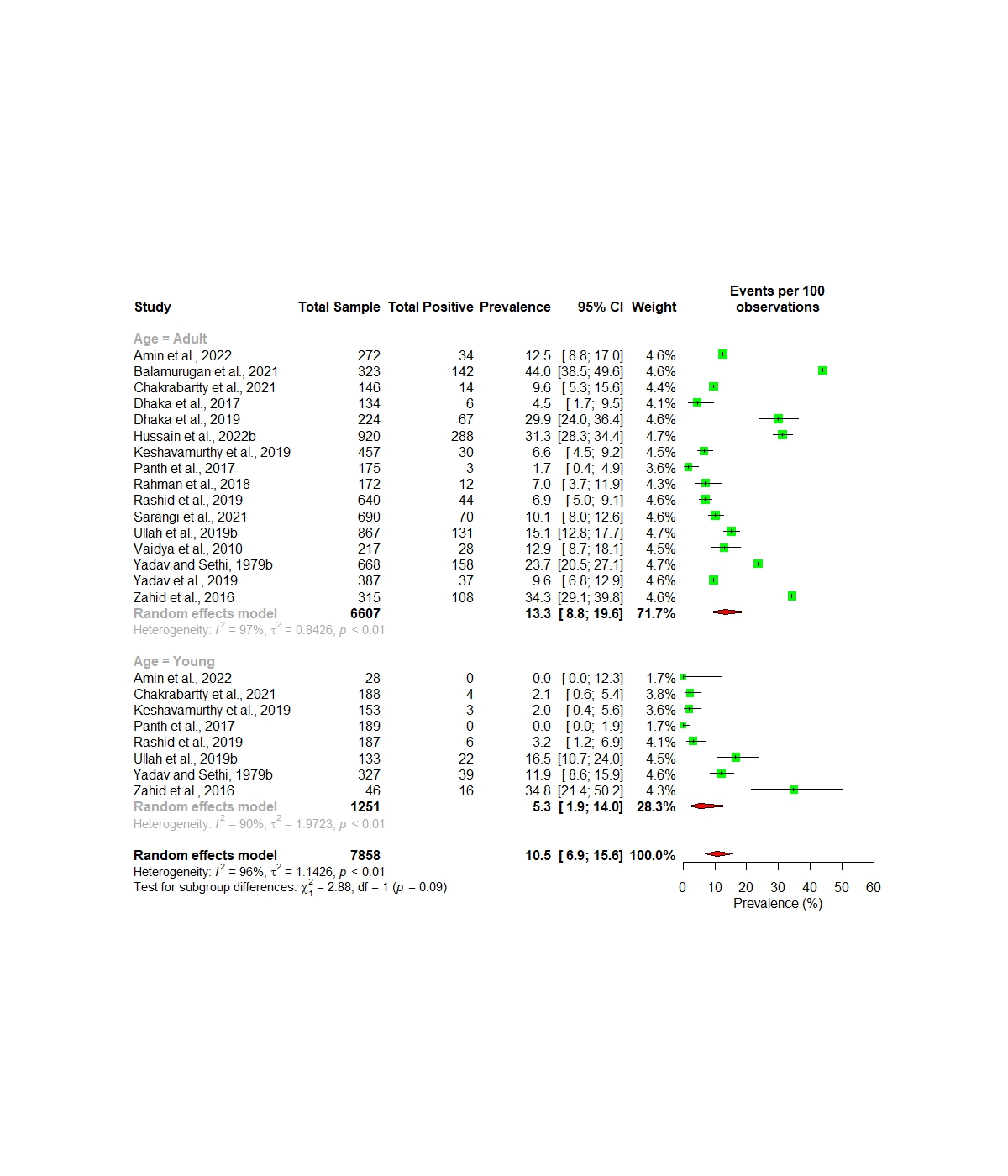


#### Ruminant sex


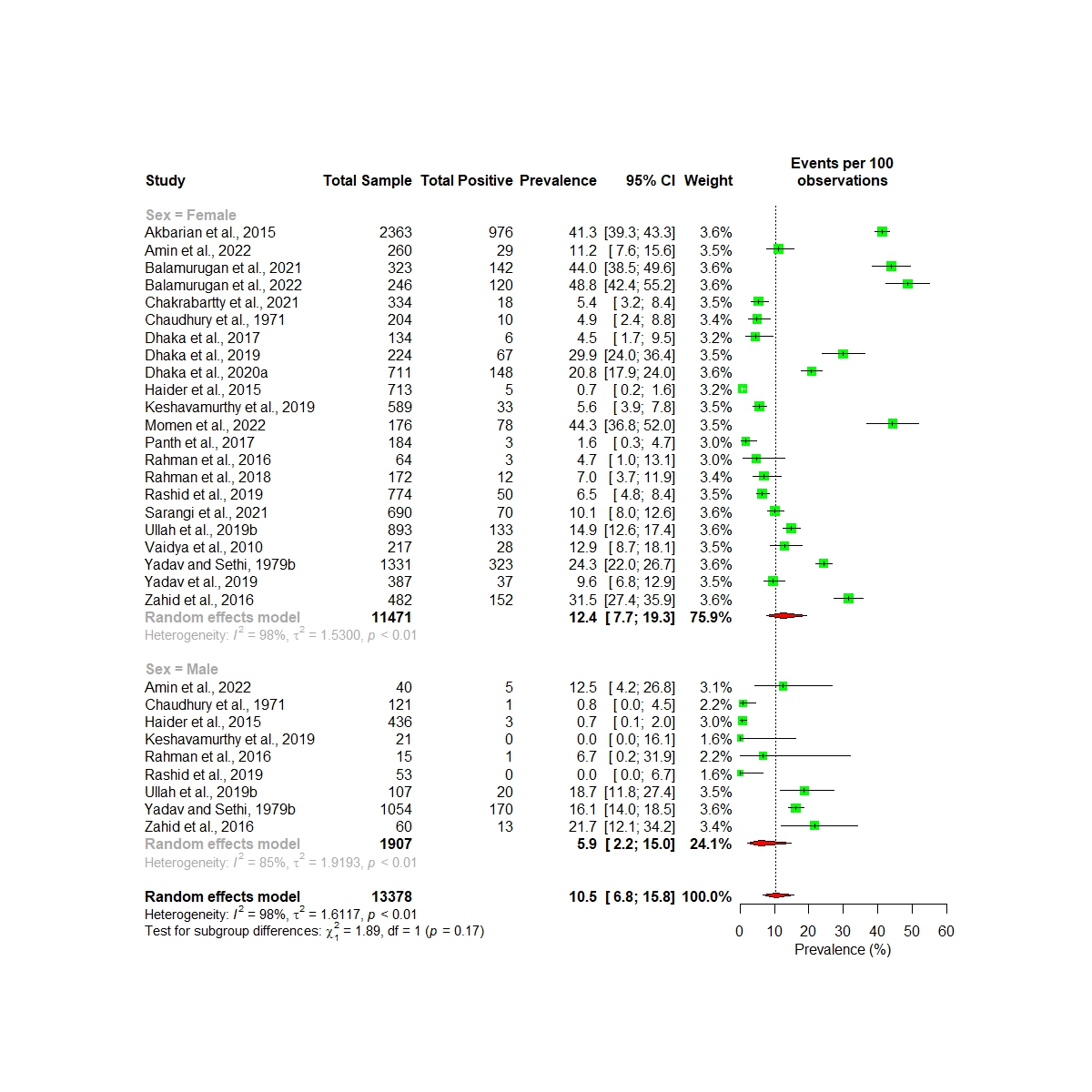


#### Breed


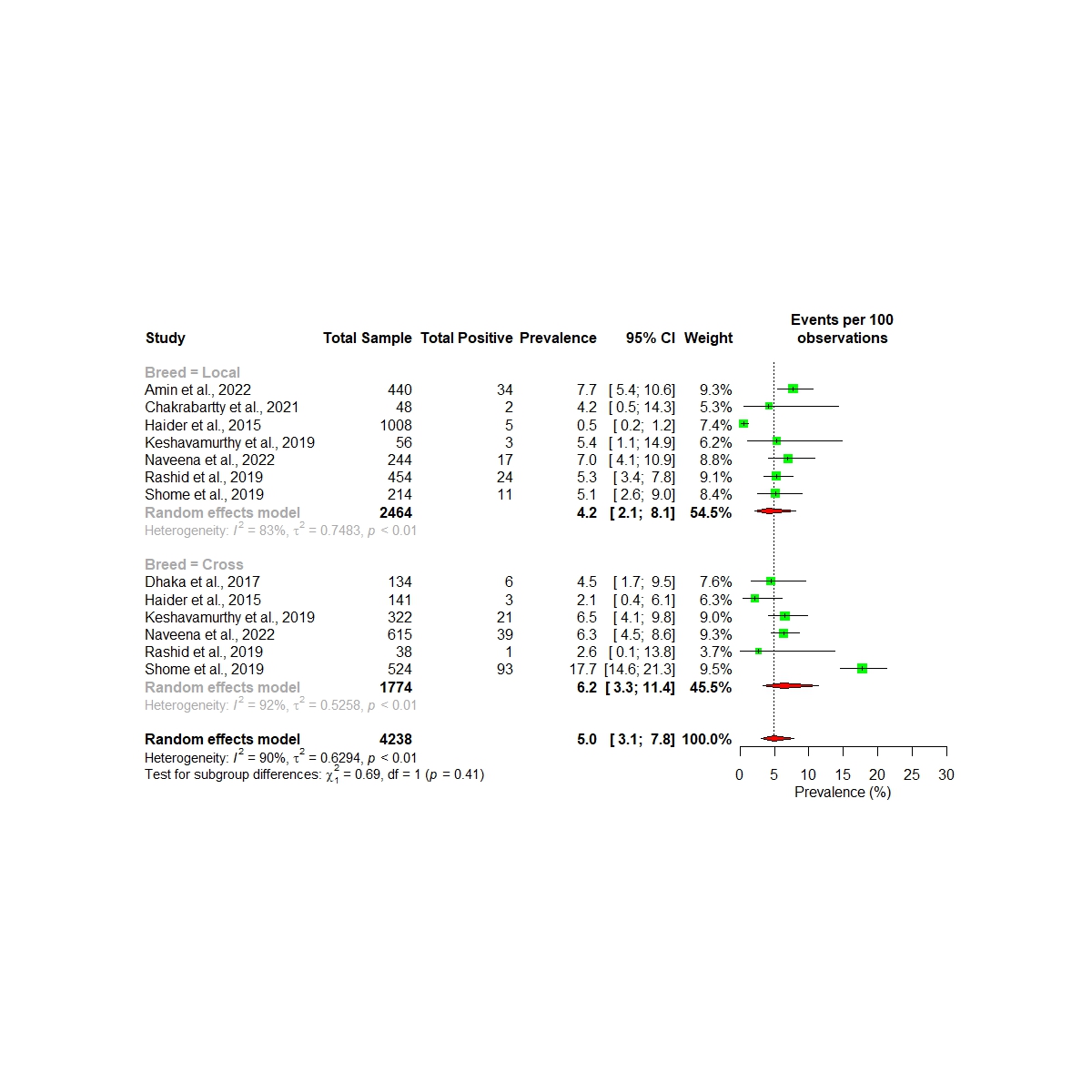


#### Ruminant pregnancy


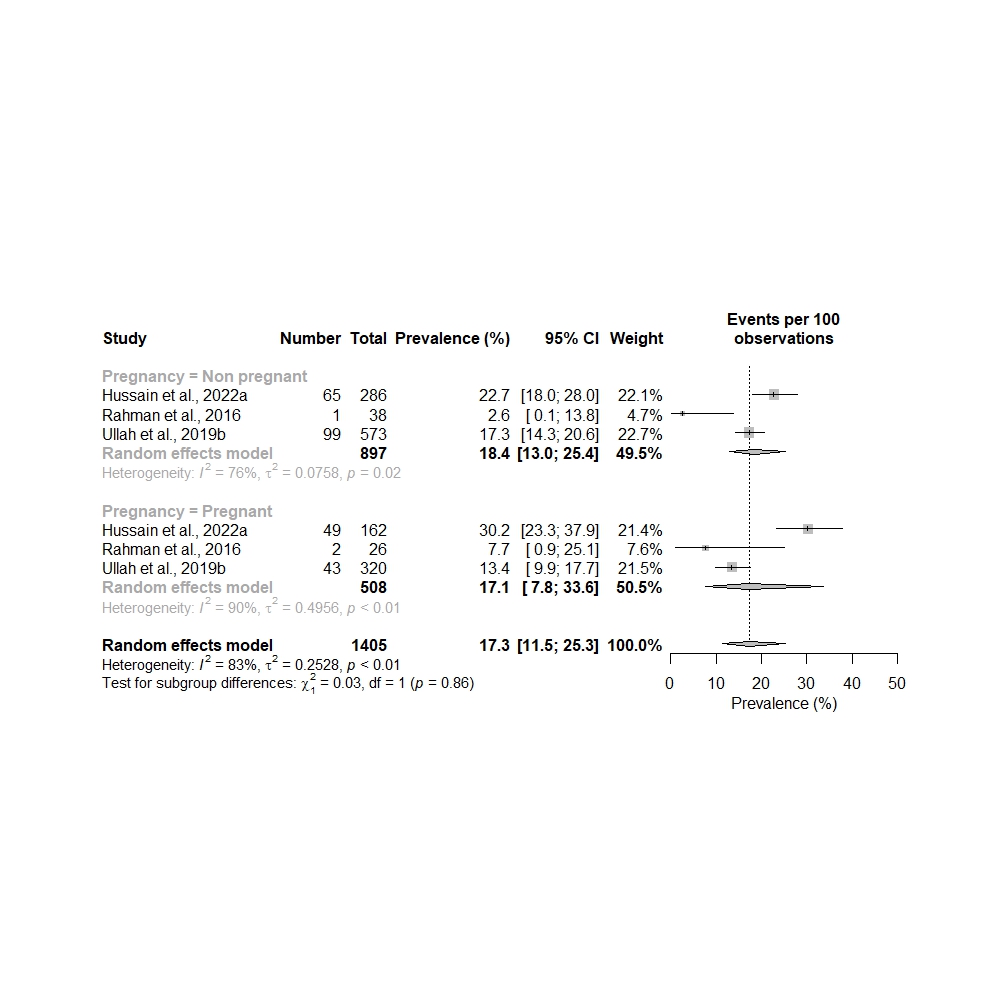


#### Ruminant parity


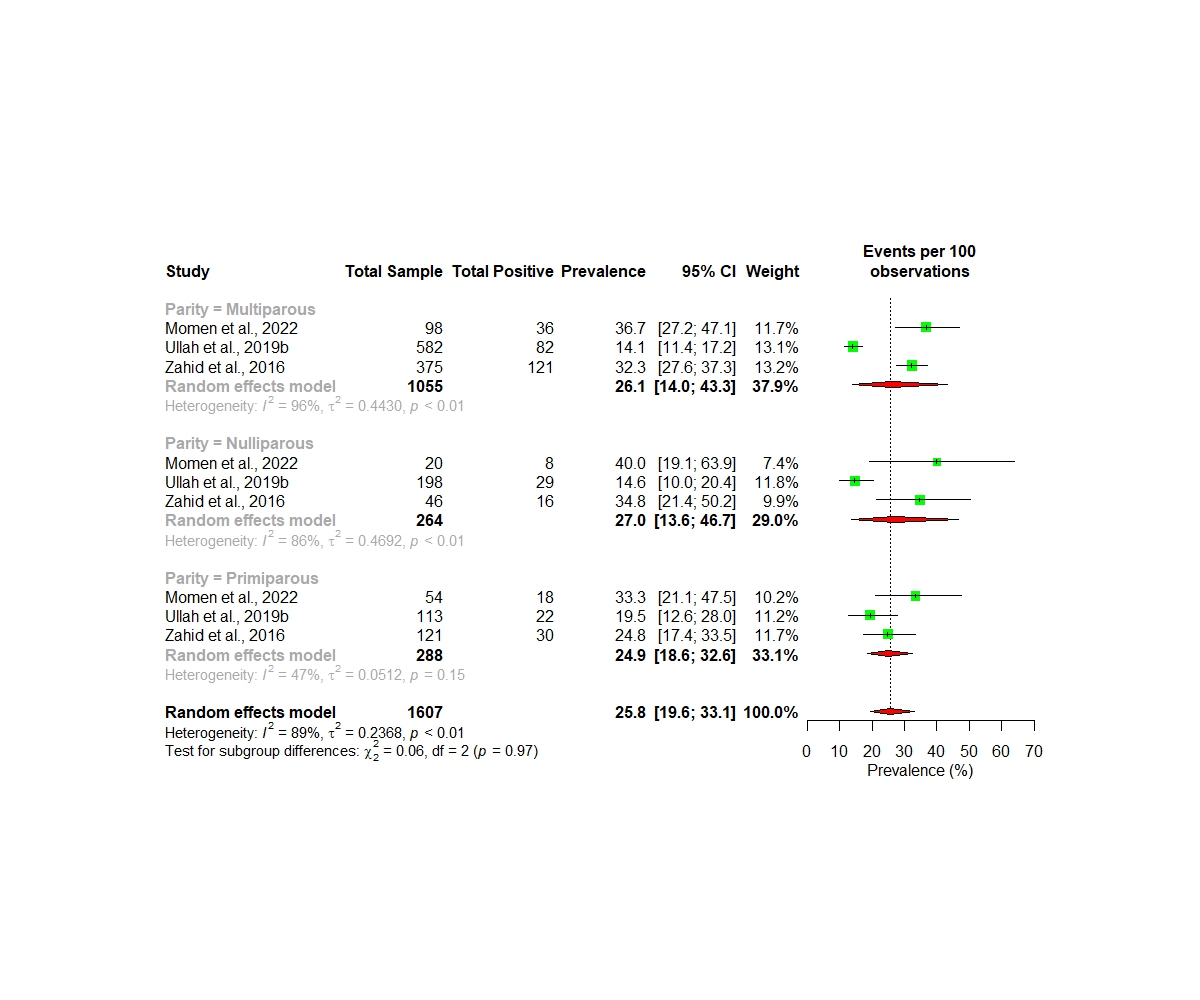


#### Ruminant body condition


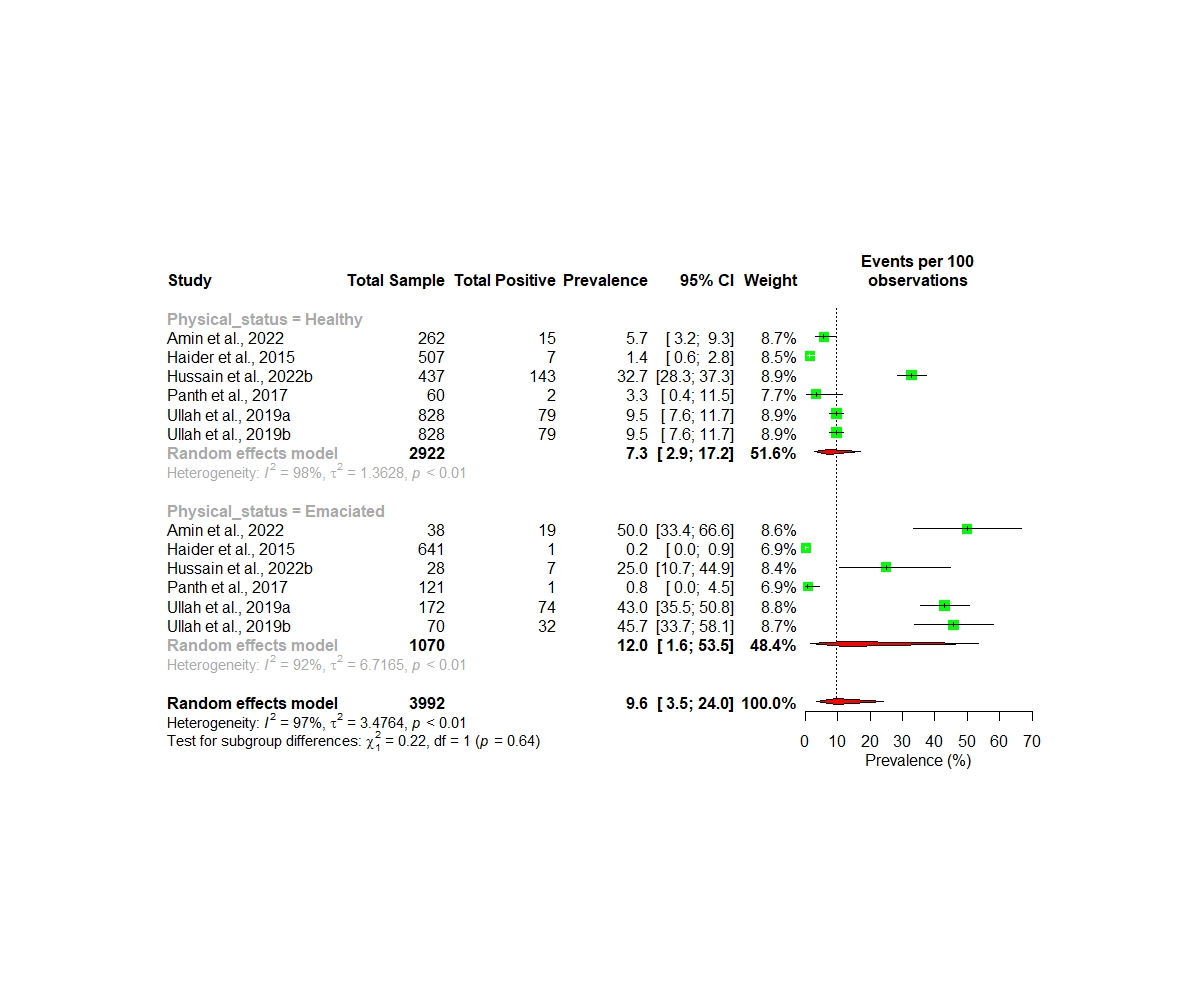


#### Ruminant grazing


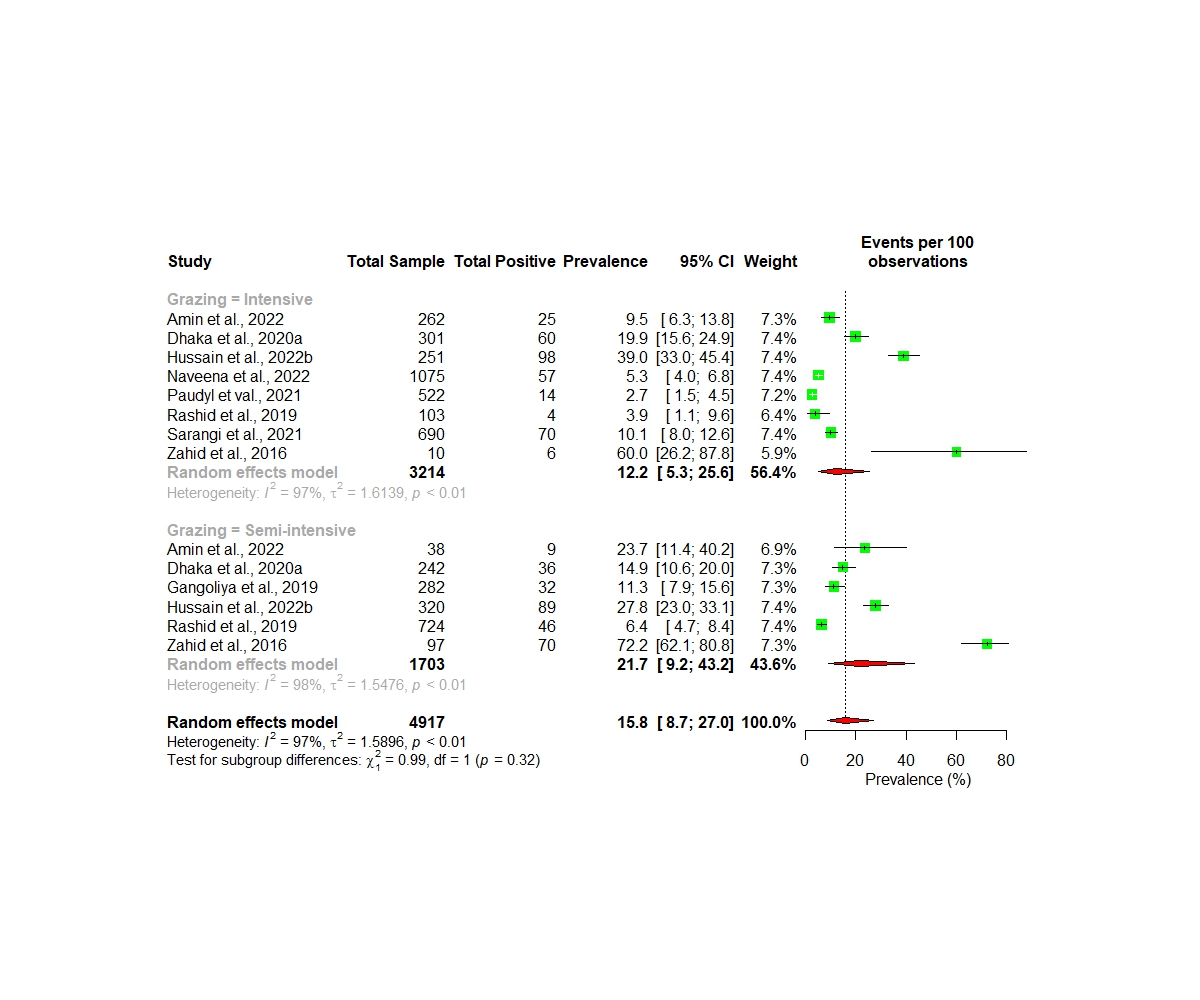


#### Ruminant biosafety


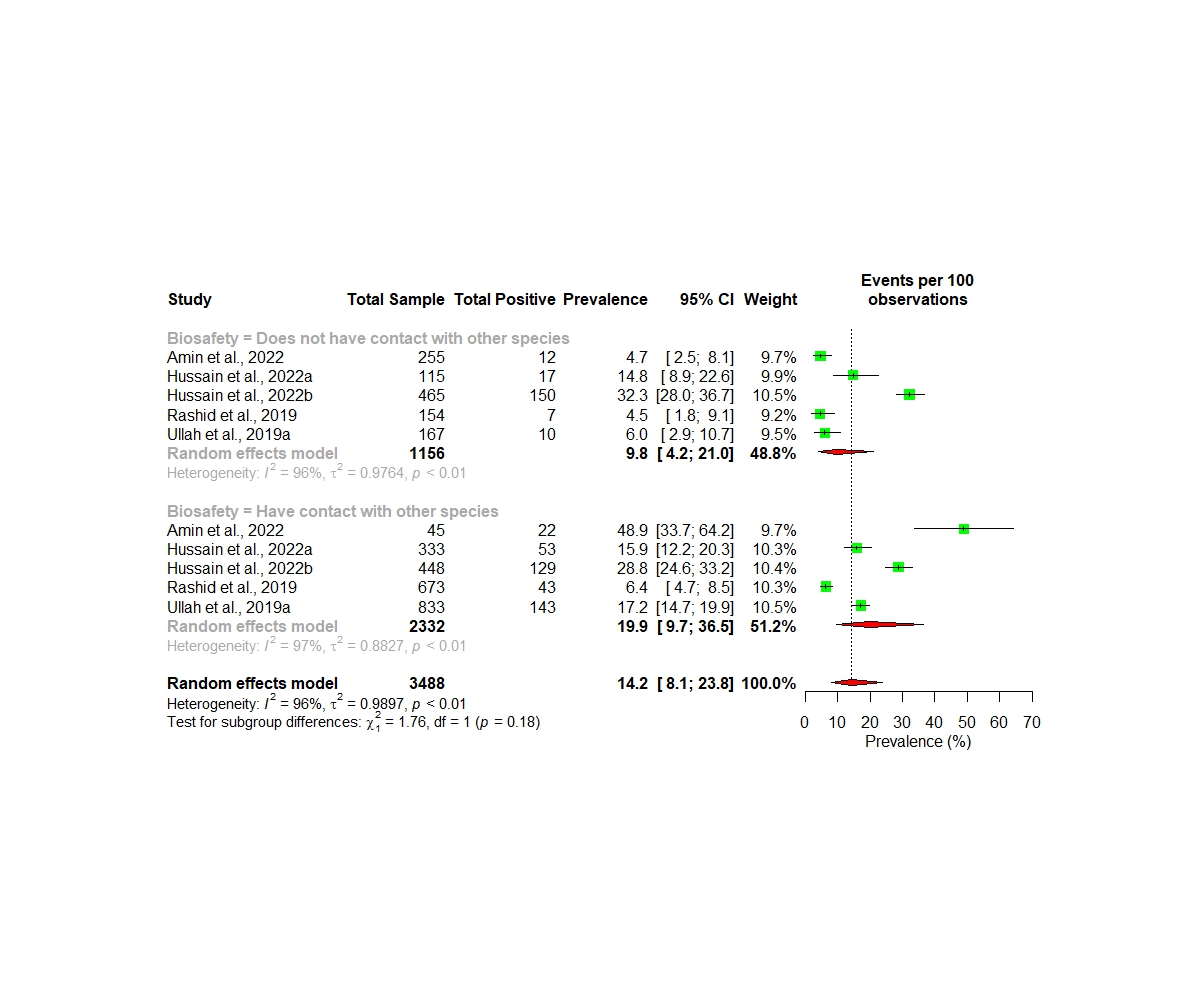


#### Ruminant tick infestation


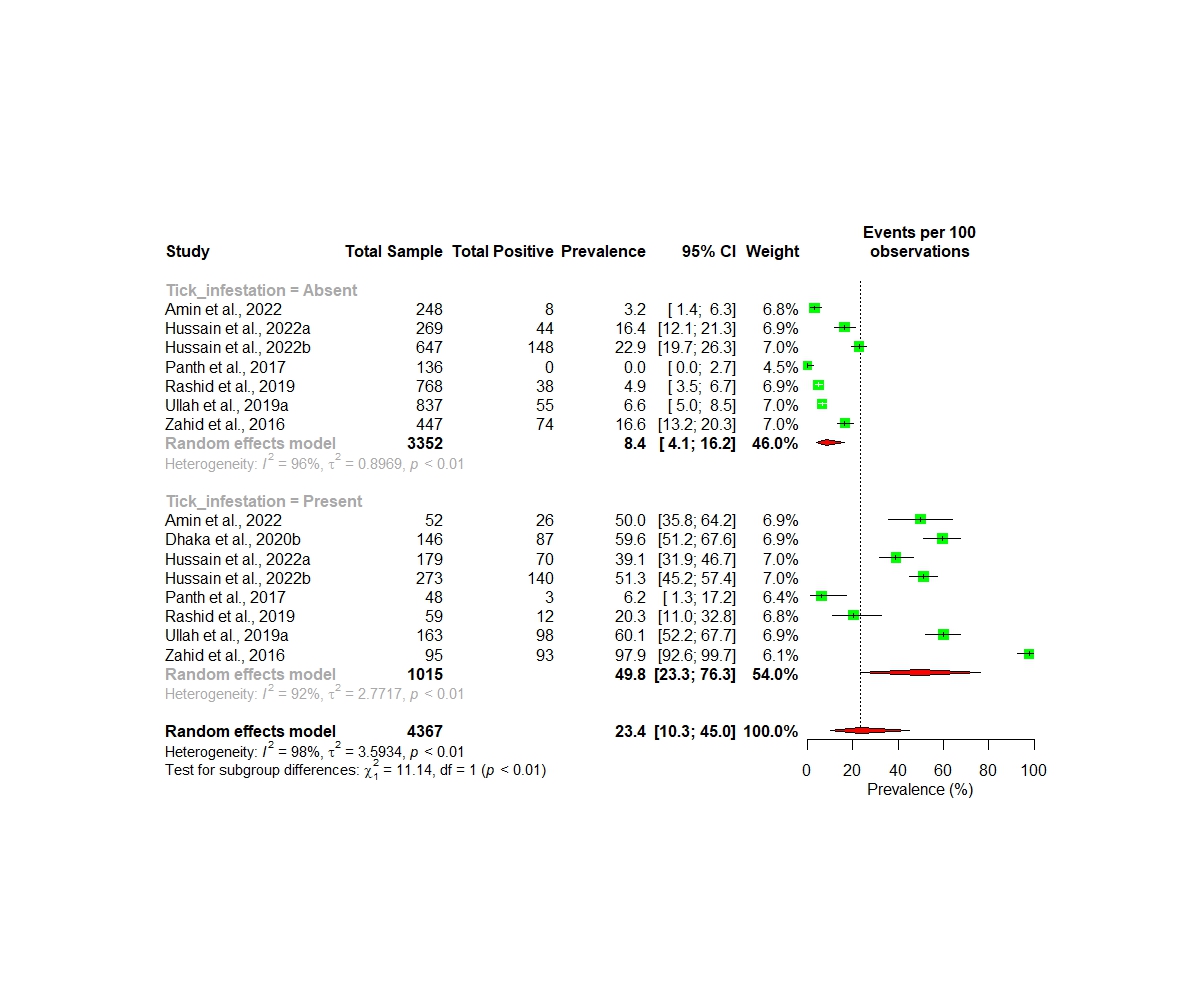


#### Ruminant season


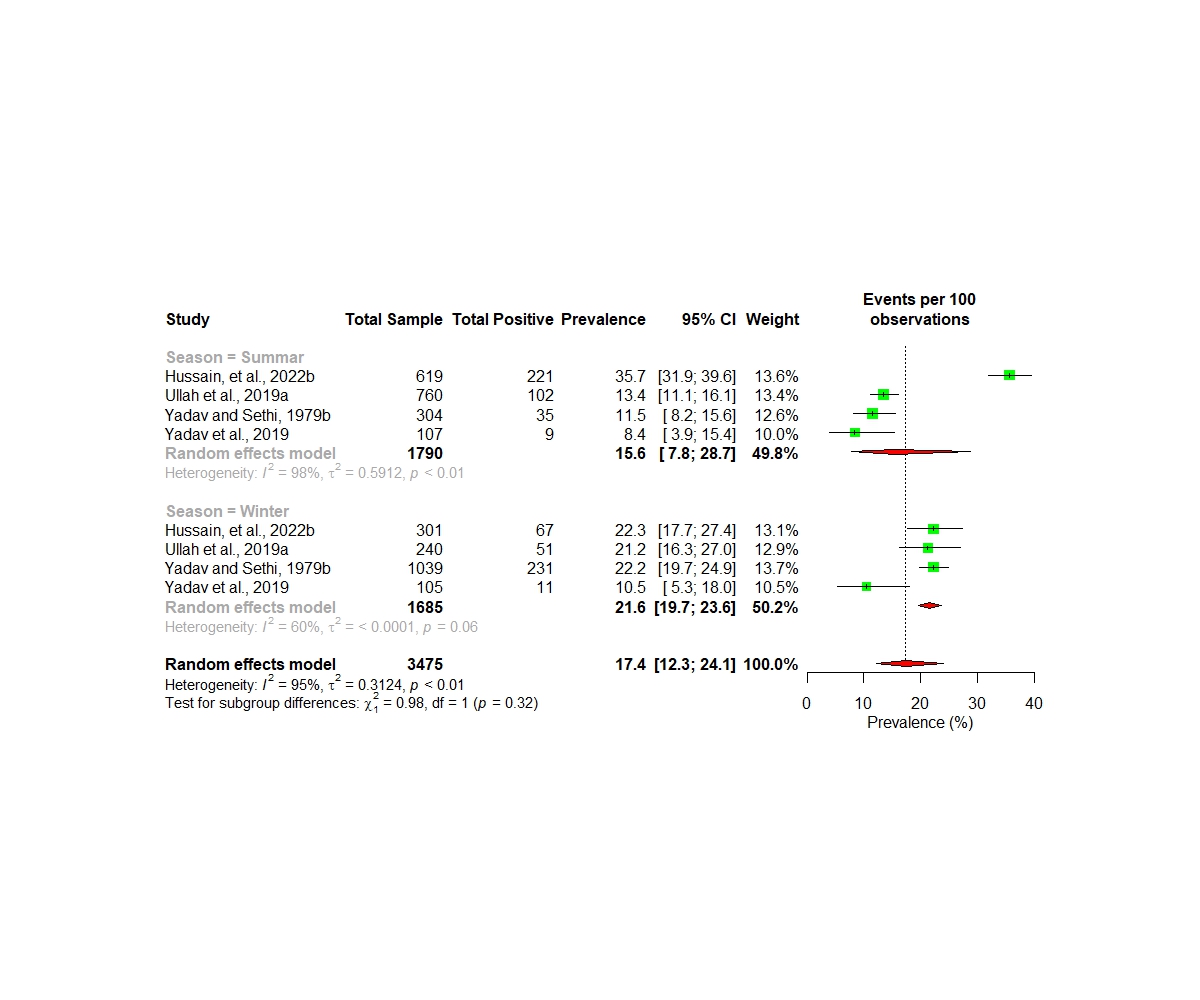


#### Ruminant history of reproductive disorder


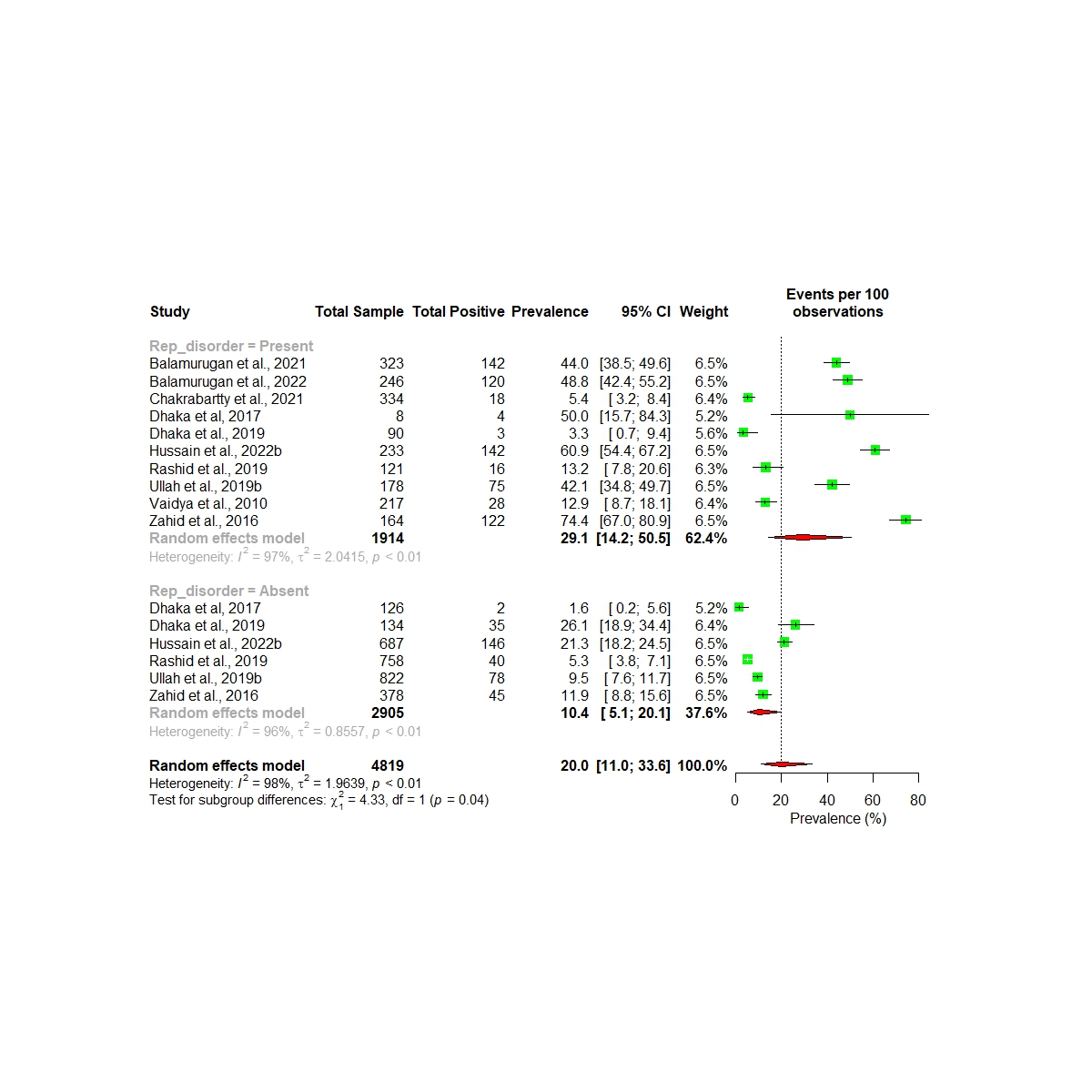


#### Ruminant_type of reproductive disorders


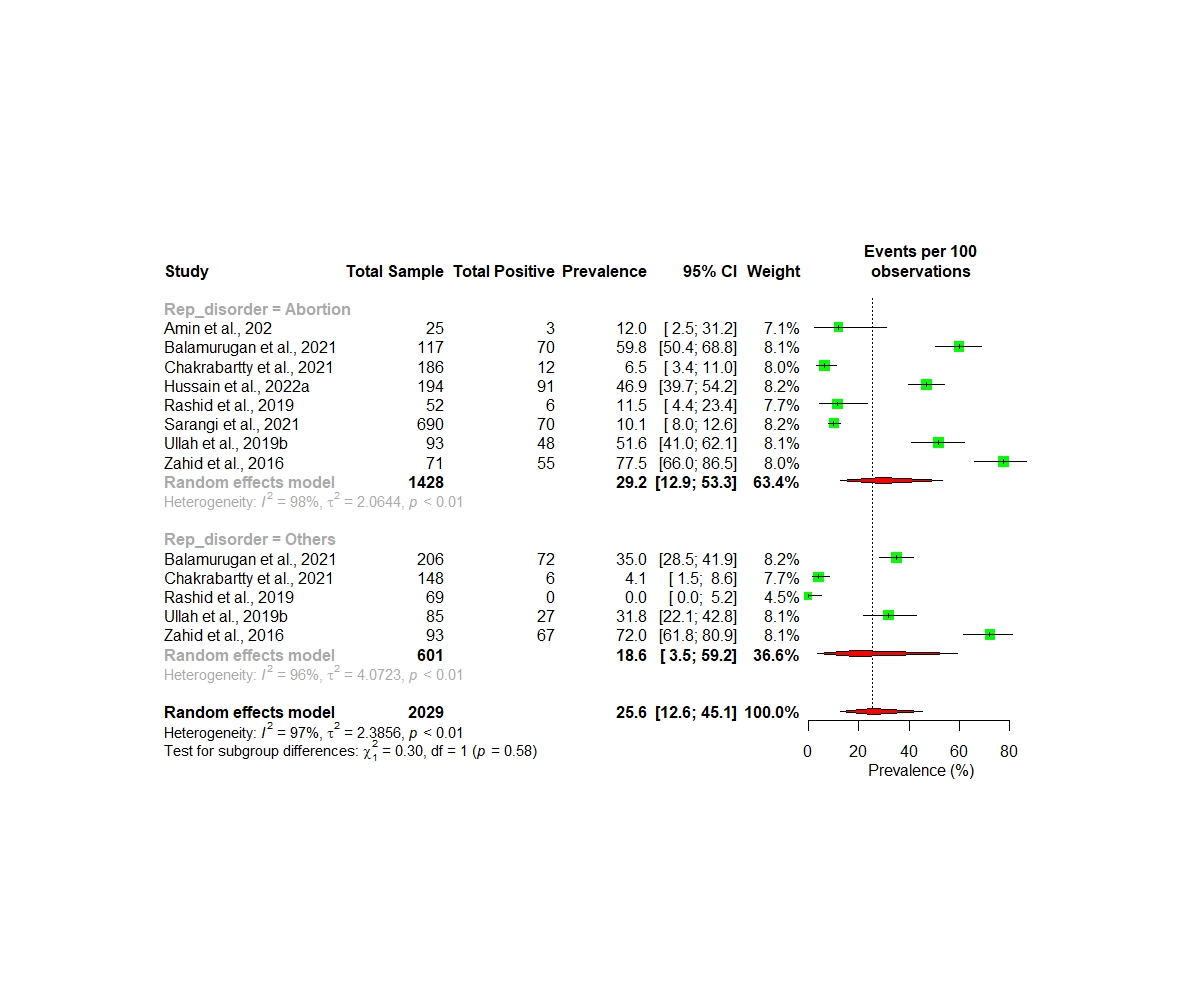


#### Ruminant history of abortion


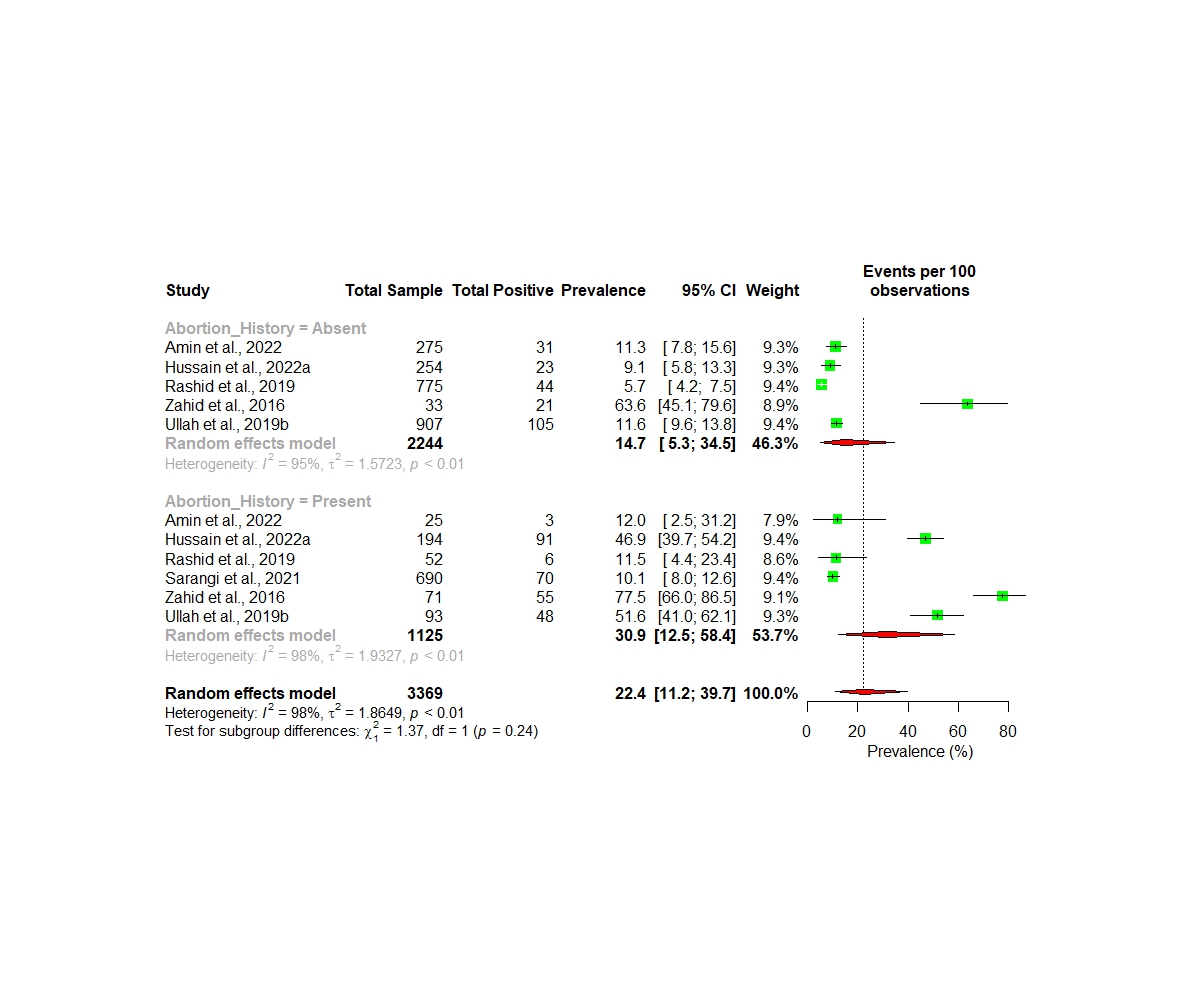


### Large ruminant level seroprevalence and associated risk factors

#### Large ruminant country


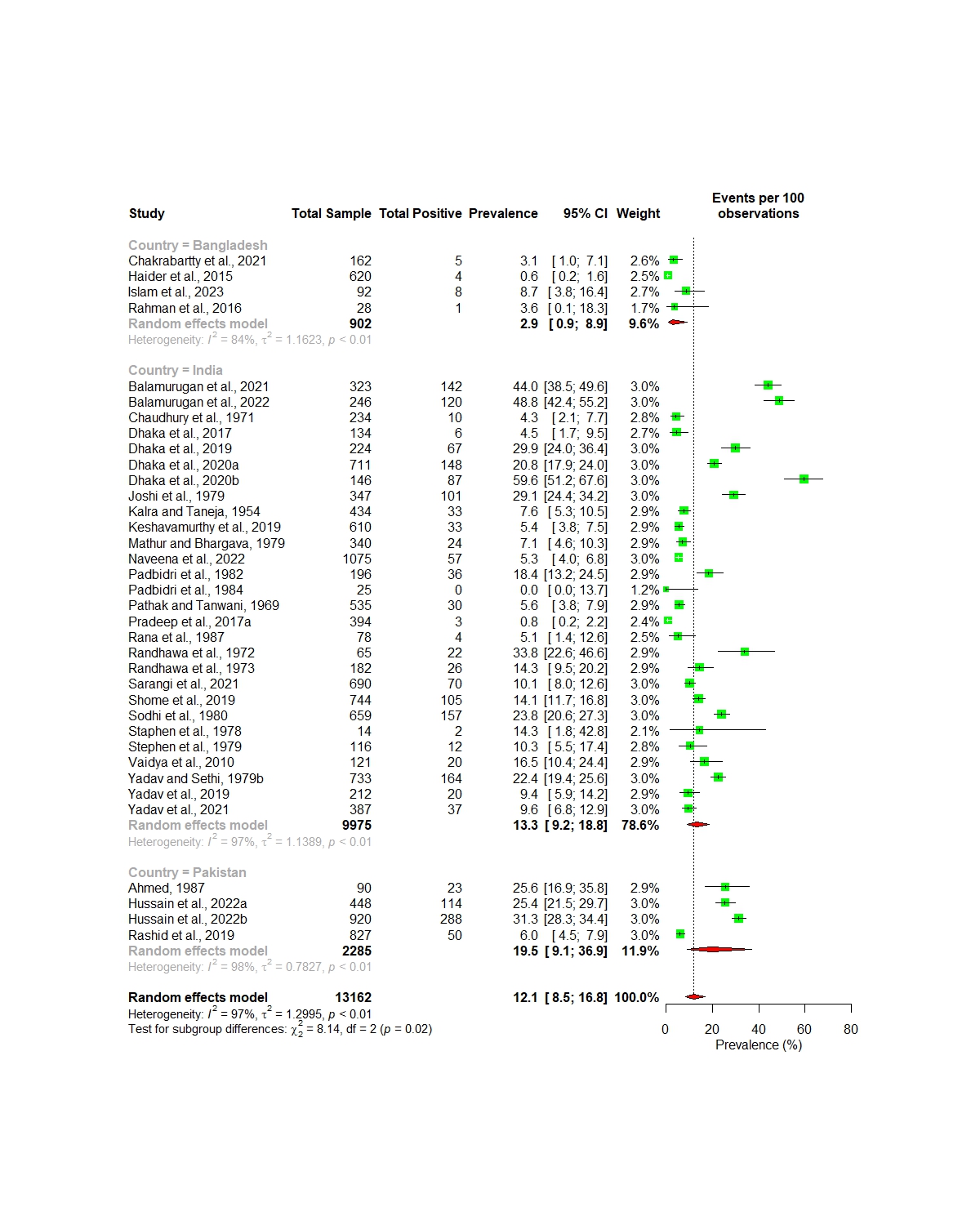


#### Large ruminant age


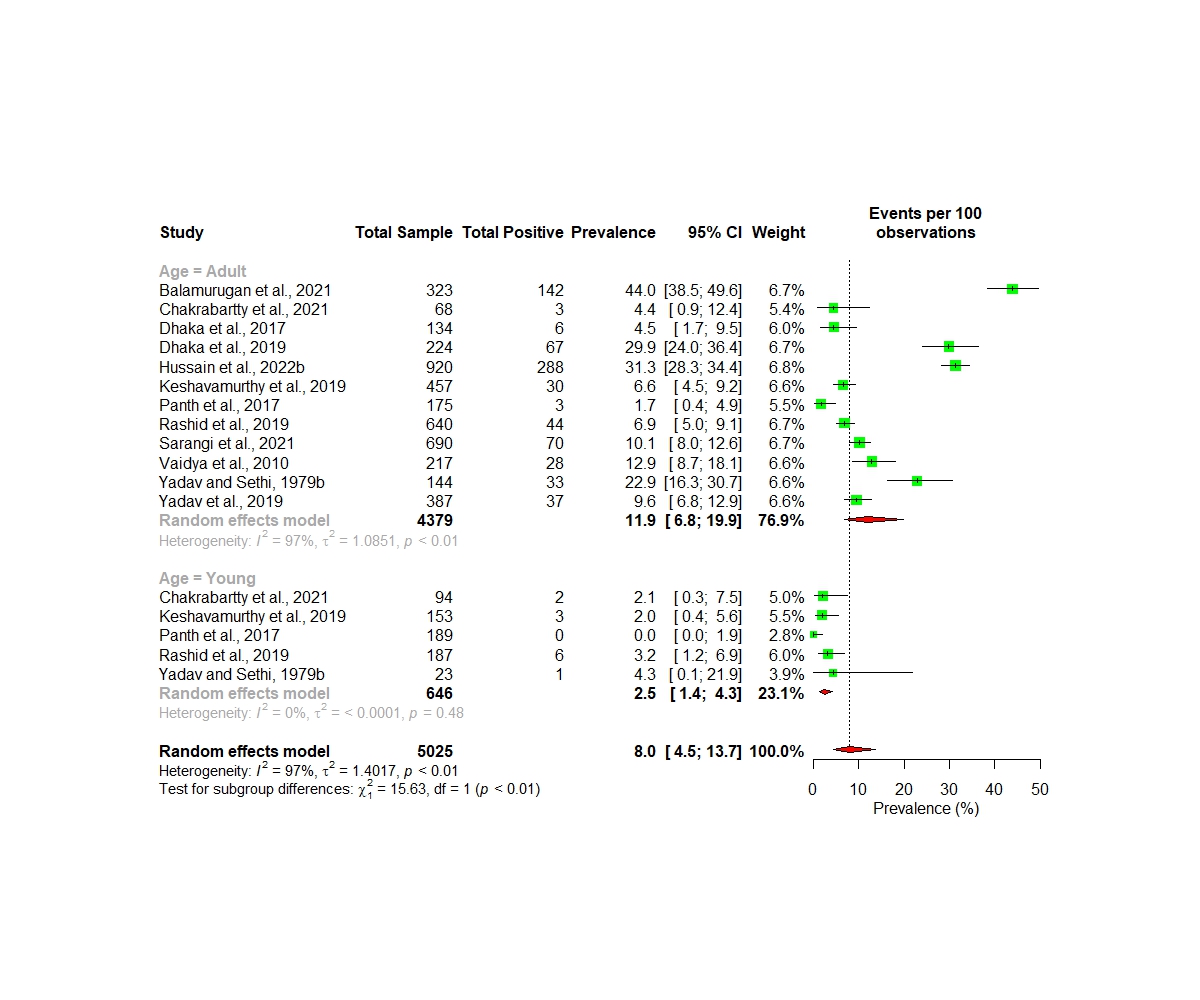


#### Large ruminant sex


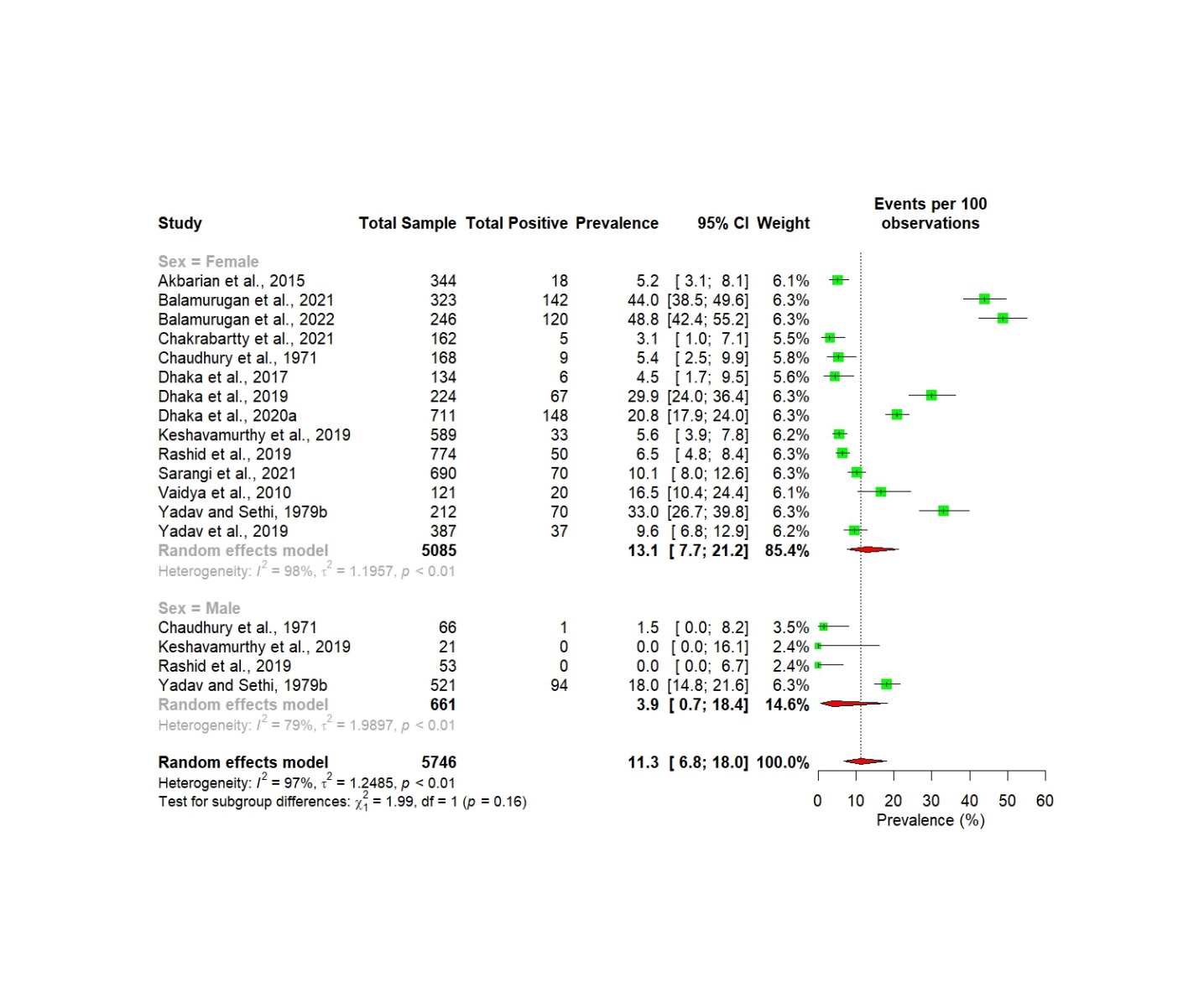


#### Large Ruminant Breed


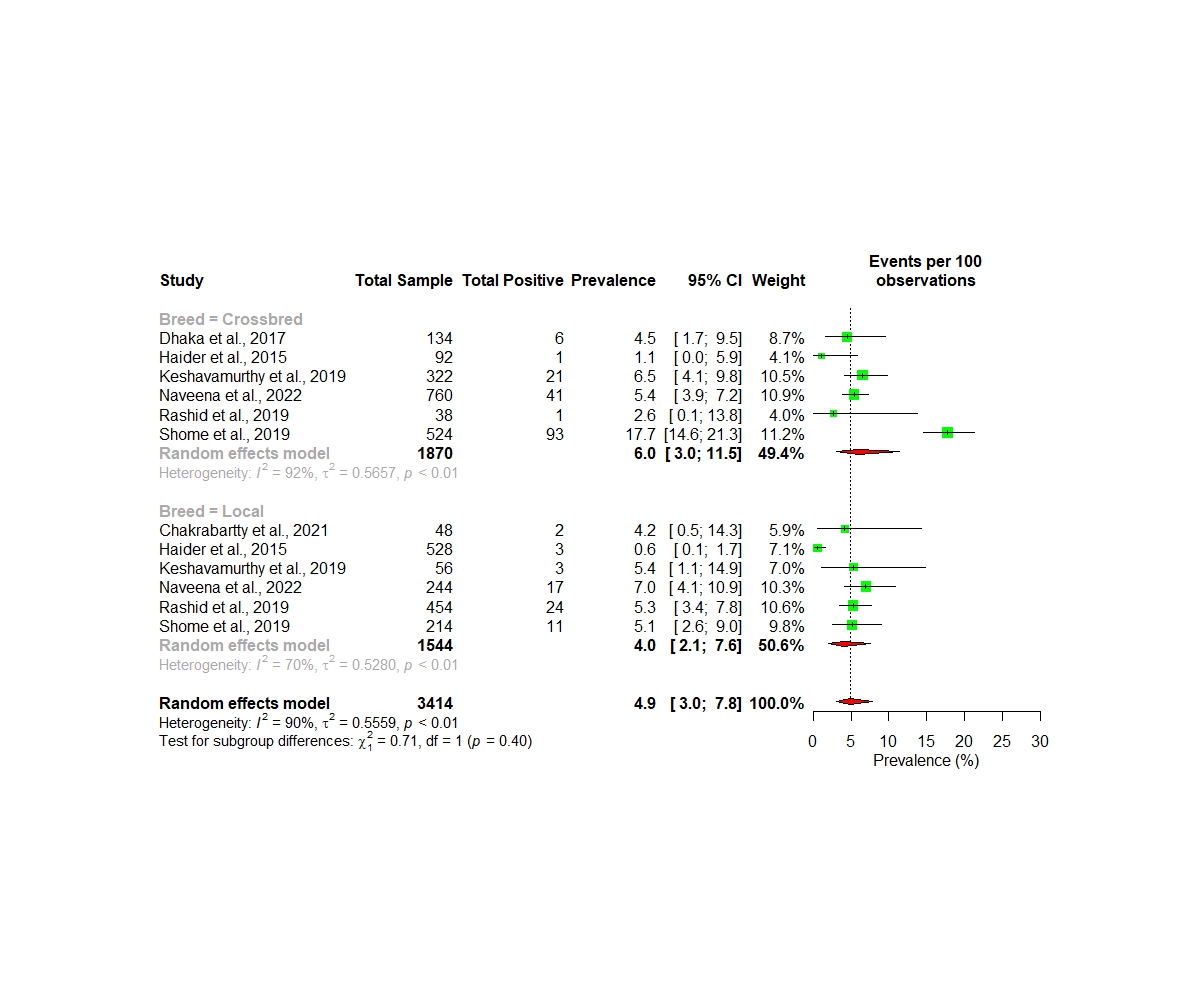


#### Large ruminant grazing


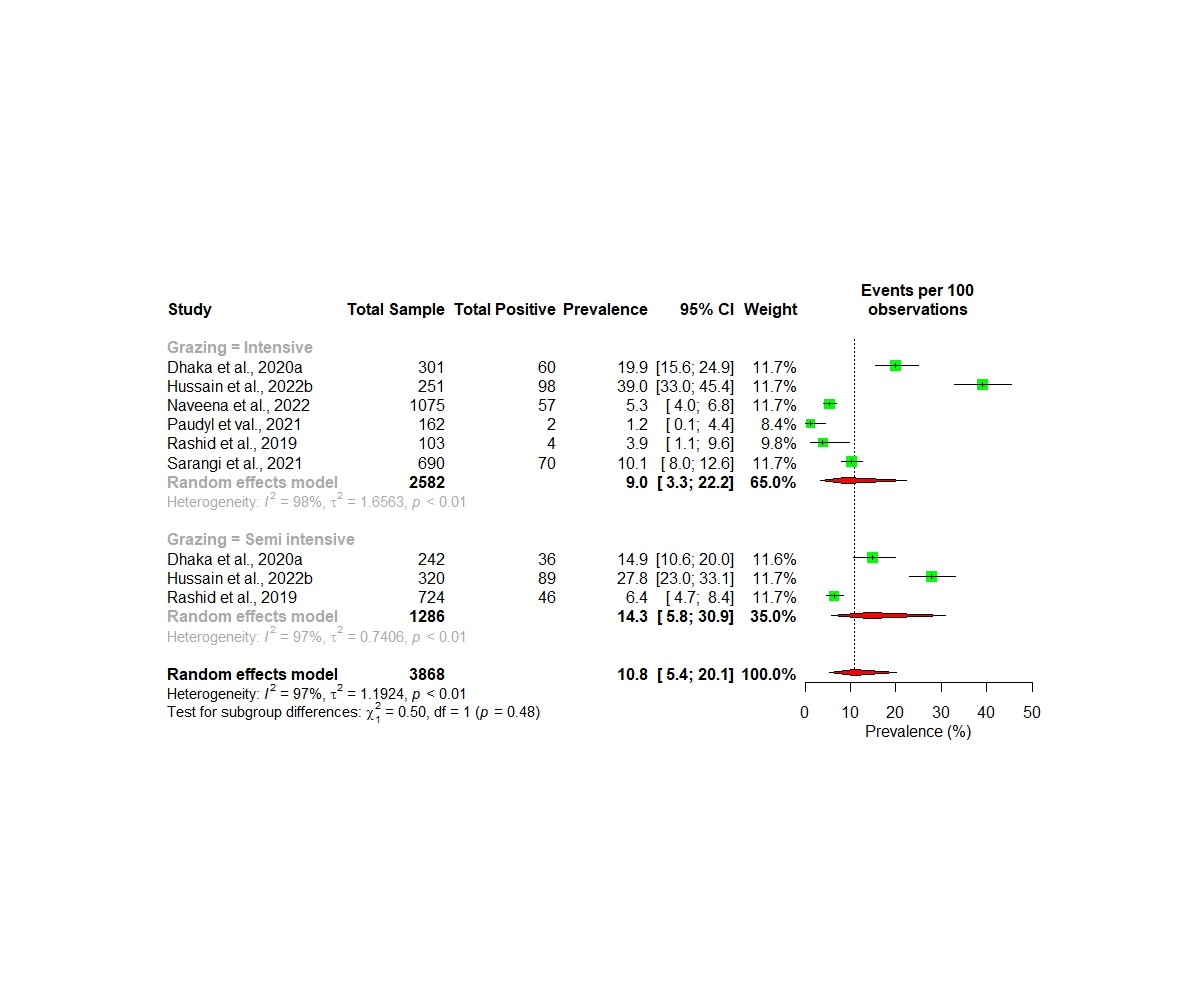


#### Large ruminant biosafety


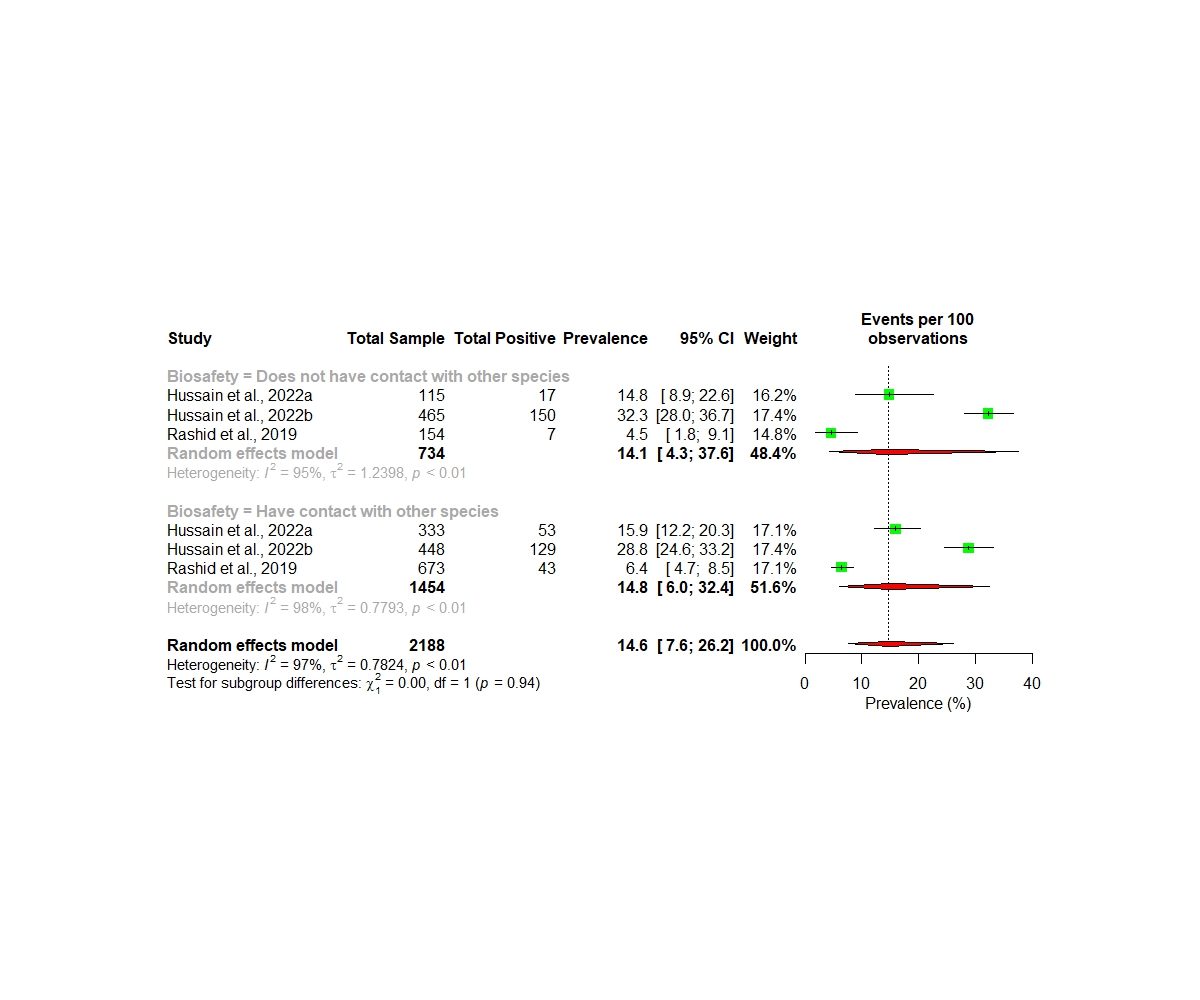


#### Large ruminant tick infestation


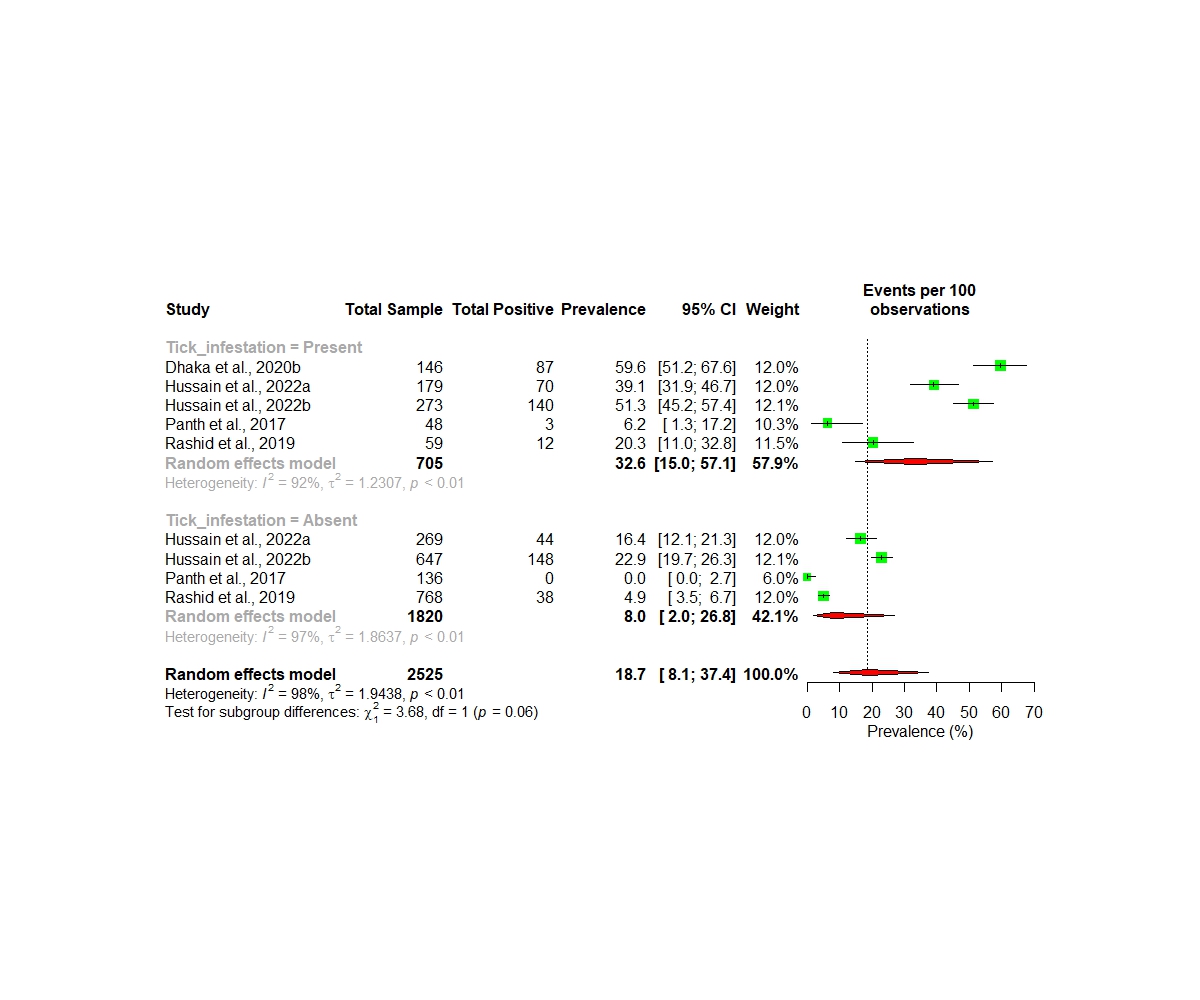


#### Large ruminant history of reproductive disorder


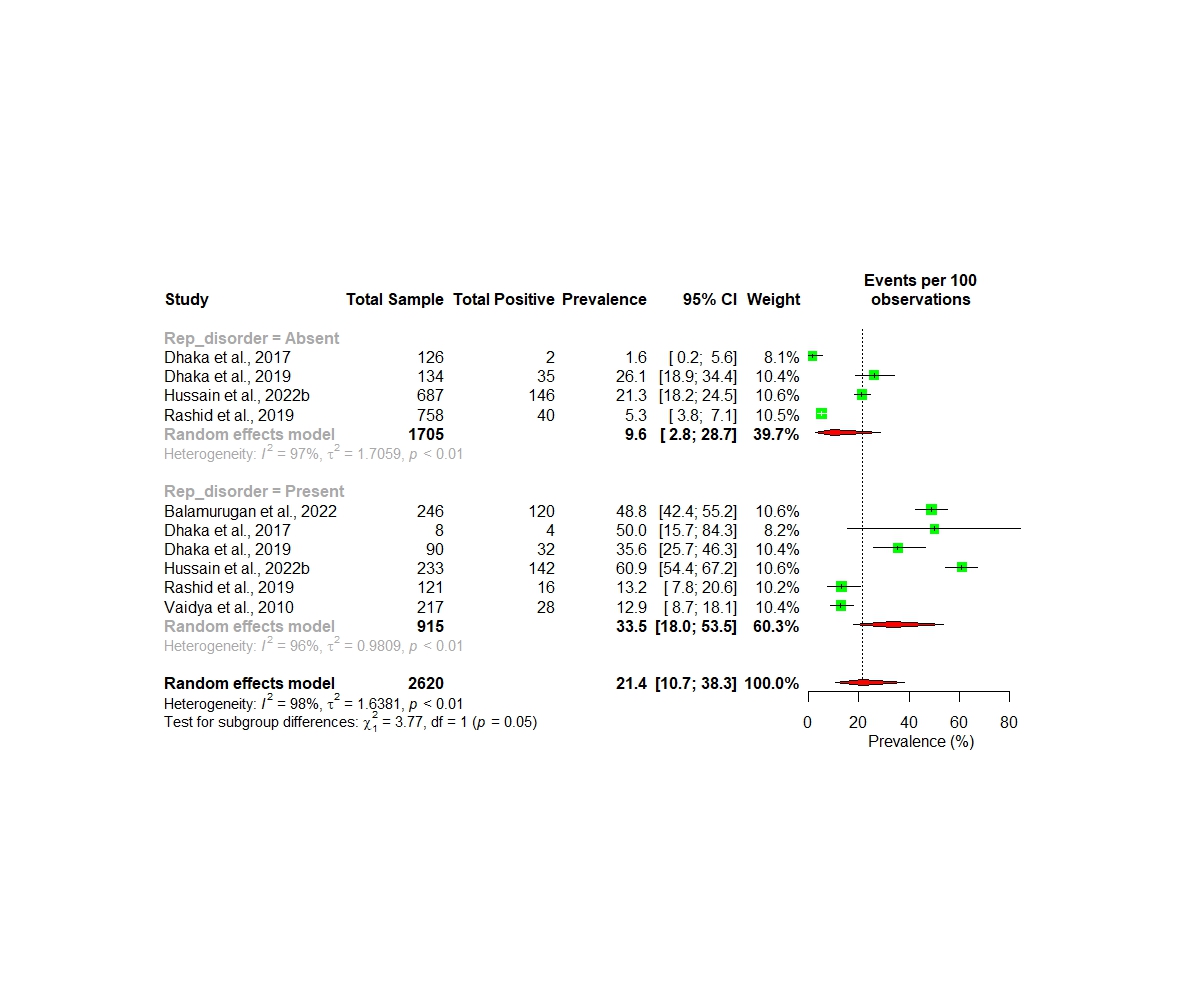


#### LR_type of reproductive disorder


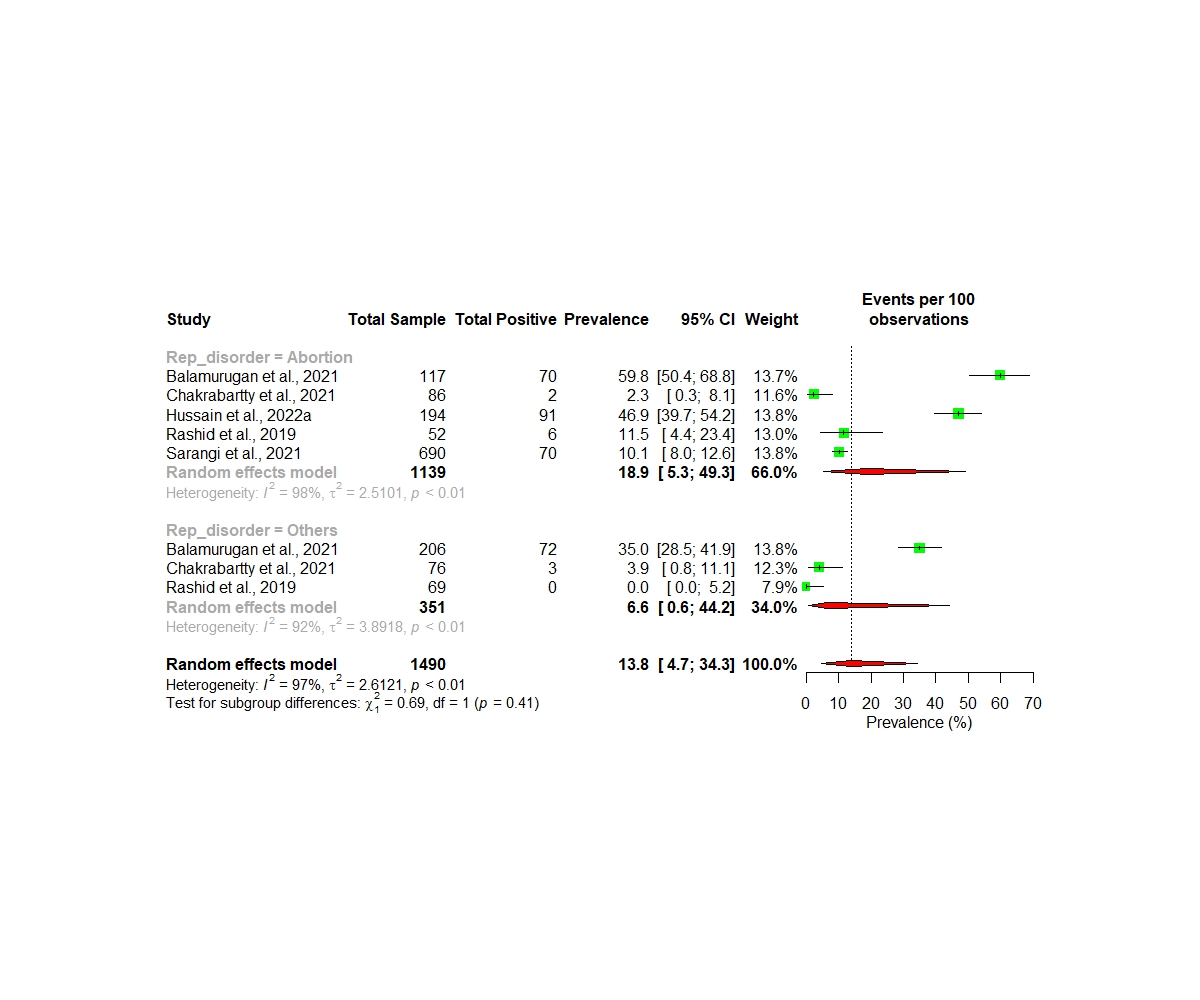


### Small ruminant level seroprevalence and associated risk factors

#### Small ruminant country


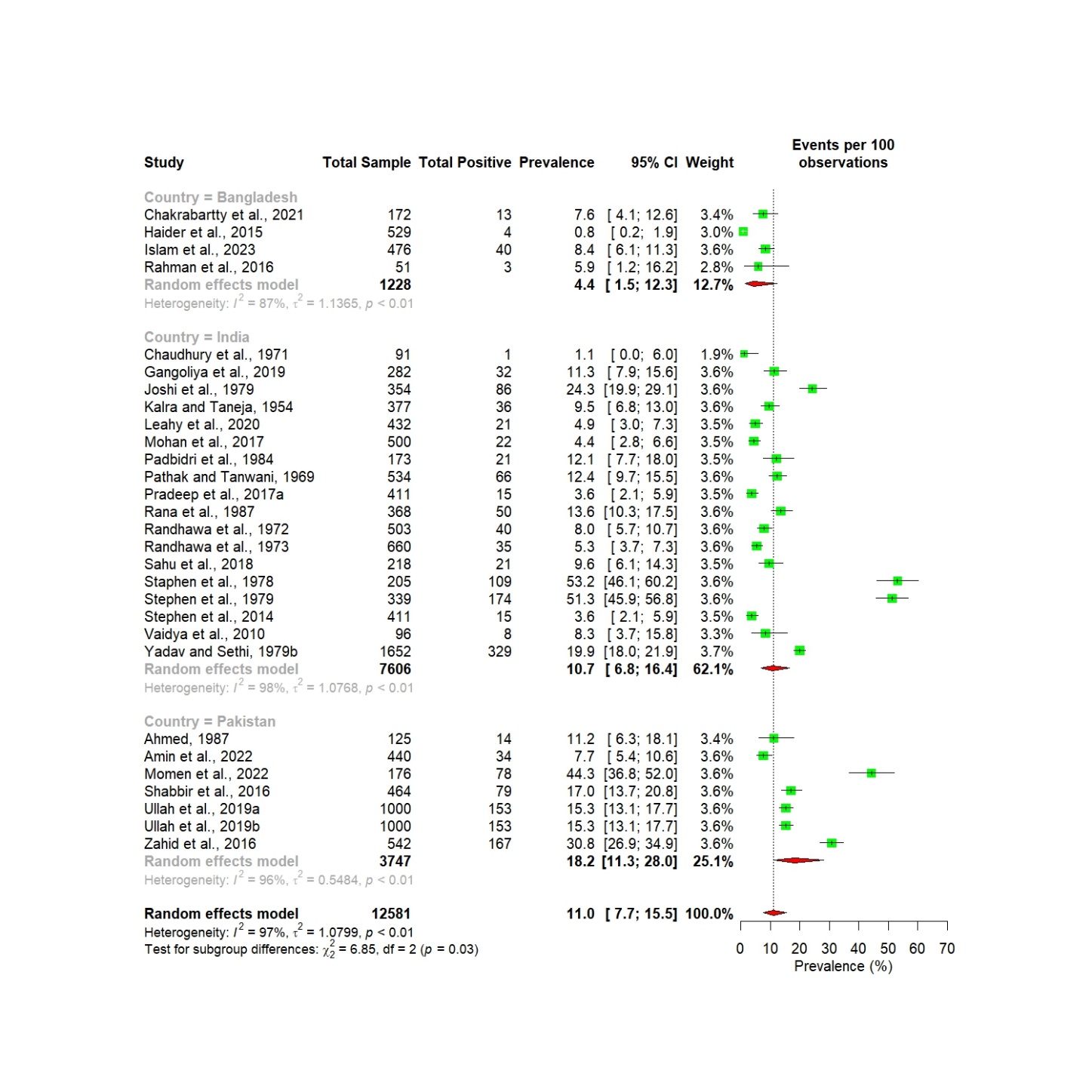


#### Small ruminant age


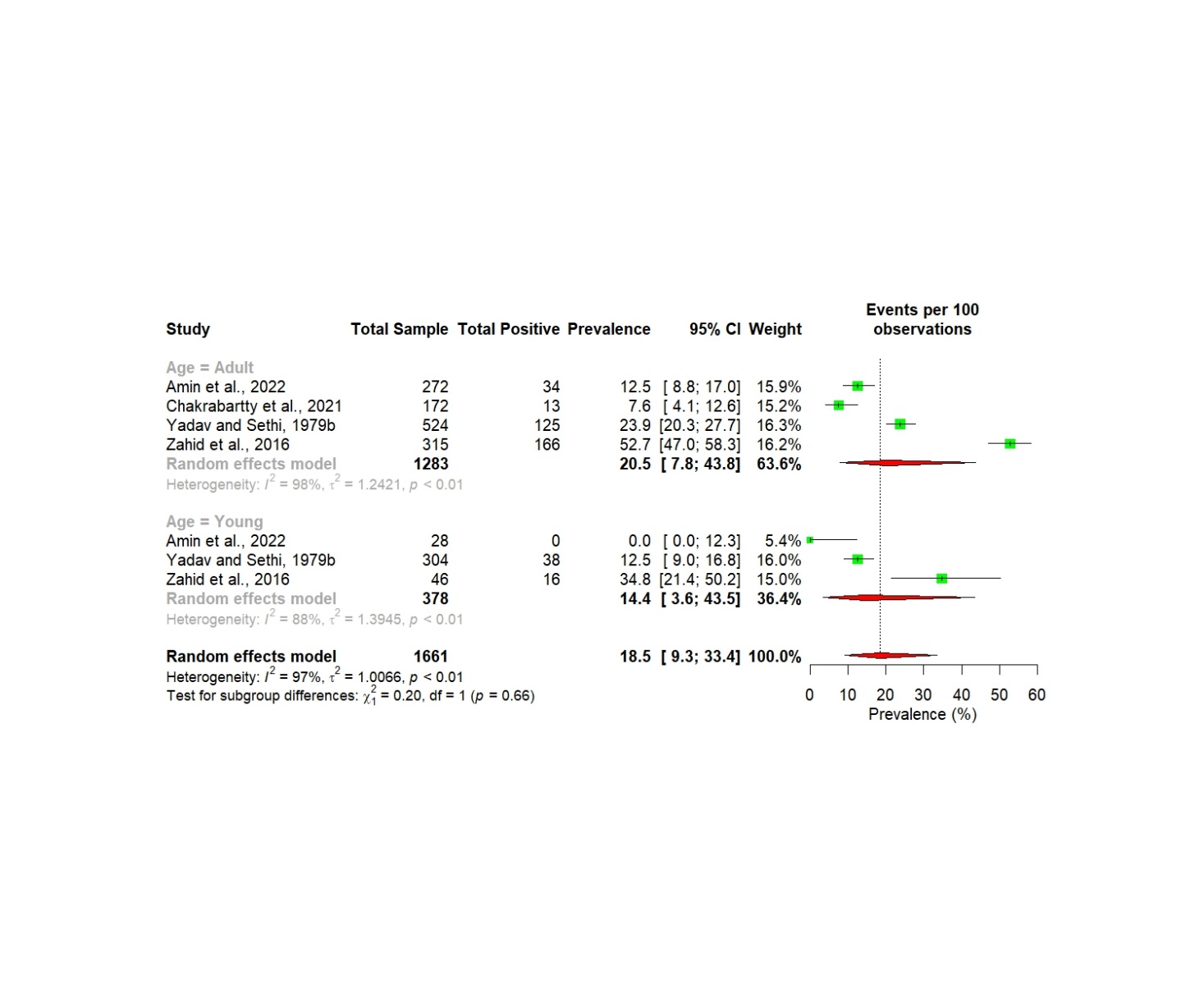


#### Small ruminant sex


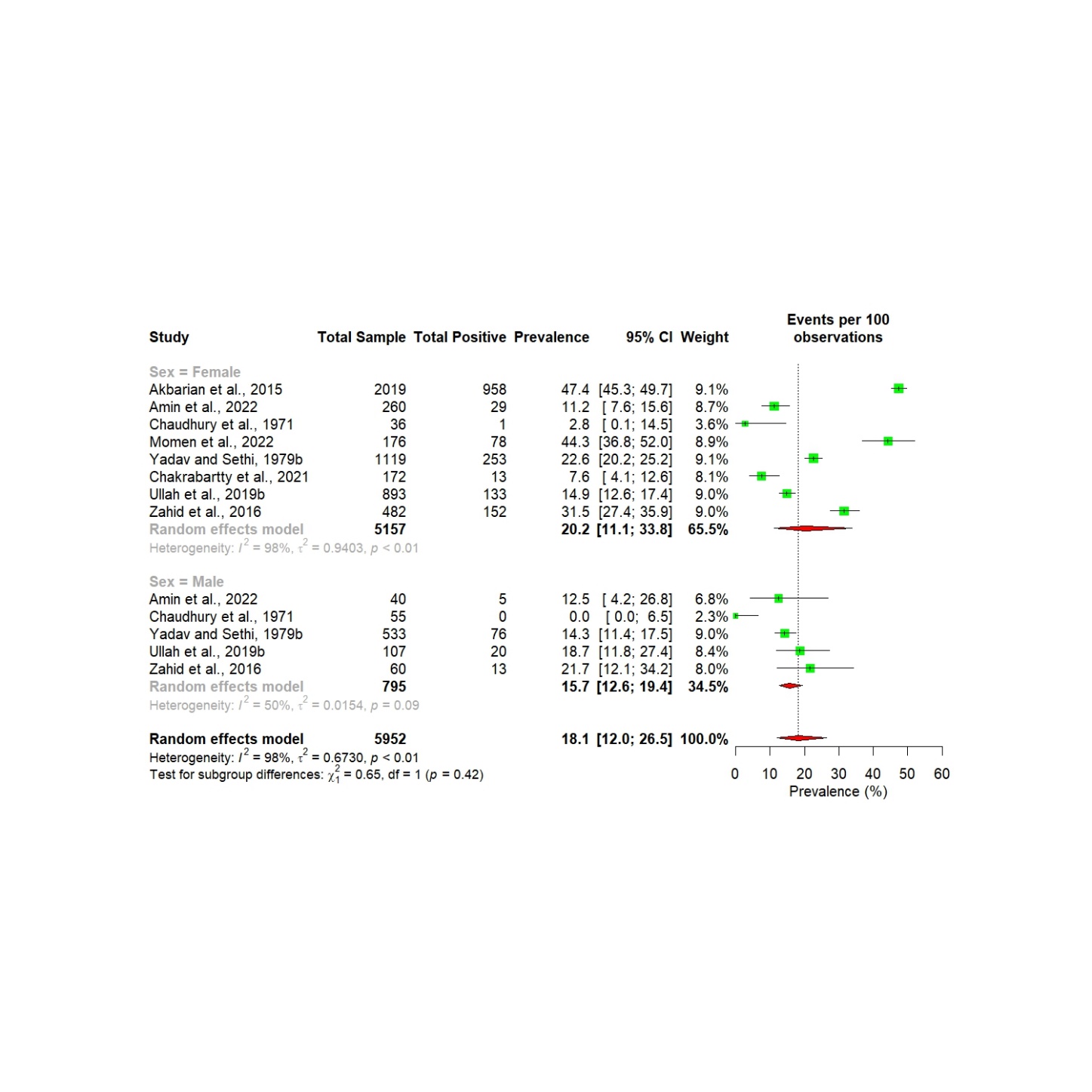


#### Small ruminant grazing


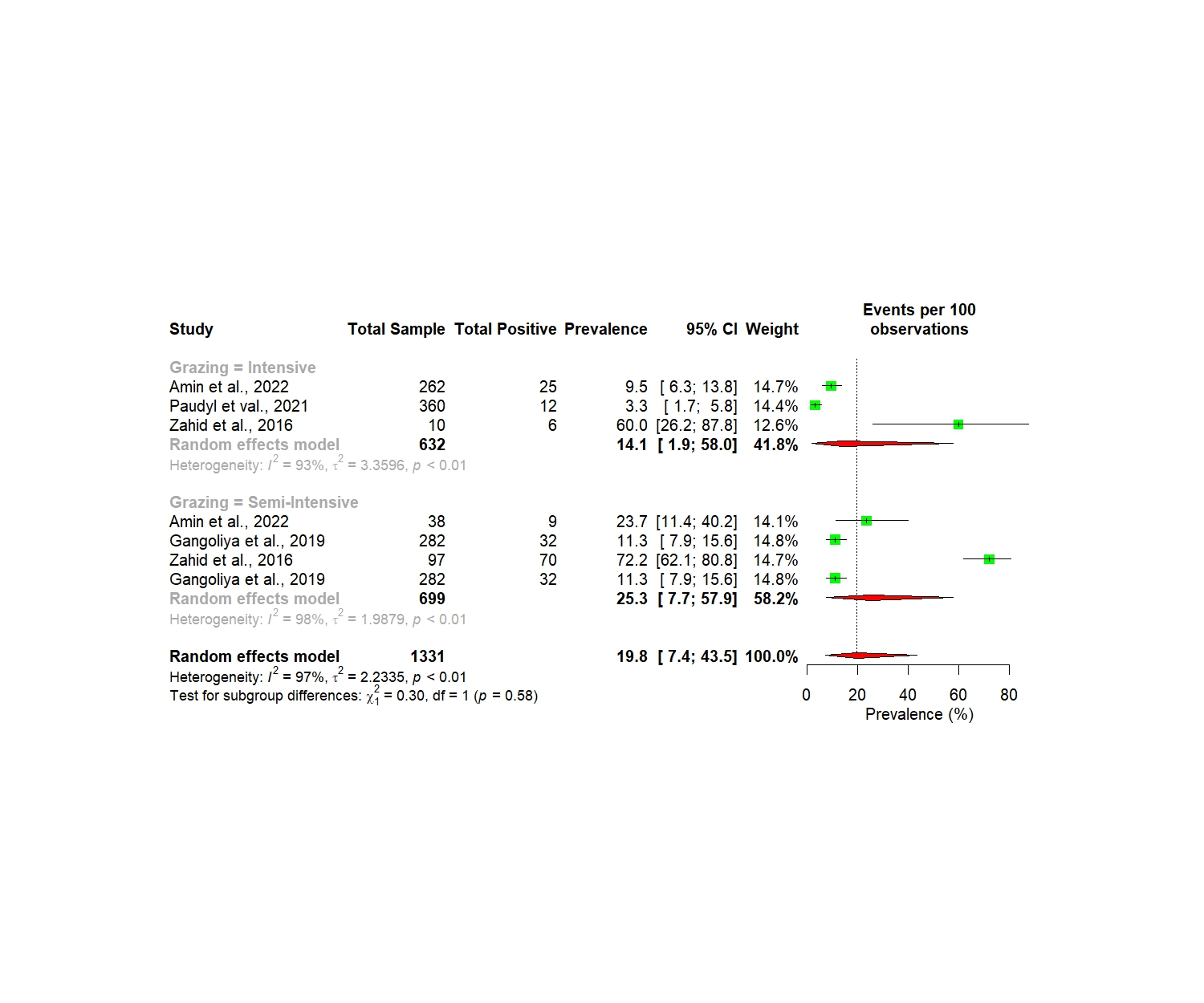


#### Small ruminant_bisafety


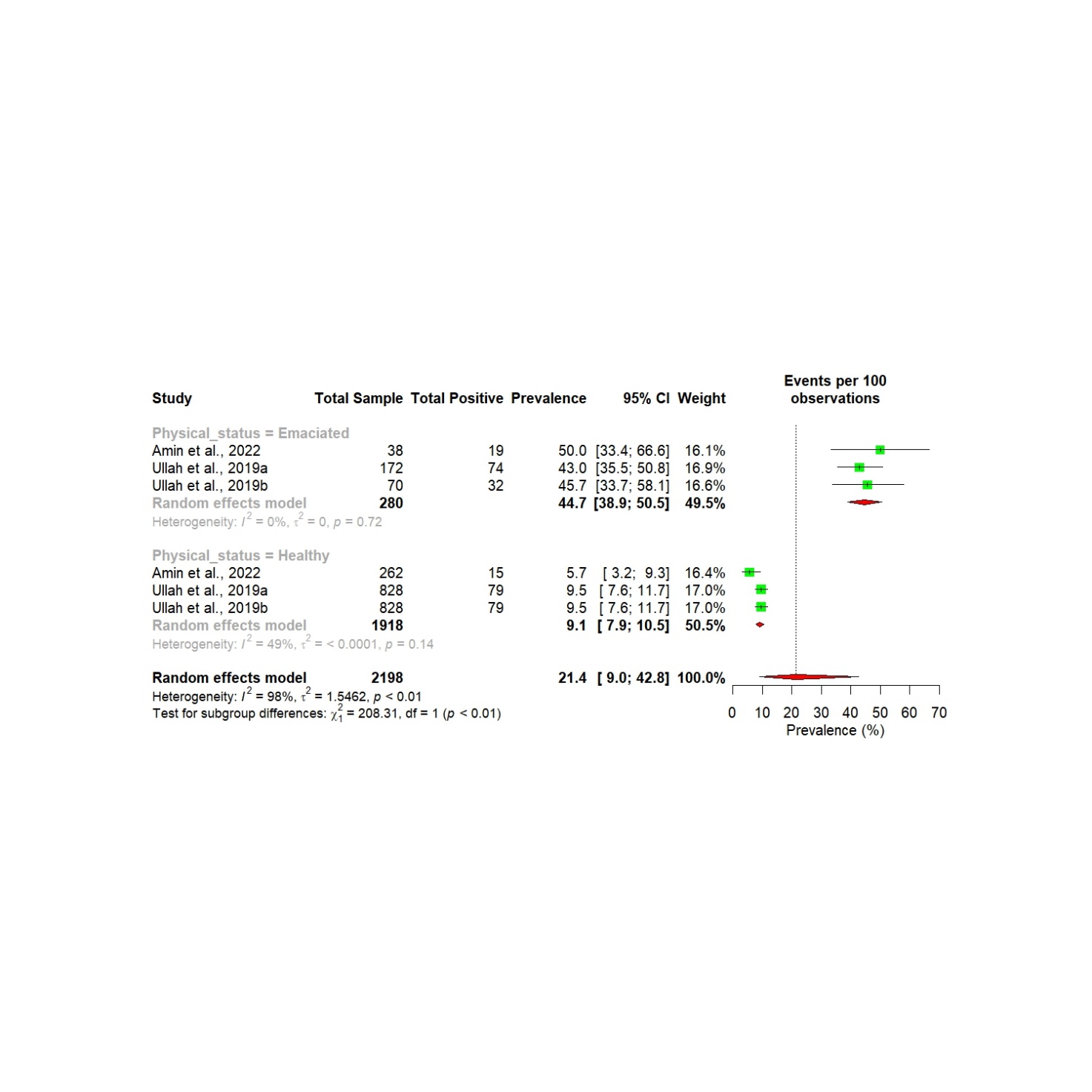


#### Small ruminant Tick infestation


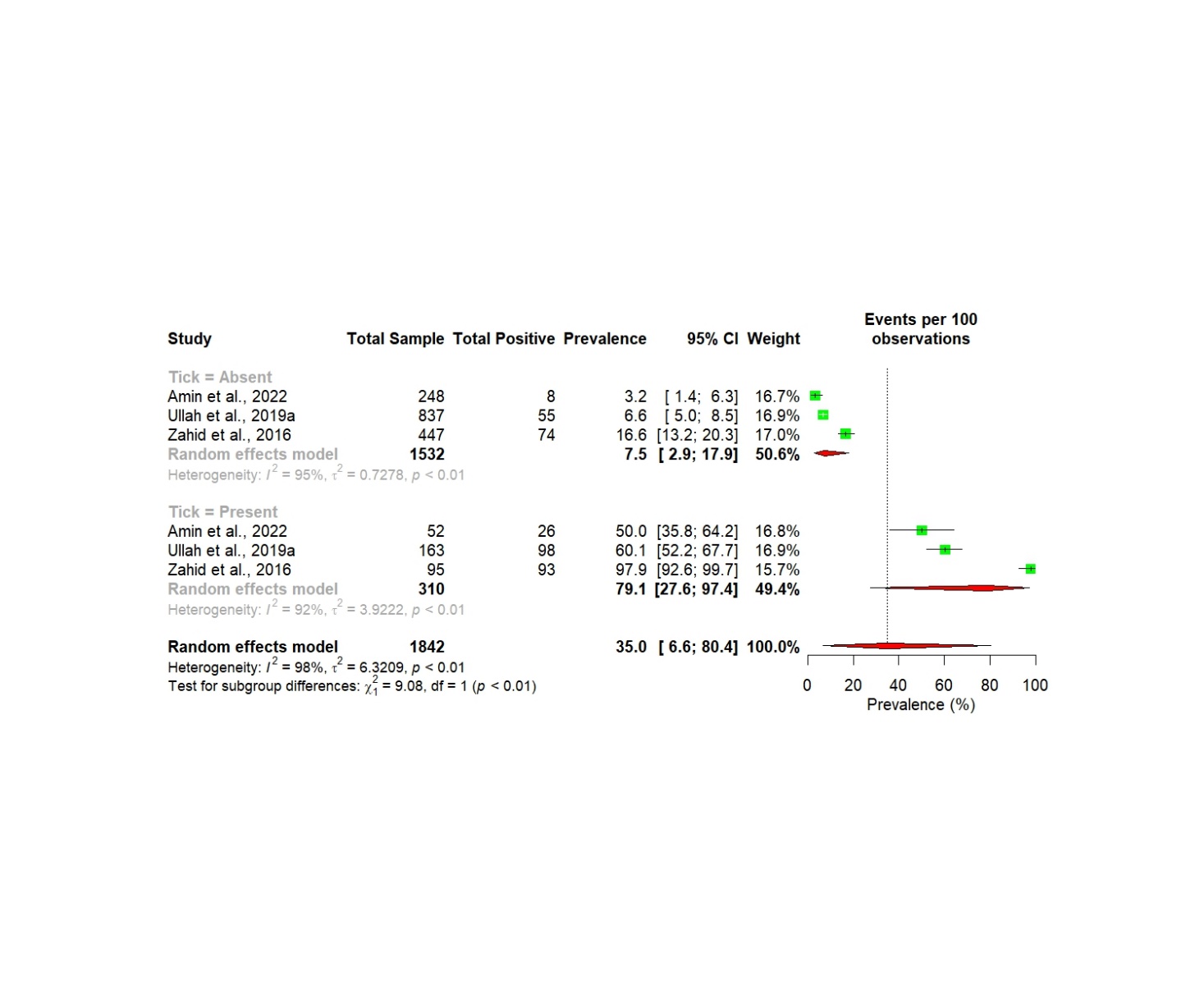


#### Small ruminant type of reproductive disorder


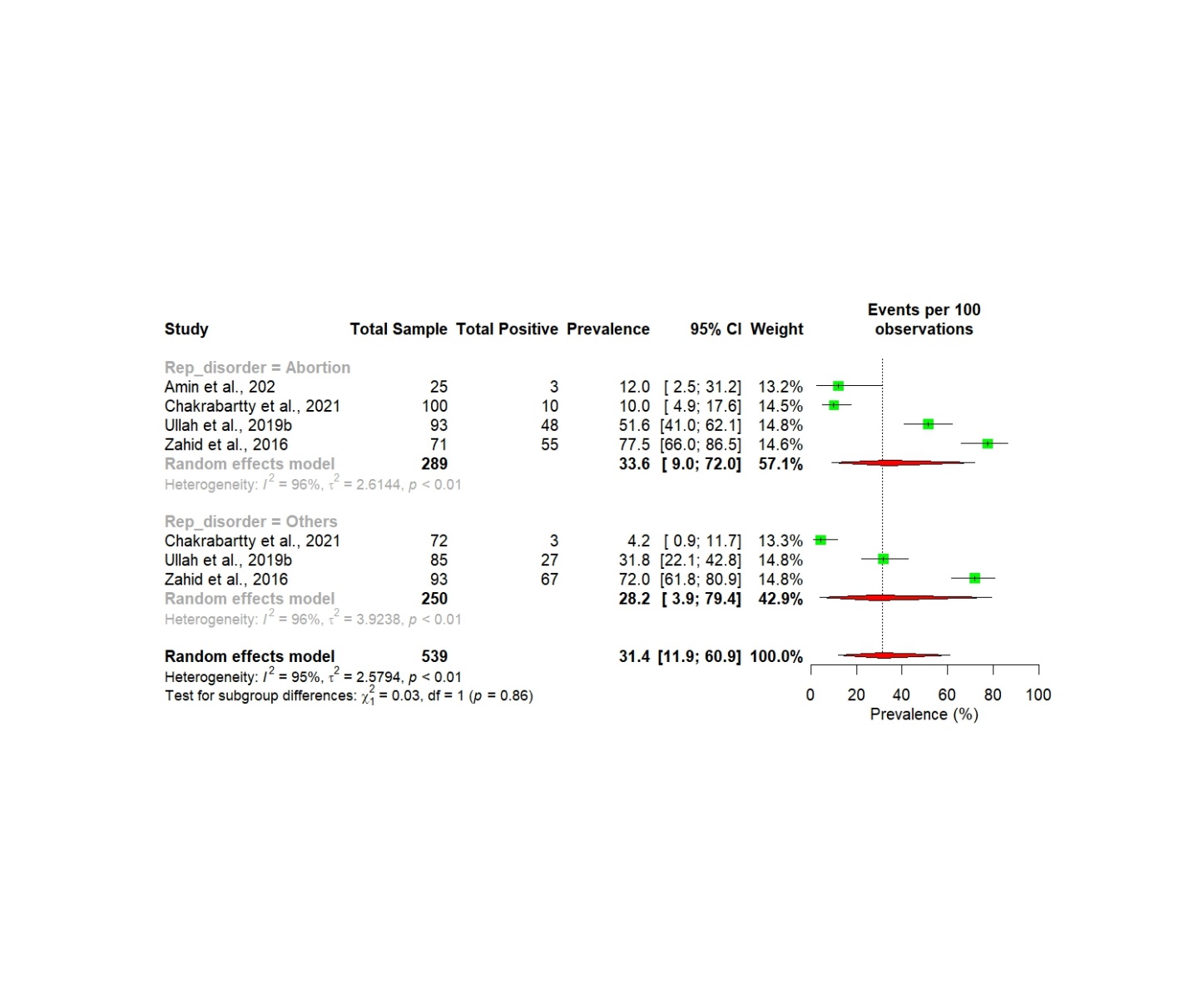


#### Small ruminant History of abortion


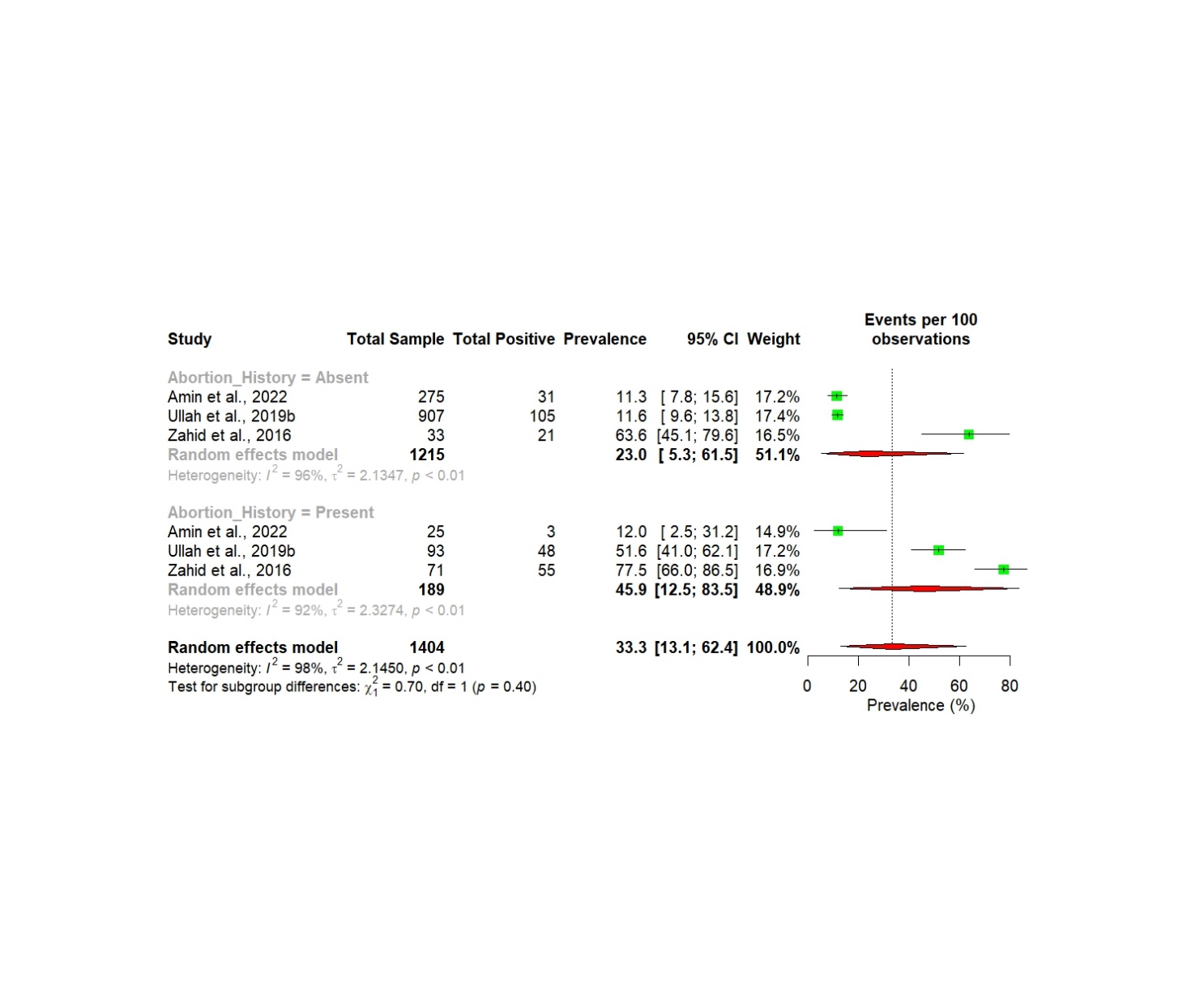


### Individual species level seroprevalence and associated risk factors

#### Cattle country


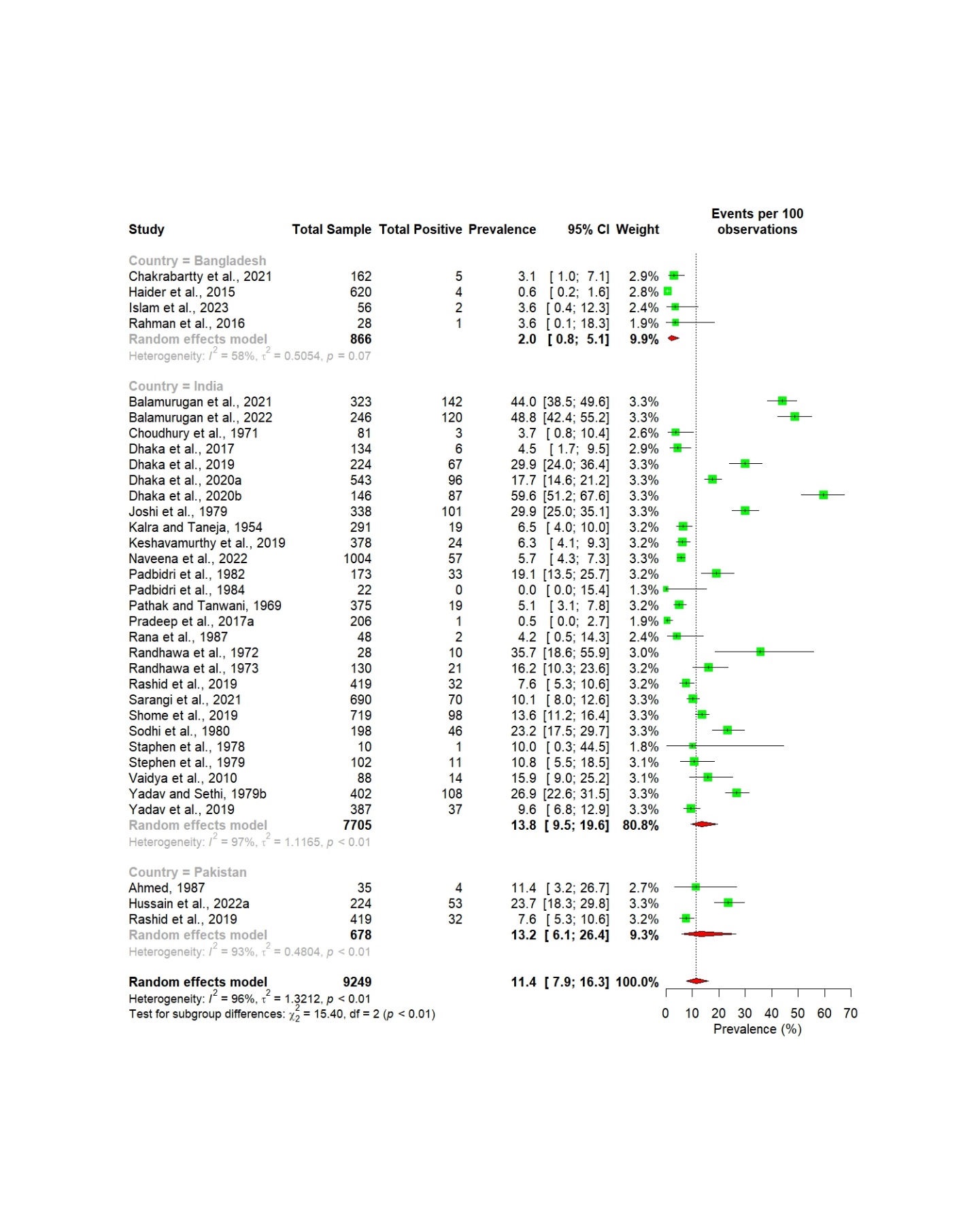


#### Cattle age


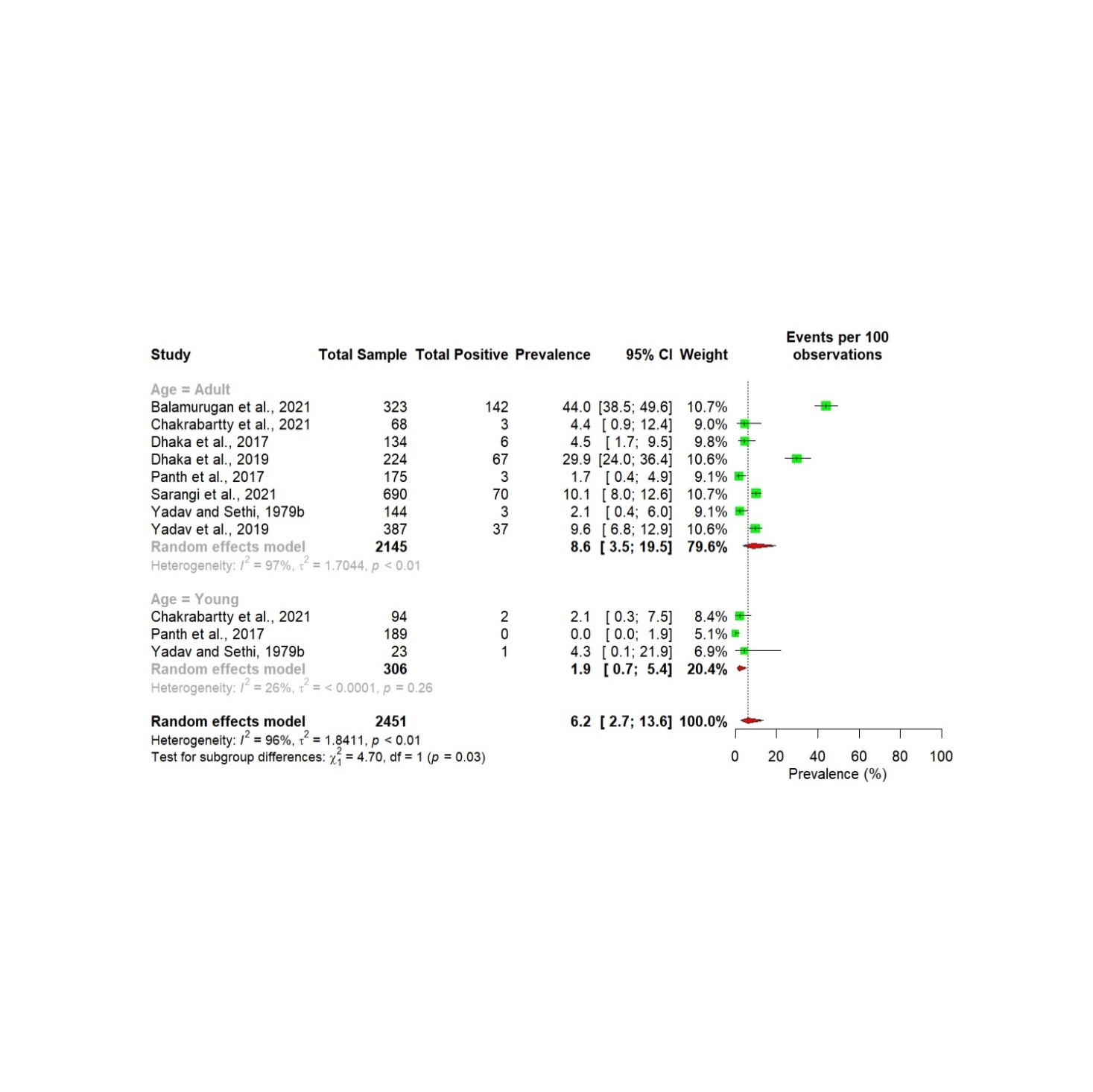


#### Cattle breed


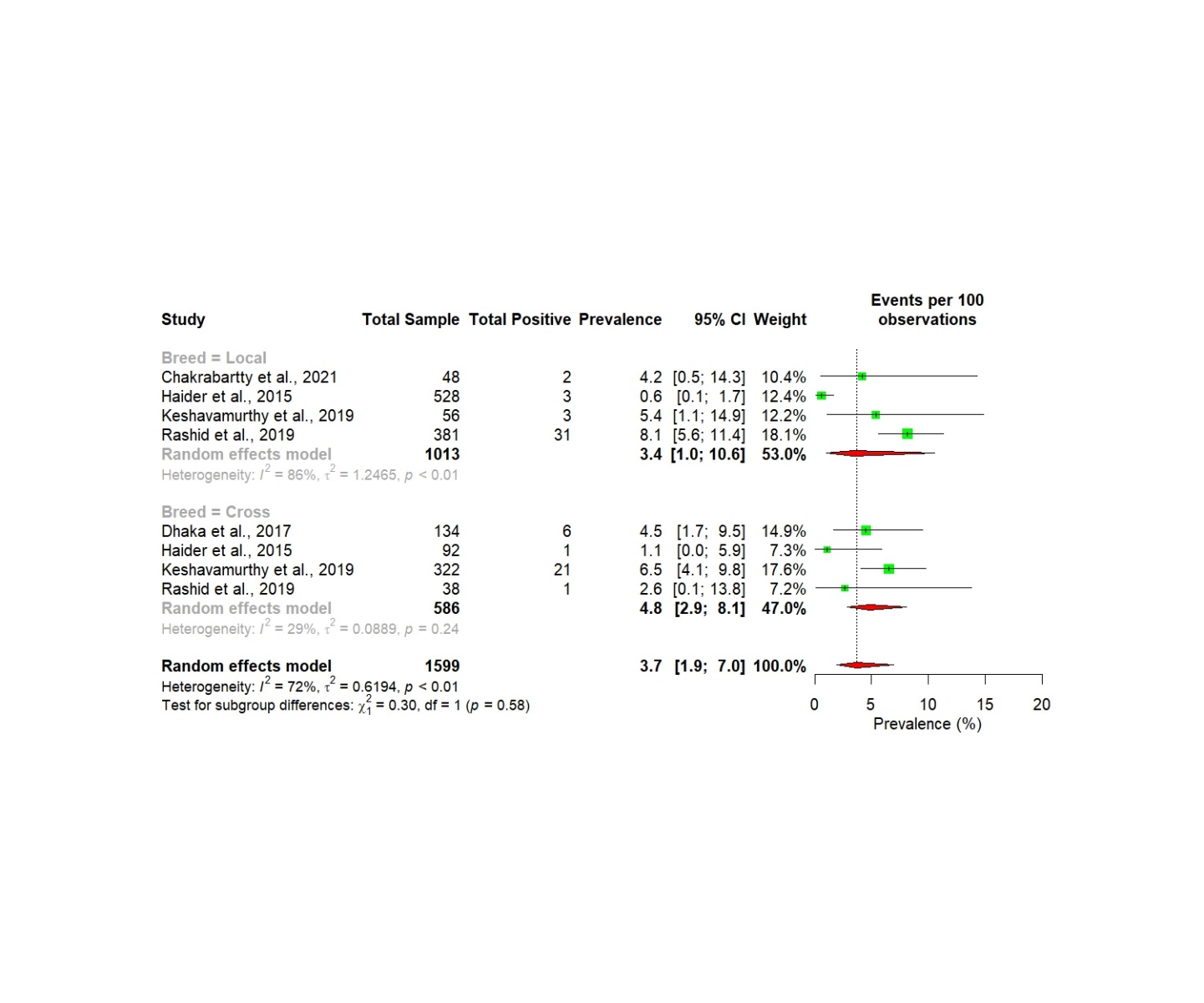


#### Sheep country


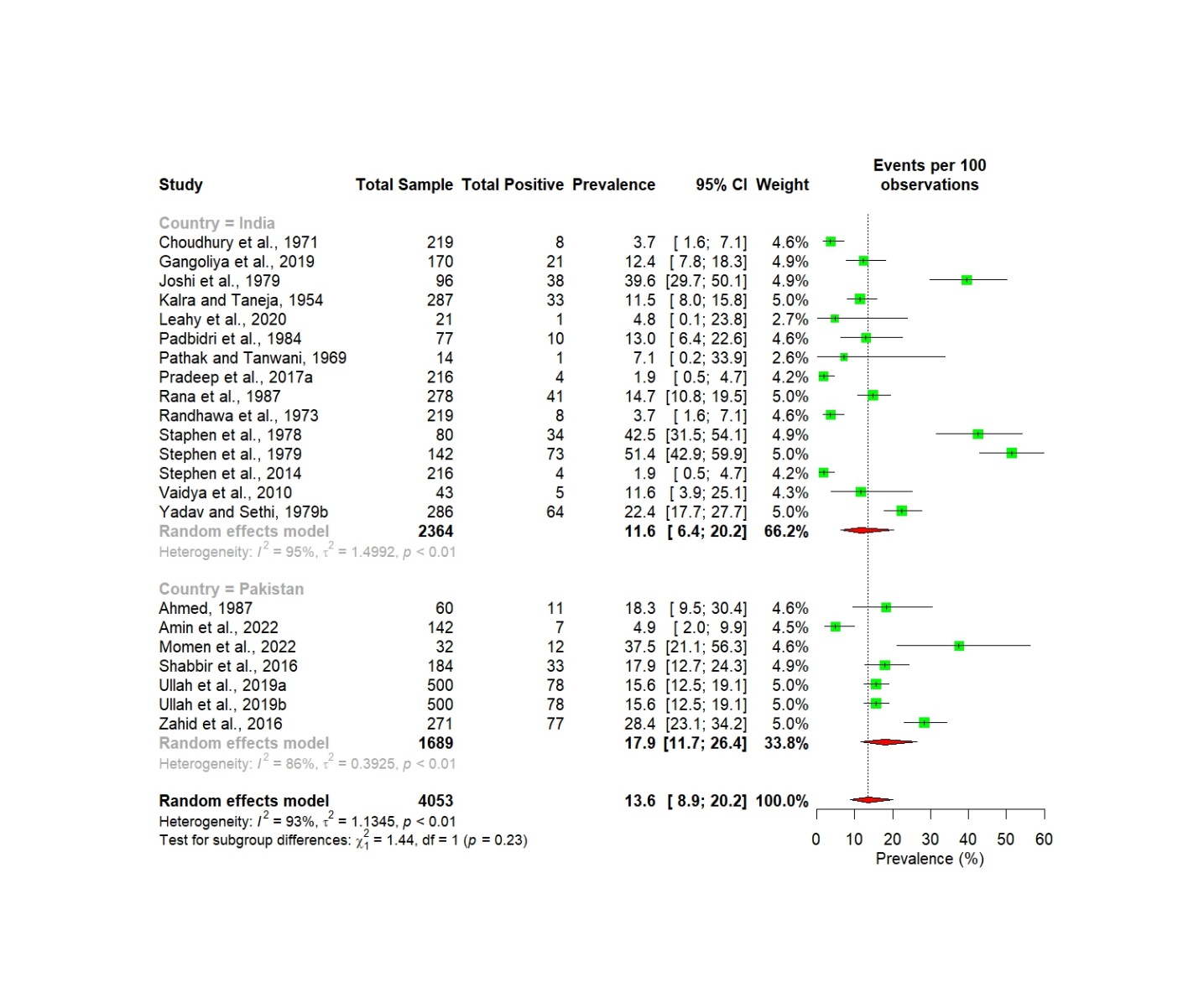


#### Goat_country


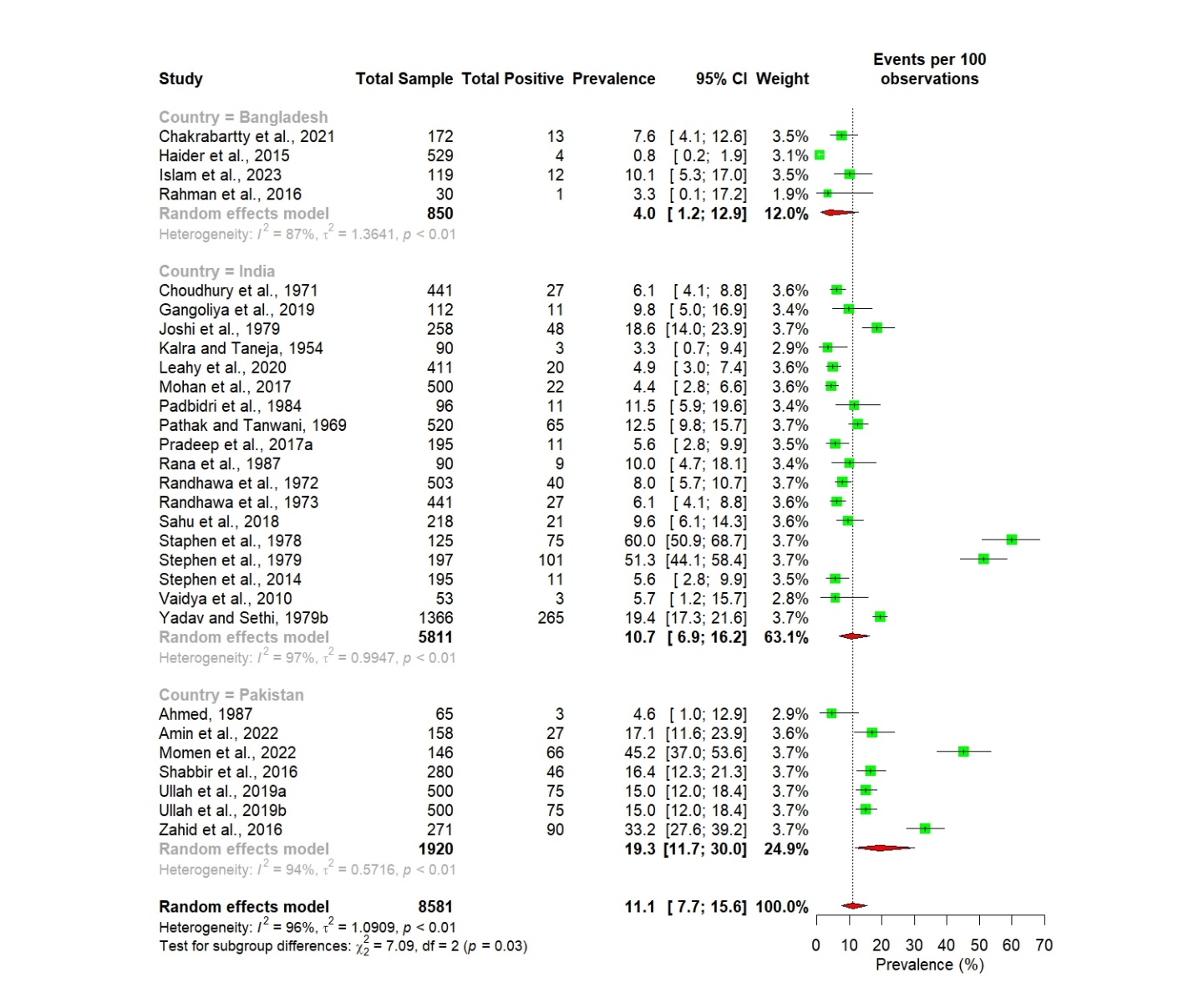


### Individual animal level carrier prevalence and associated risk factors

#### Individual carrier overall


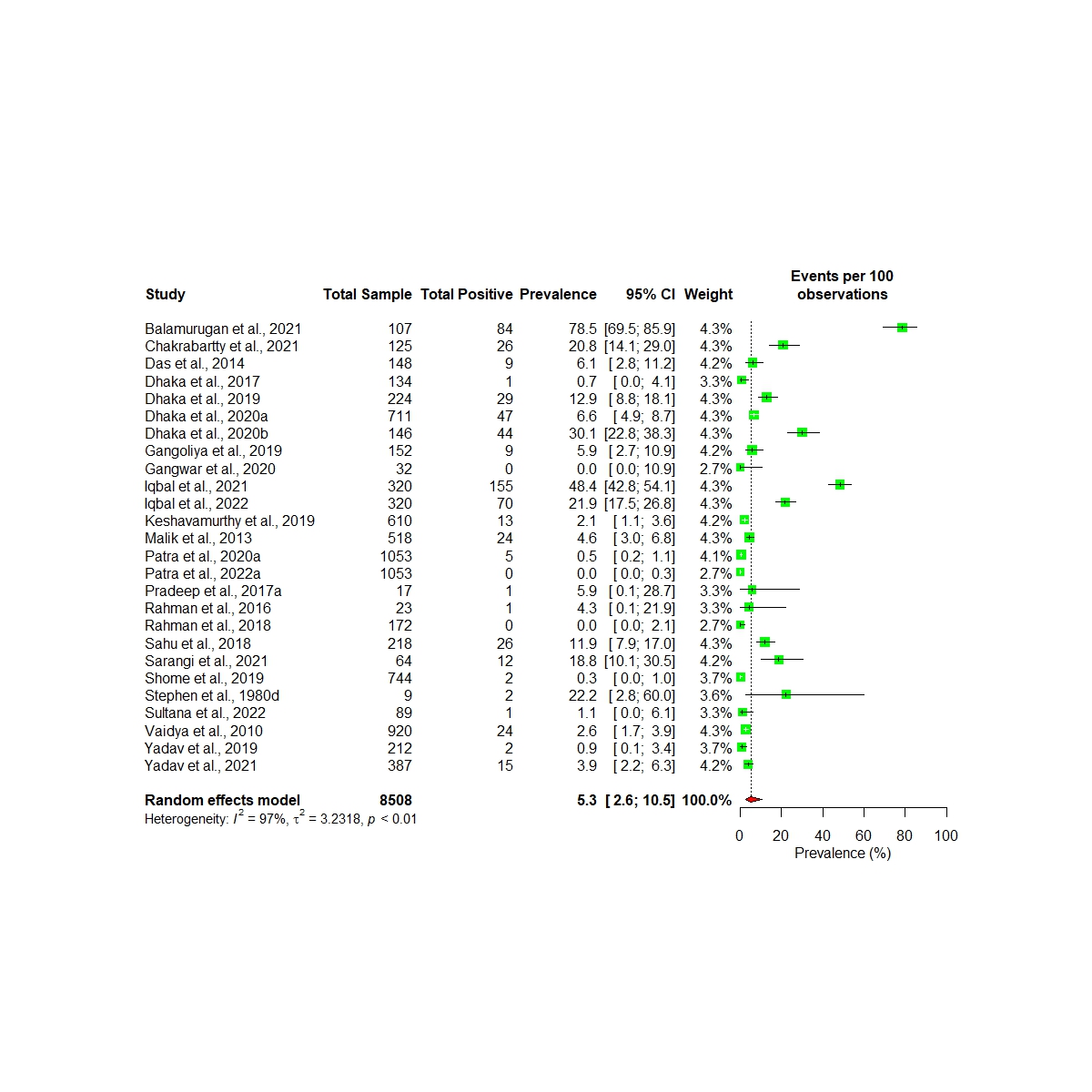


#### Individual carrier_LRSR


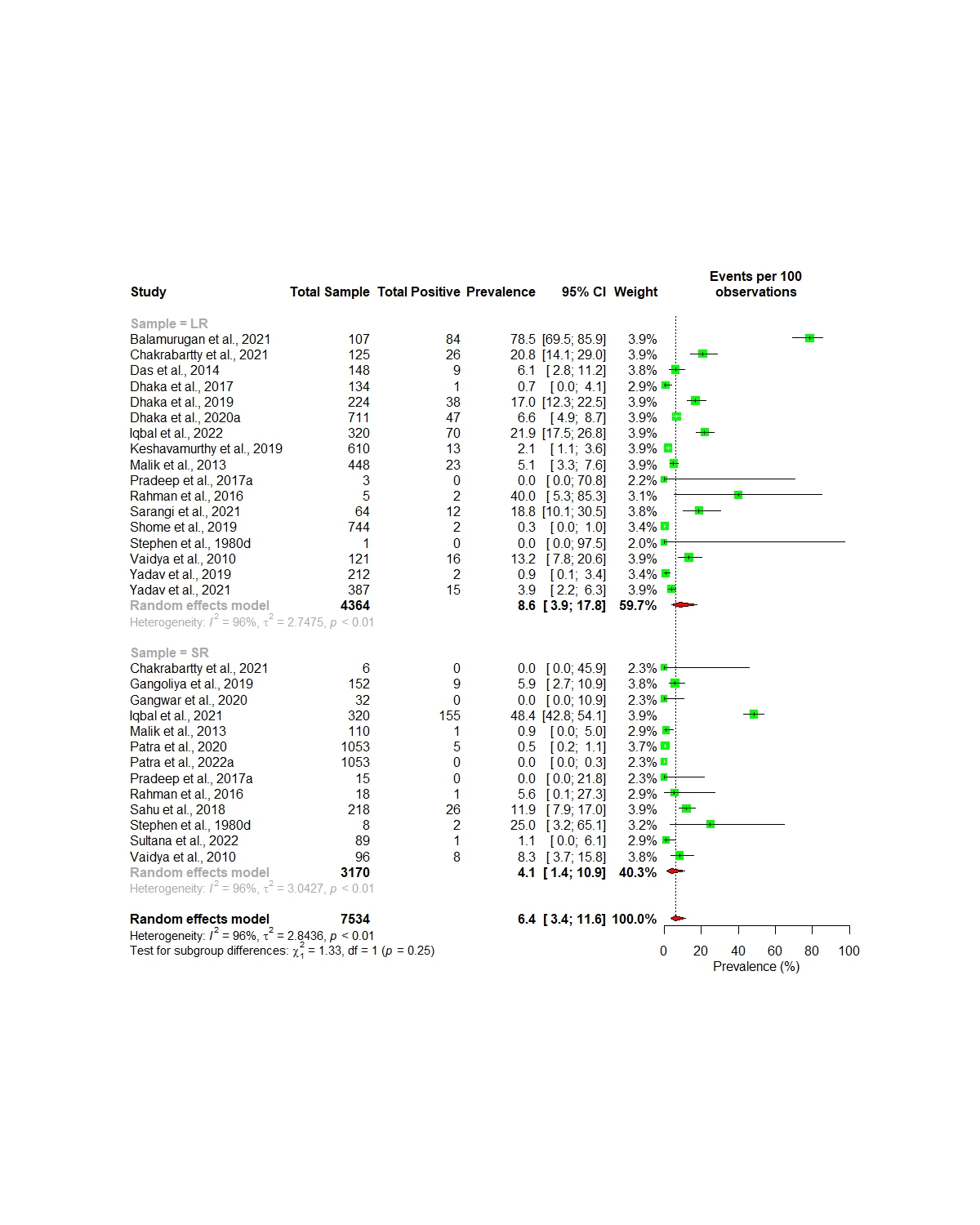


#### Individual carrier_ sample


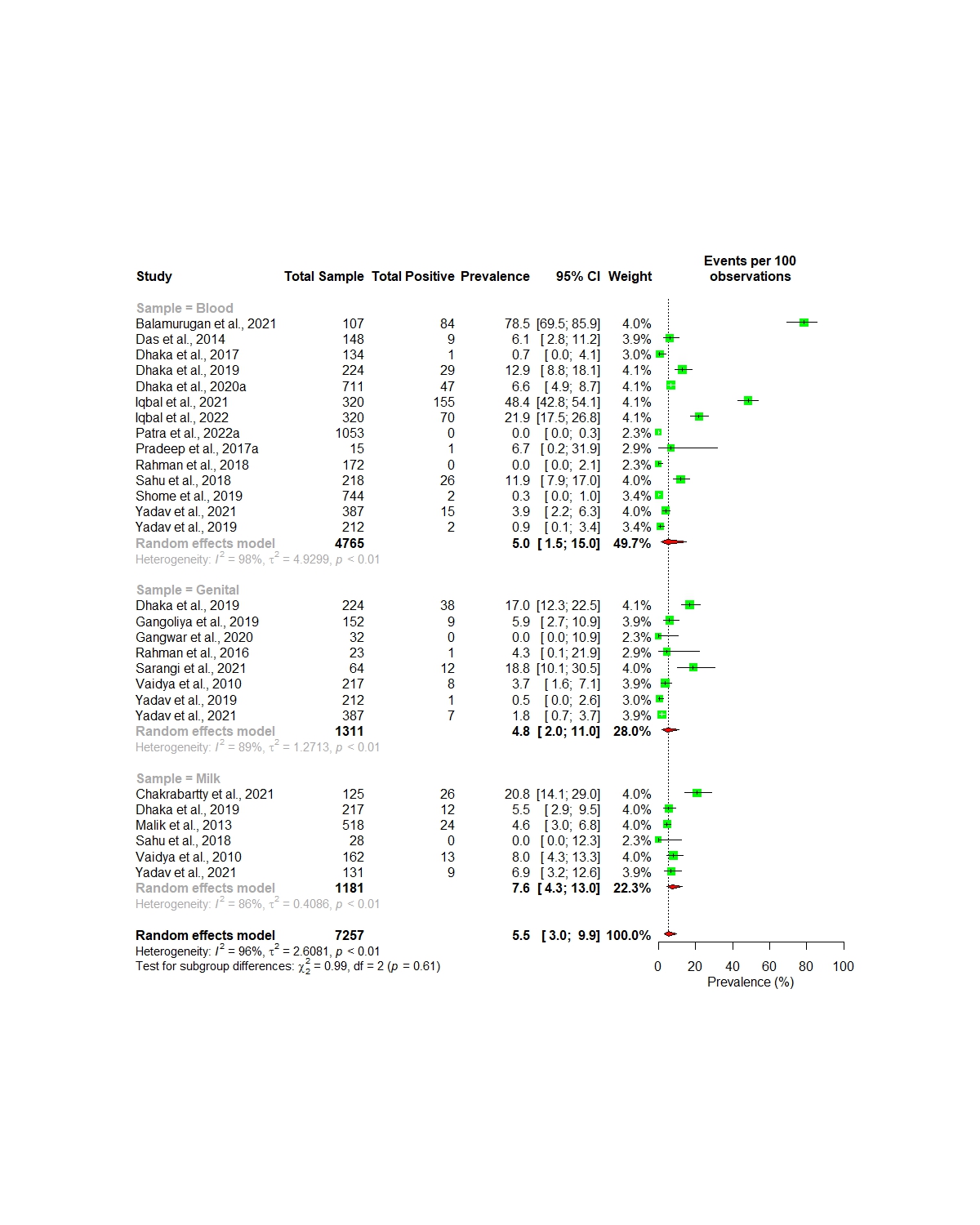


#### Individual carrier country


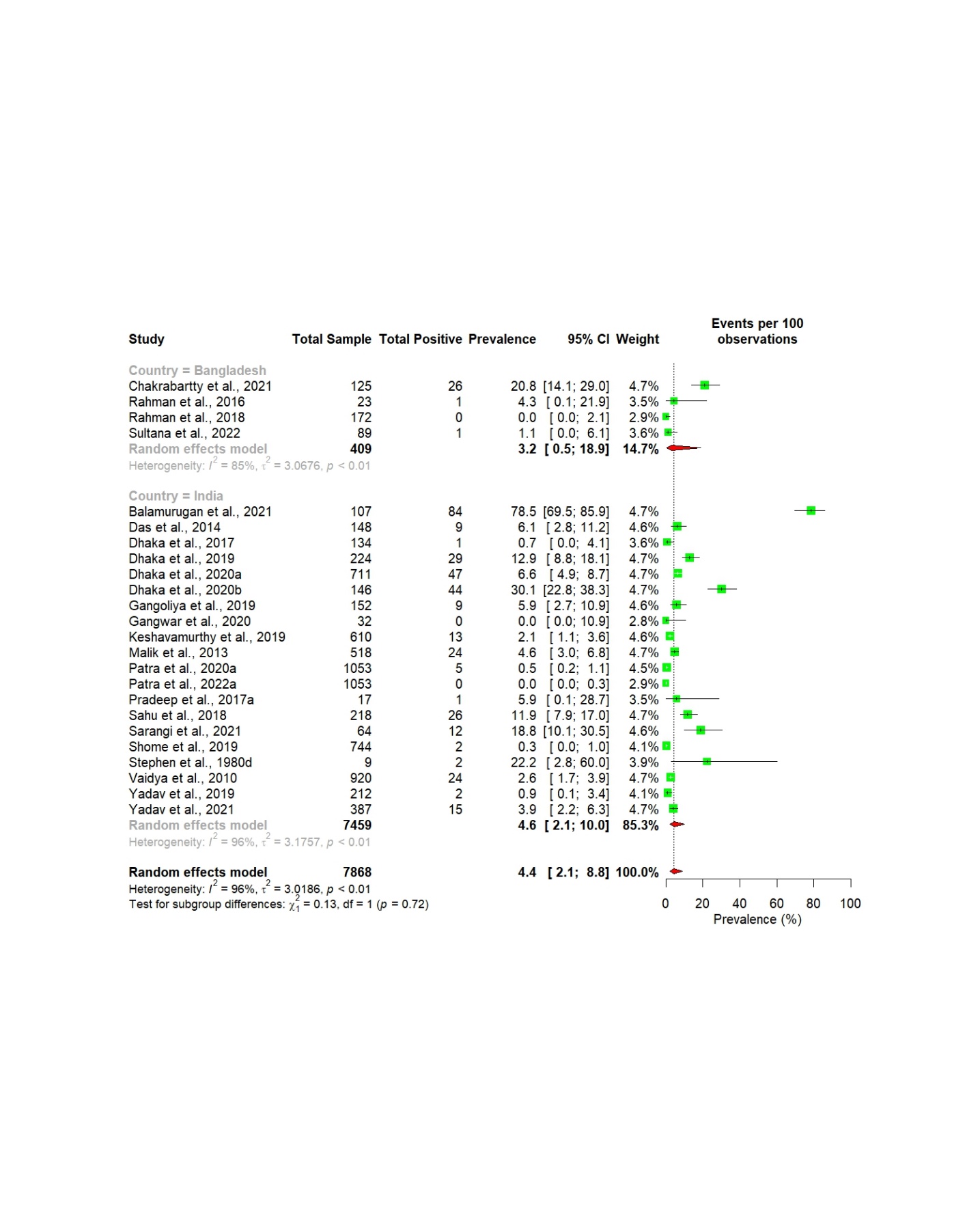


### Antibody_milk


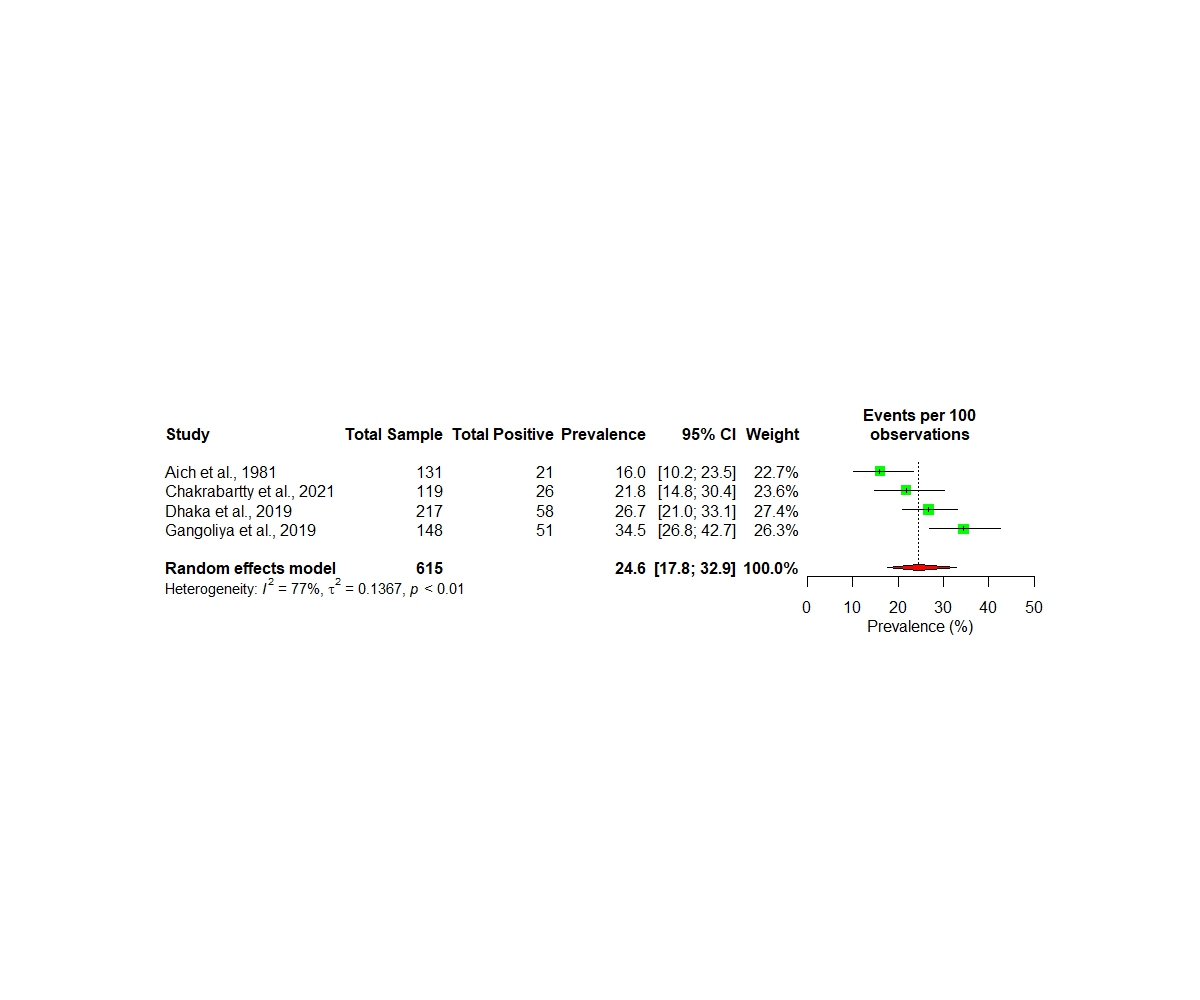


## Coxiellosis in other animals and birds

### Non-ruminant mammals


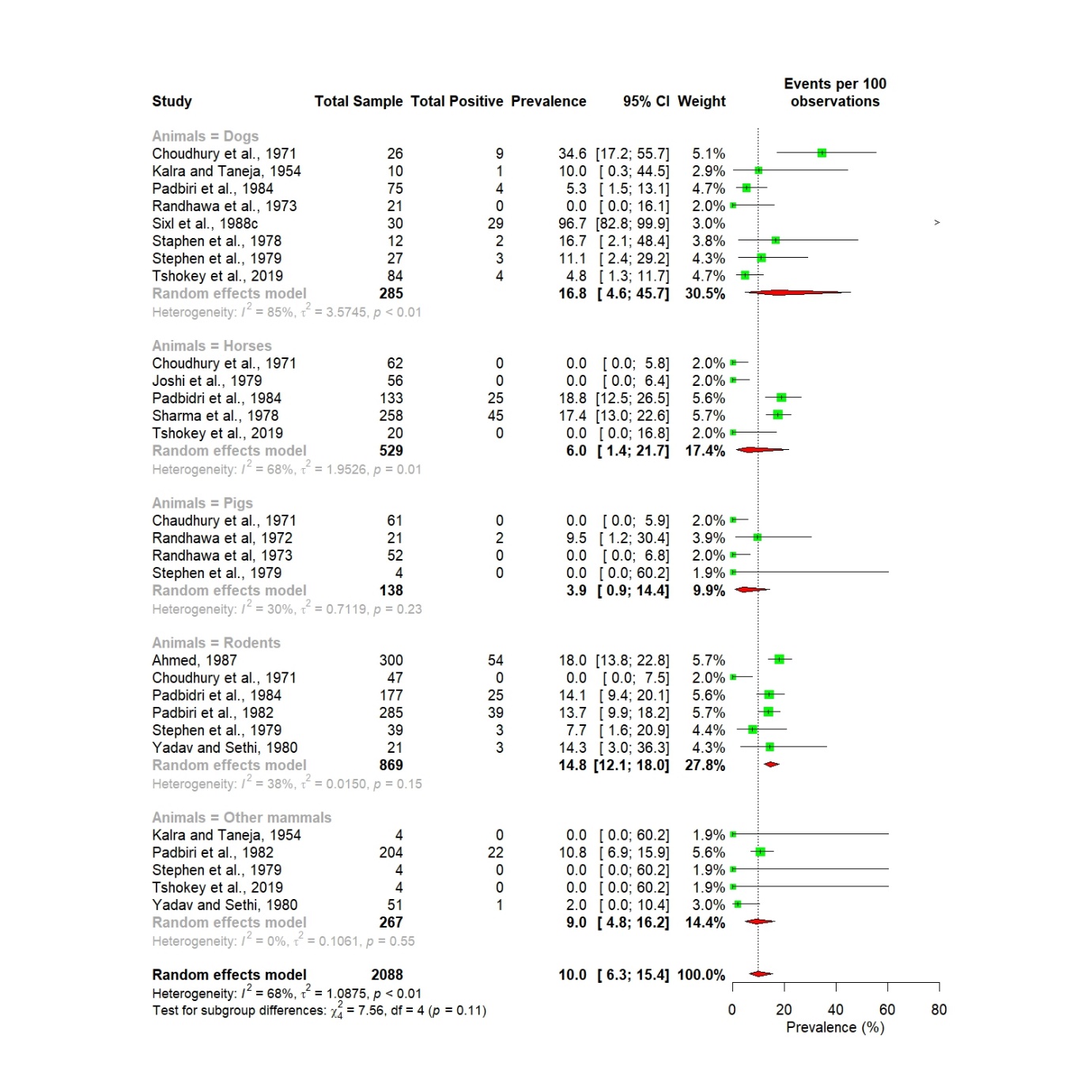


### Non-mammals


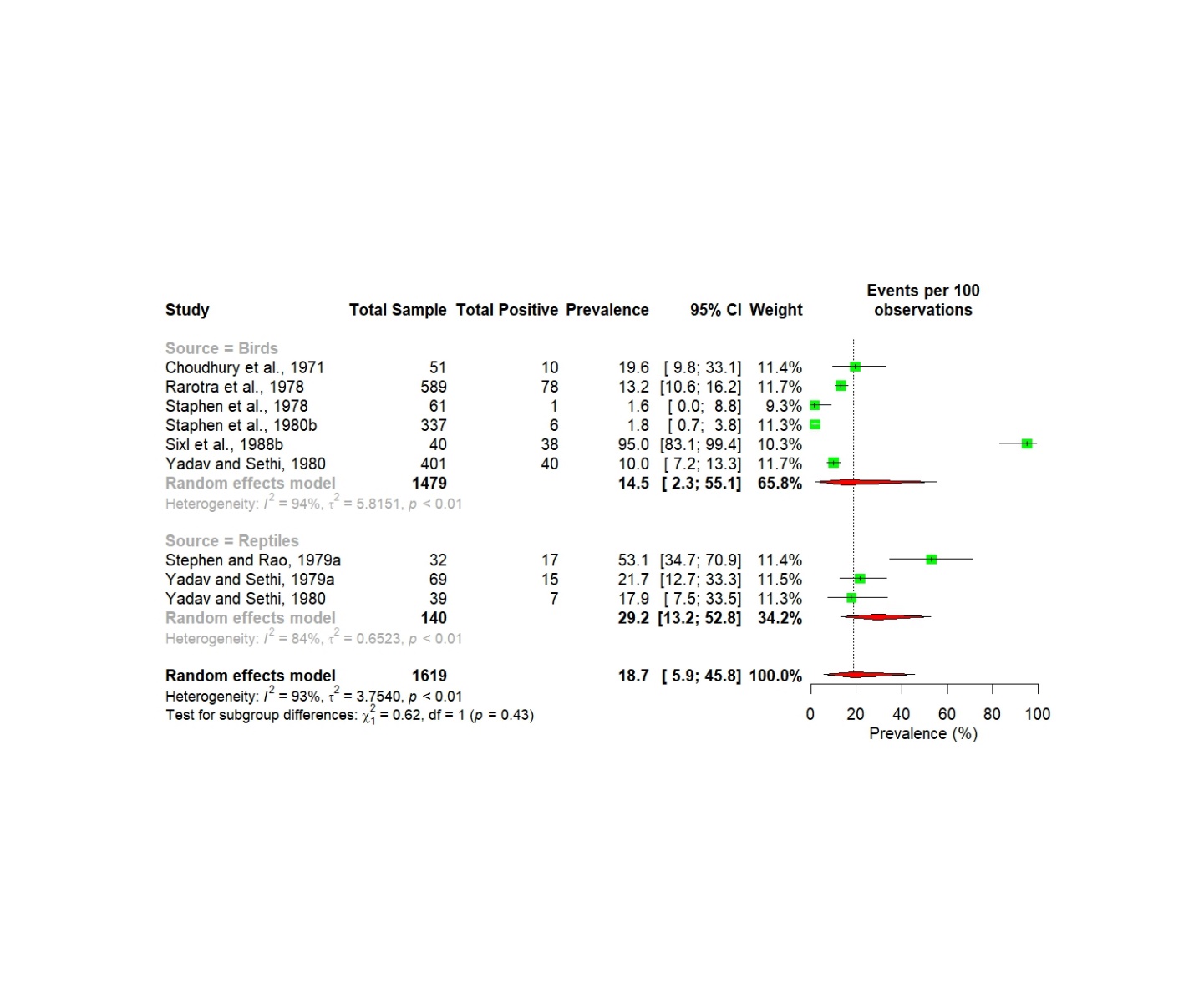


### Environment

#### Carrier prevalence in ticks


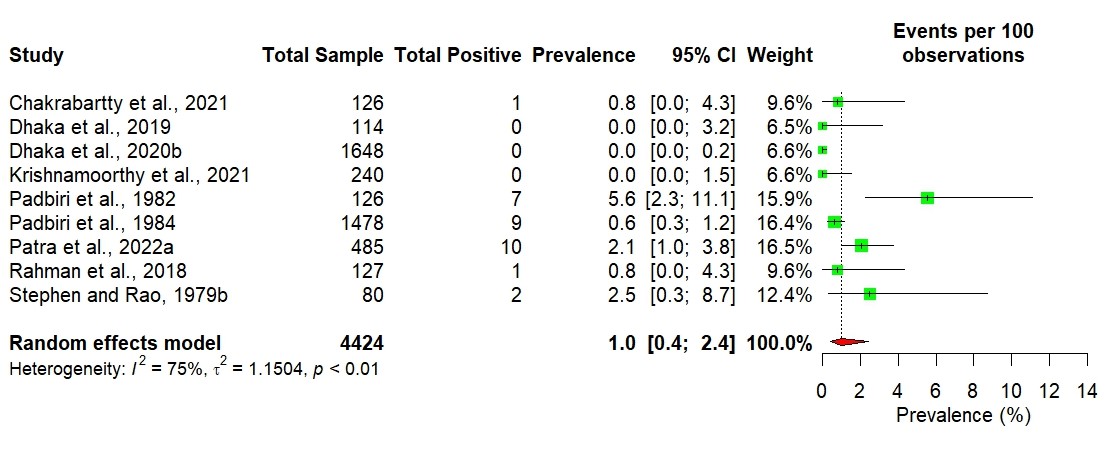


#### Environment samples


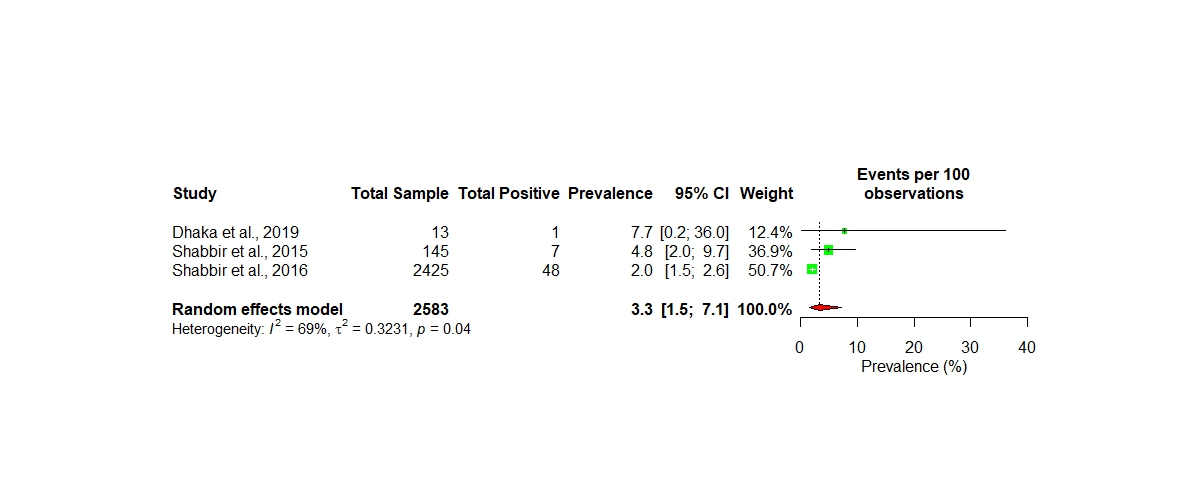

Supplement: Supporting Information 6 — Forest plots of meta-analysis depict the seroprevalence and carrier prevalence of Coxiellosis in humans and animals of the South Asian countries. [file 2890693.f6.docx]
